# Supplementary material for: Somatic PIK3R1 mutations in the iSH2 domain are accessible to PI3Kα inhibition
Source: EMBO Mol Med. 2025 May 19;17(7):1556–74. doi: 10.1038/s44321-025-00249-9 (PMC12254339; doi:10.1038/s44321-025-00249-9)
Supplement: Supplementary file 1 — Table EV1 [file 44321_2025_249_MOESM1_ESM.docx]

| Gene/Variant | ID | Name | Size (bp) | Promoter | ORF | Marker |
| --- | --- | --- | --- | --- | --- | --- |
| GFP | VB210708-1118hgr | pRP[Exp]-{EF1A/T7}>EGFP | 4284 | {EF1A/T7} | EGFP | EGFP |
| hPIK3R1 WT | VB220330-1182cfm | pRP[Exp]-EGFP-EF1A>hPIK3R1[NM_181523.3] | 7312 | EF1A | hPIK3R1[NM_181523.3]* | EGFP |
| hPIK3R1 p.(Q579-Y580)del | VB220330-1186cmc | pRP[Exp]-EGFP-EF1A>hPIK3R1[NM_181523.3]* | 7306 | EF1A | hPIK3R1[NM_181523.3]* | EGFP |
| hPIK3R1 p.K459dup | VB220330-1180ueb | pRP[Exp]-EGFP-EF1A>hPIK3R1[NM_181523.3]* | 7315 | EF1A | hPIK3R1[NM_181523.3]* | EGFP |
| hPIK3R1 WT | VB211115-1274xvk | pRP[Exp]-{EF1A/T7}>hPIK3R1[NM_181523.3] | 5739 | {EF1A/T7} | hPIK3R1[NM_181523.3] | None |
| hPIK3R1 p.K567E | VB211115-1275qgv | pRP[Exp]-{EF1A/T7}>{hPIK3R1[NM_181523.3](K567E)} | 5739 | {EF1A/T7} | hPIK3R1[NM_181523.3]* | None |
| hPIK3R1 p.568L | VB211125-1031wsr | pRP[Exp]-{EF1A/T7}>{hPIK3R1[NM_181523.3](P568L)} | 5739 | {EF1A/T7} | hPIK3R1[NM_181523.3]* | None |
| hPIK3R1 p.(K567E,P568L) | VB211125-1034sup | pRP[Exp]-{EF1A/T7}>{hPIK3R1[NM_181523.3](K567E,P568L)} | 5739 | {EF1A/T7} | hPIK3R1[NM_181523.3]* | None |

**Table EV1**: Plasmids used in this study.

All plasmids were mammalian gene expression vectors designed for high copy number from the VectorBuilder UltraStable cloning host added with a sequence coding for ampicillin resistance. When two promoters are present, they respectively control the expression of the ORF and that of the marker if present.

* indicates that a modification was made to the deposited sequence. EF1A, Elongation factor 1-alpha 1. EGFP, Enhanced GFP. GFP, Green fluorescent protein. ORF, open reading frame.
