# Supplementary material for: Somatic PIK3R1 mutations in the iSH2 domain are accessible to PI3Kα inhibition
Source: EMBO Mol Med. 2025 May 19;17(7):1556–74. doi: 10.1038/s44321-025-00249-9 (PMC12254339; doi:10.1038/s44321-025-00249-9)

Figure 1B – Blot – p85

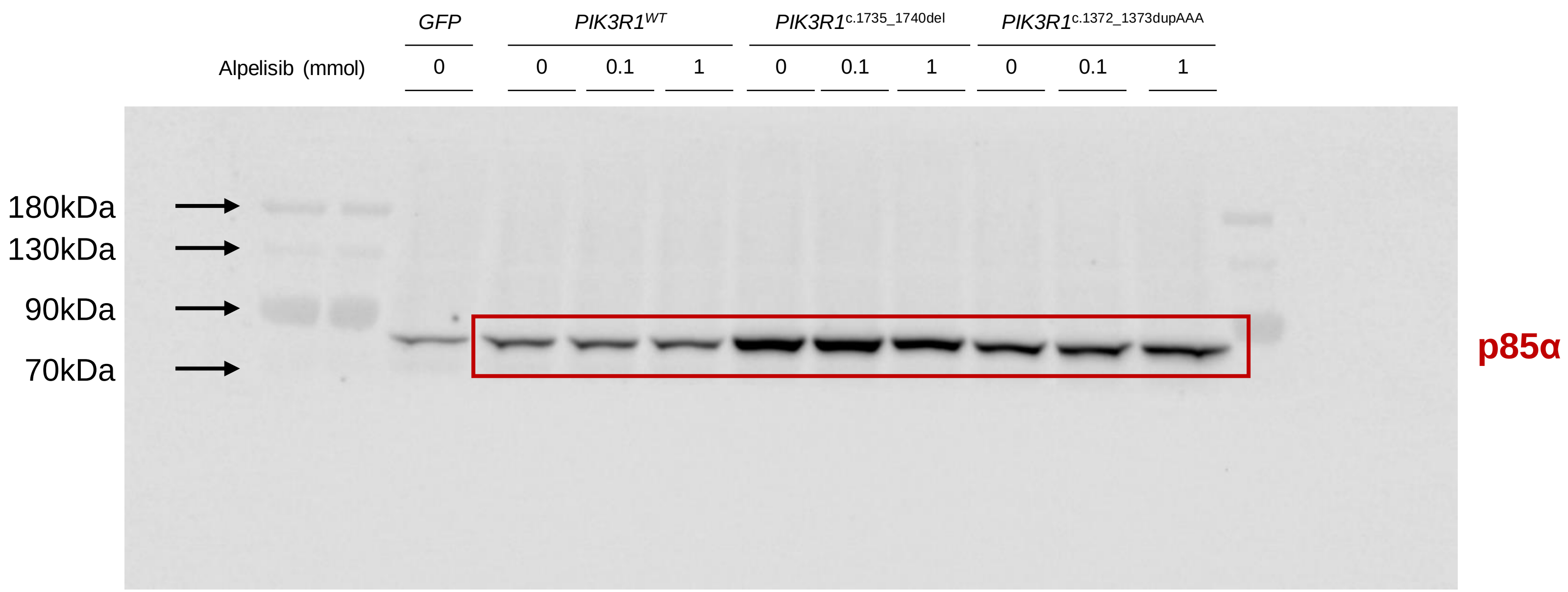

Figure 1B – Blot – P-AKT

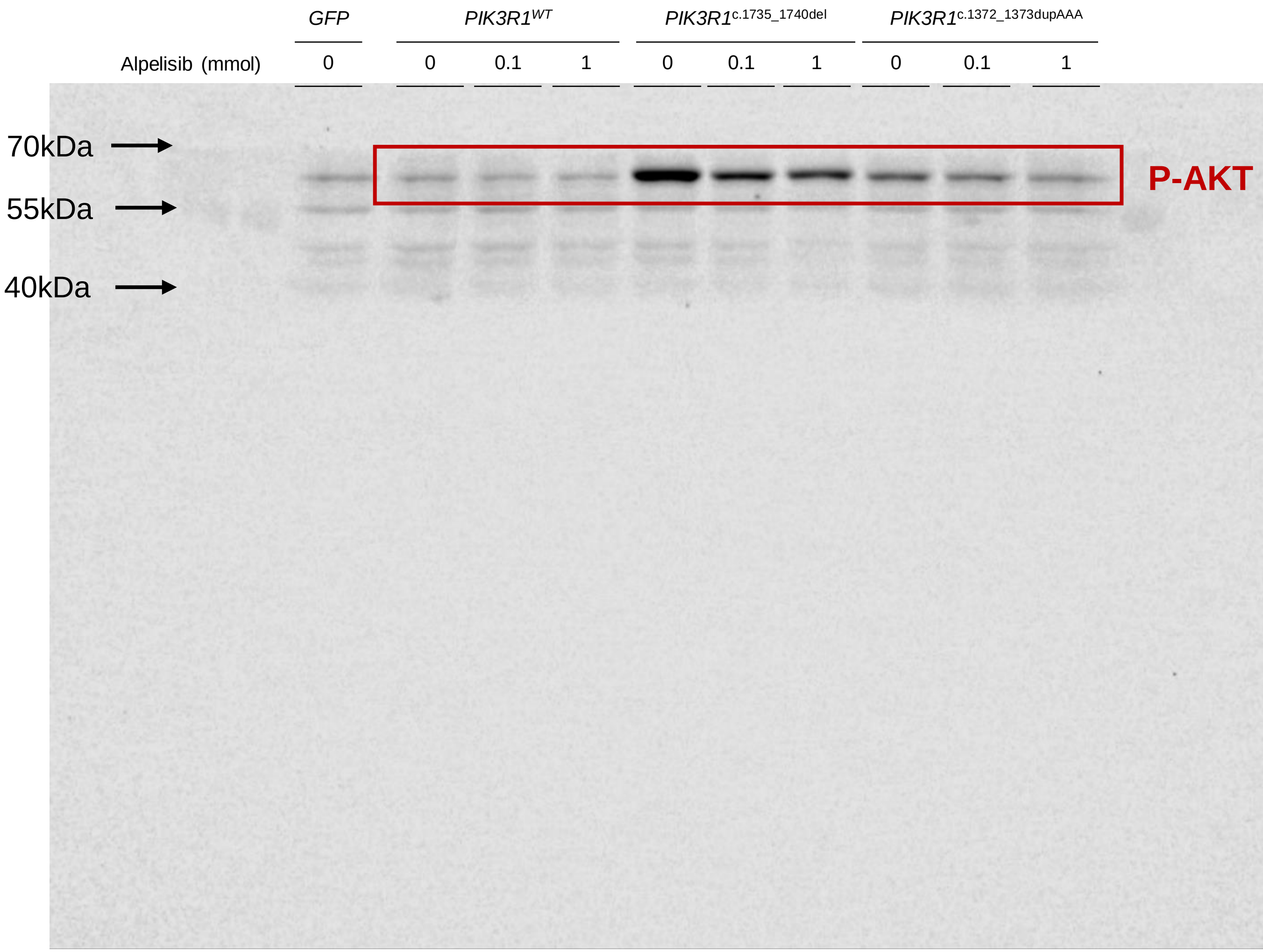

Figure 1B – Blot – AKT

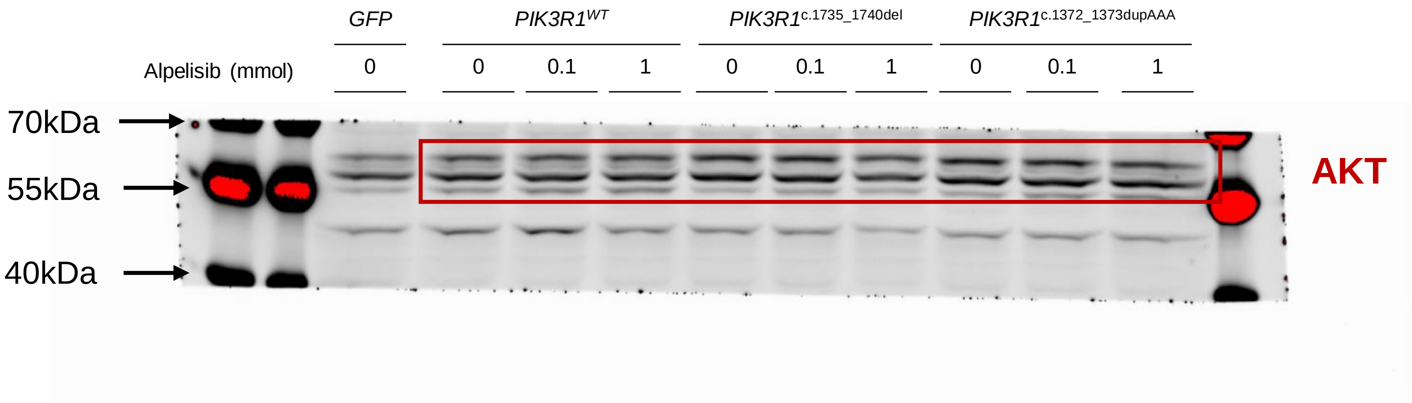

Figure 1B – Blot – P-S6RP

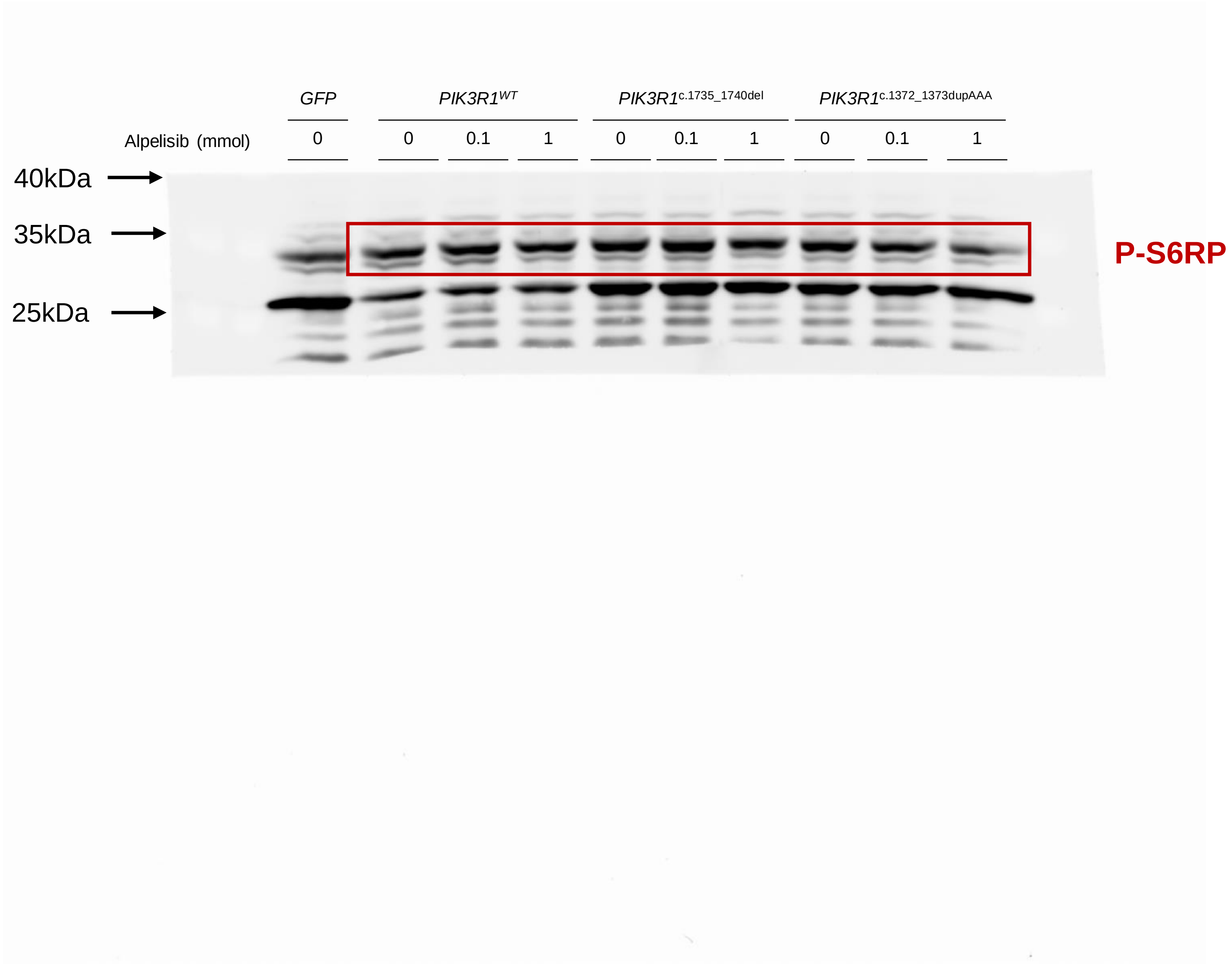

Figure 1B – Blot – S6RP

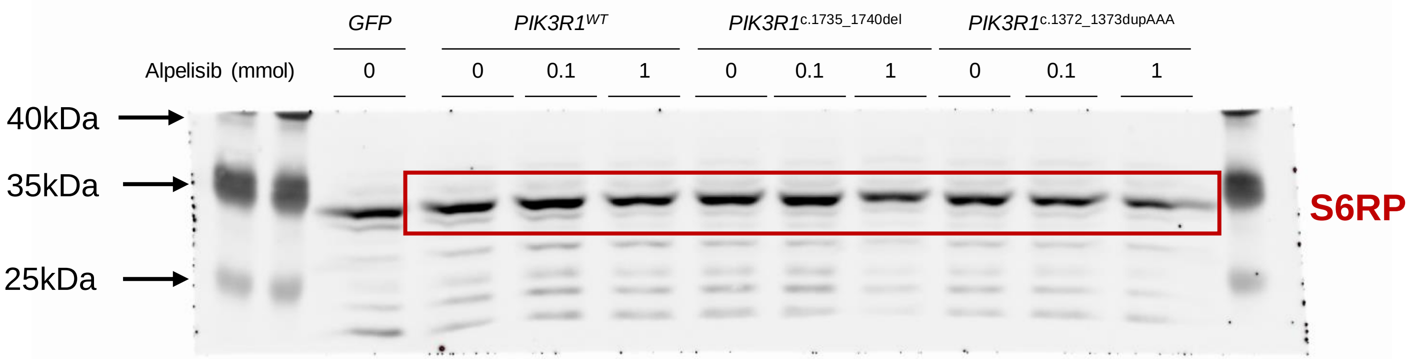

Figure 1B – Blot – α-tubulin

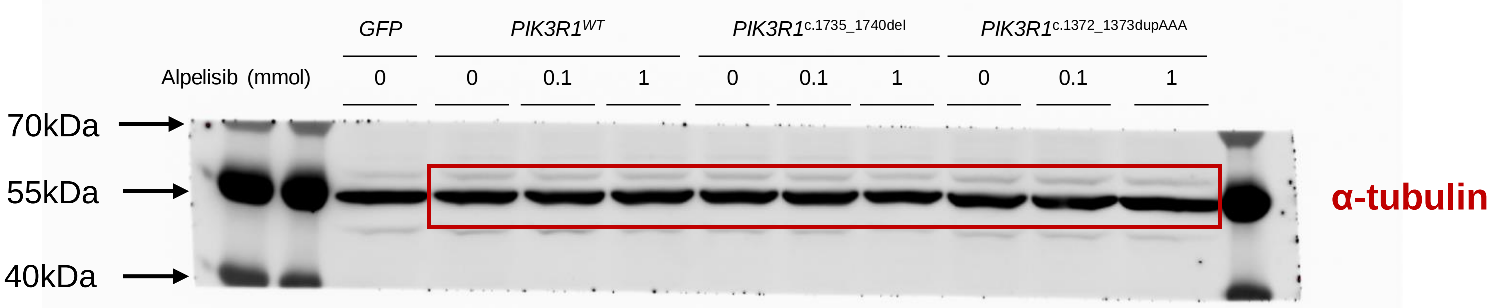

Supplement: Supplementary file 3 — Source data Fig. 1 [file 44321_2025_249_MOESM3_ESM.zip › Figure 1/1B_blot_summary.pdf]
