# Supplementary figures and images for: Somatic PIK3R1 mutations in the iSH2 domain are accessible to PI3Kα inhibition
Source: EMBO Mol Med. 2025 May 19;17(7):1556–74. doi: 10.1038/s44321-025-00249-9 (PMC12254339; doi:10.1038/s44321-025-00249-9)

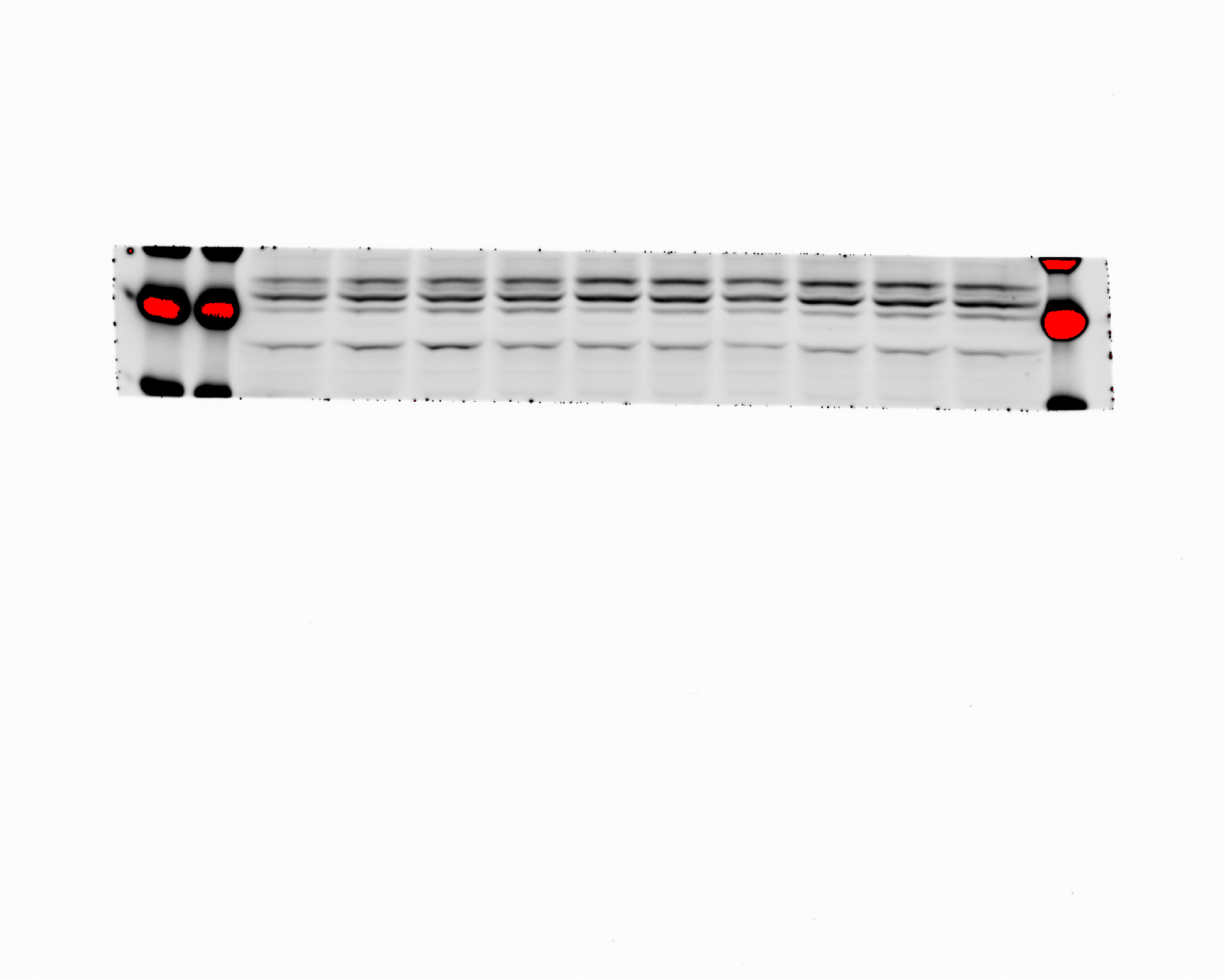

Supplement: Supplementary file 3 — Source data Fig. 1 [file 44321_2025_249_MOESM3_ESM.zip › Figure 1/1B_blot_akt.tif]

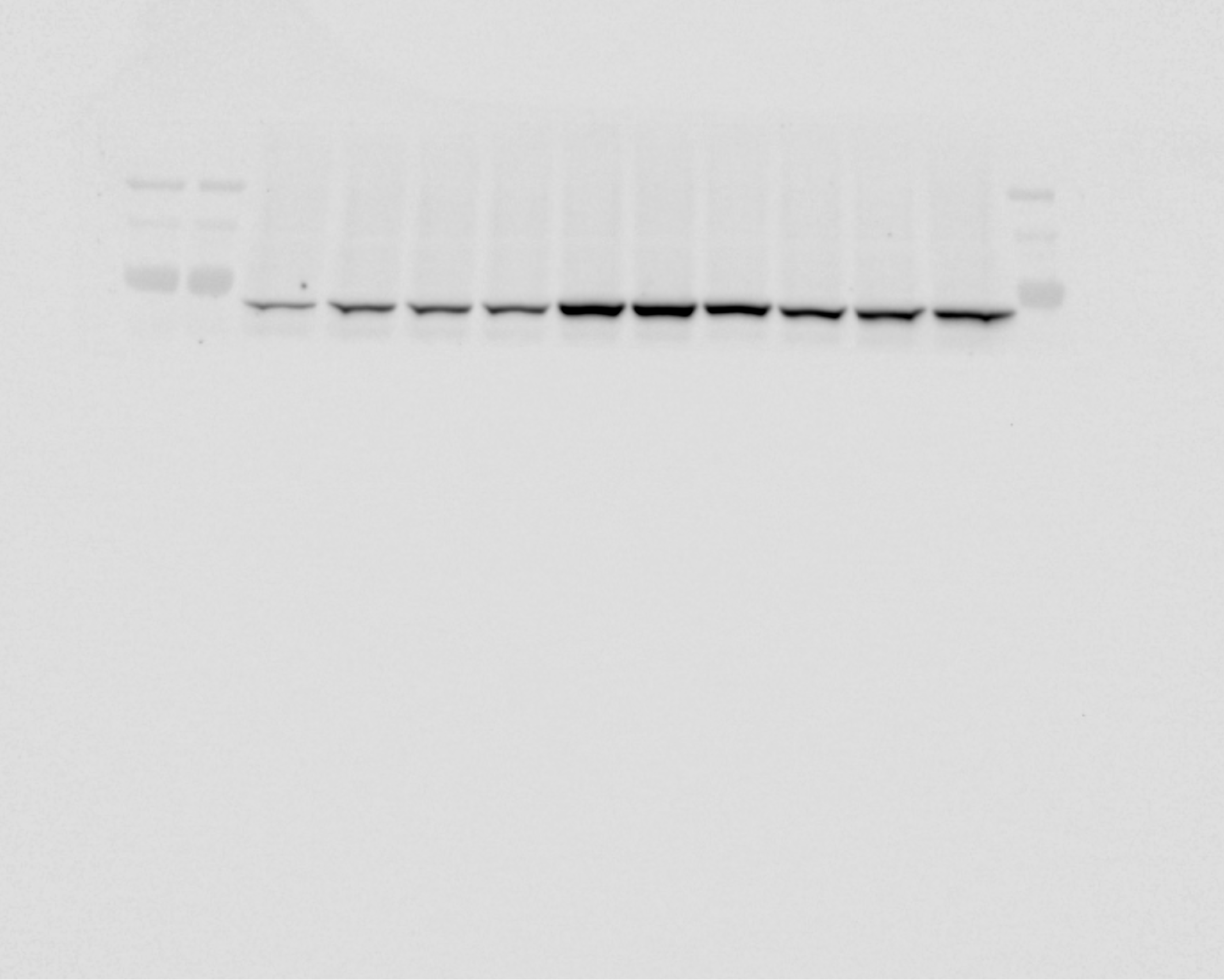

Supplement: Supplementary file 3 — Source data Fig. 1 [file 44321_2025_249_MOESM3_ESM.zip › Figure 1/1B_blot_p85.tif]

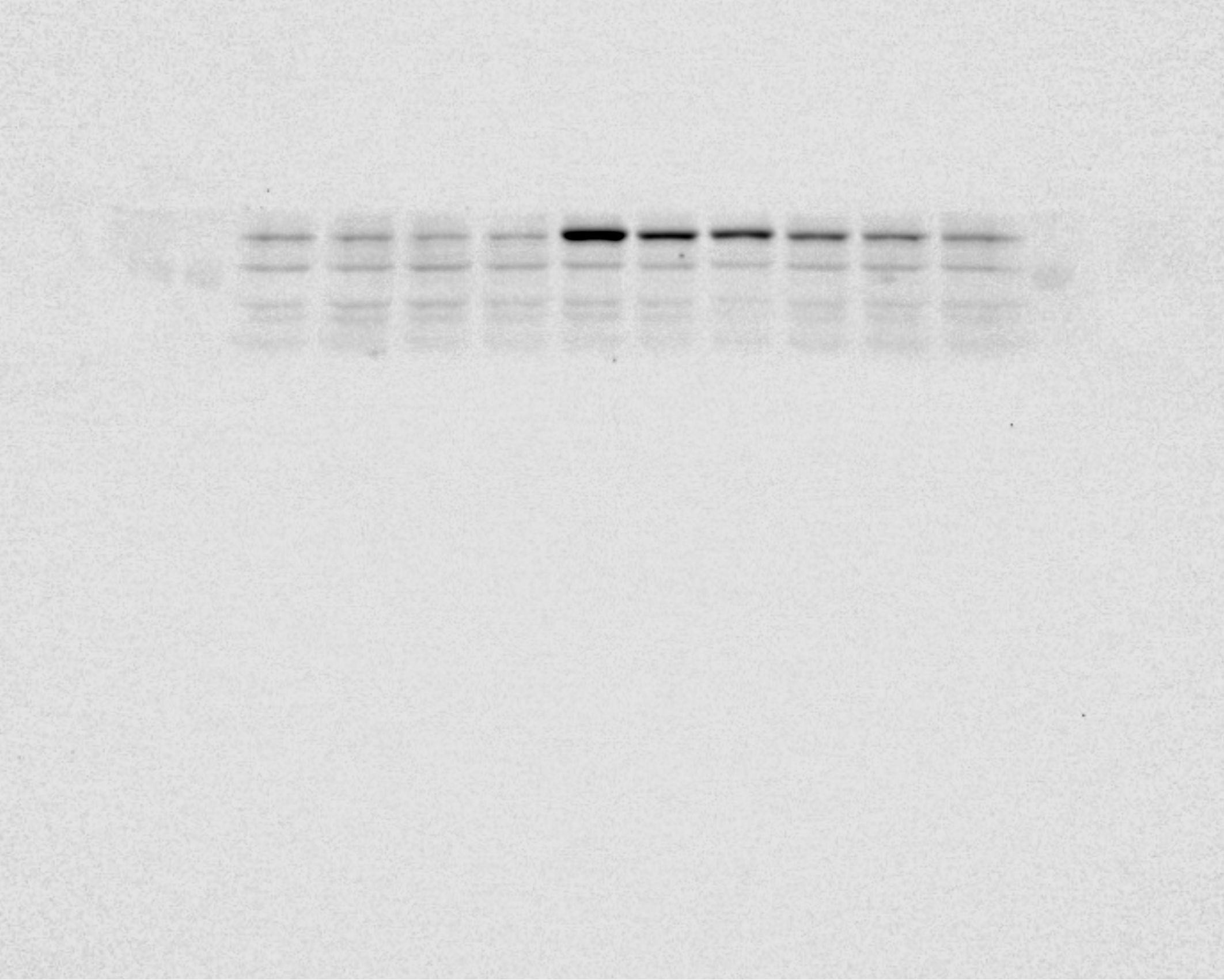

Supplement: Supplementary file 3 — Source data Fig. 1 [file 44321_2025_249_MOESM3_ESM.zip › Figure 1/1B_blot_p_akt.tif]

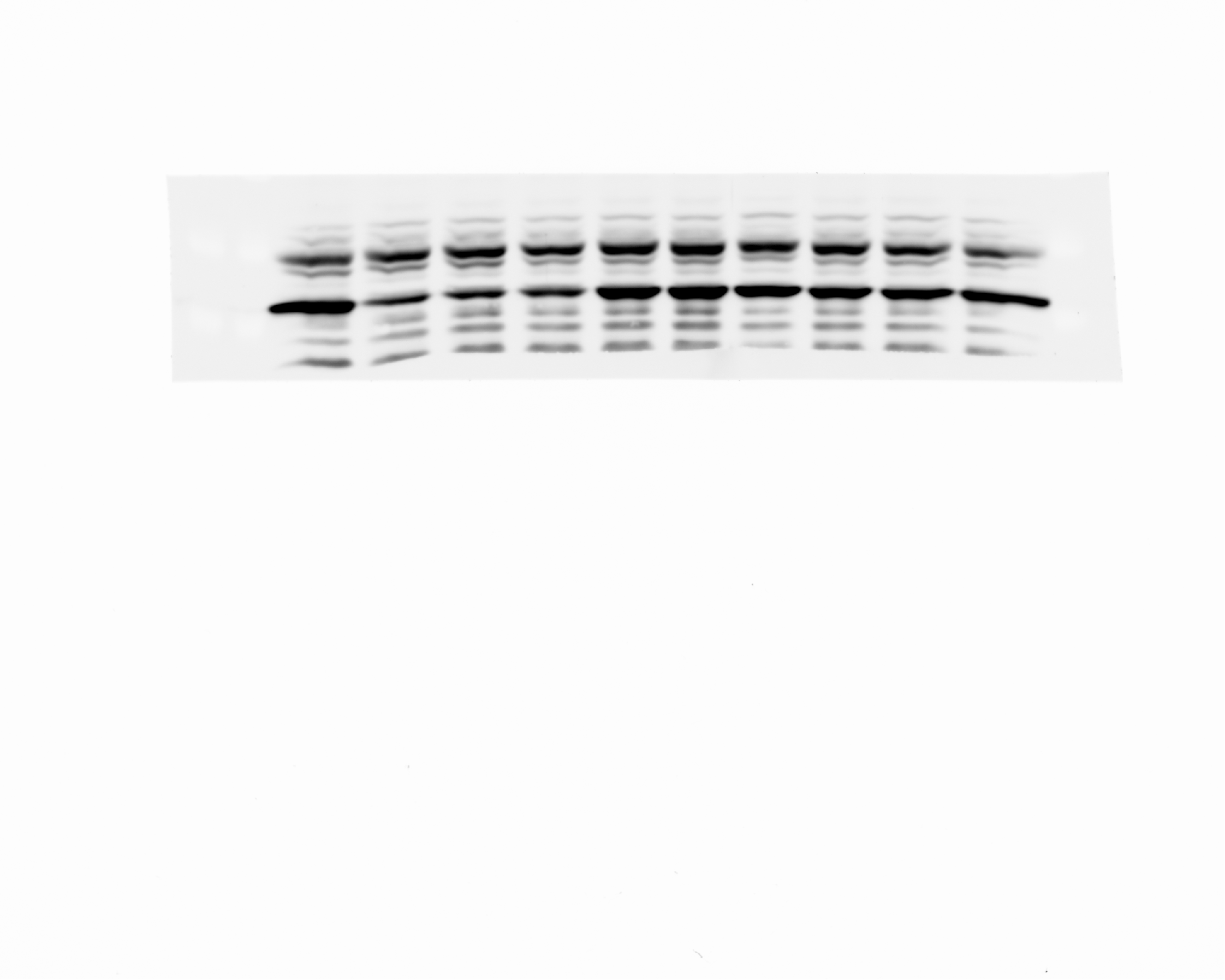

Supplement: Supplementary file 3 — Source data Fig. 1 [file 44321_2025_249_MOESM3_ESM.zip › Figure 1/1B_blot_p_s6rp.tif]

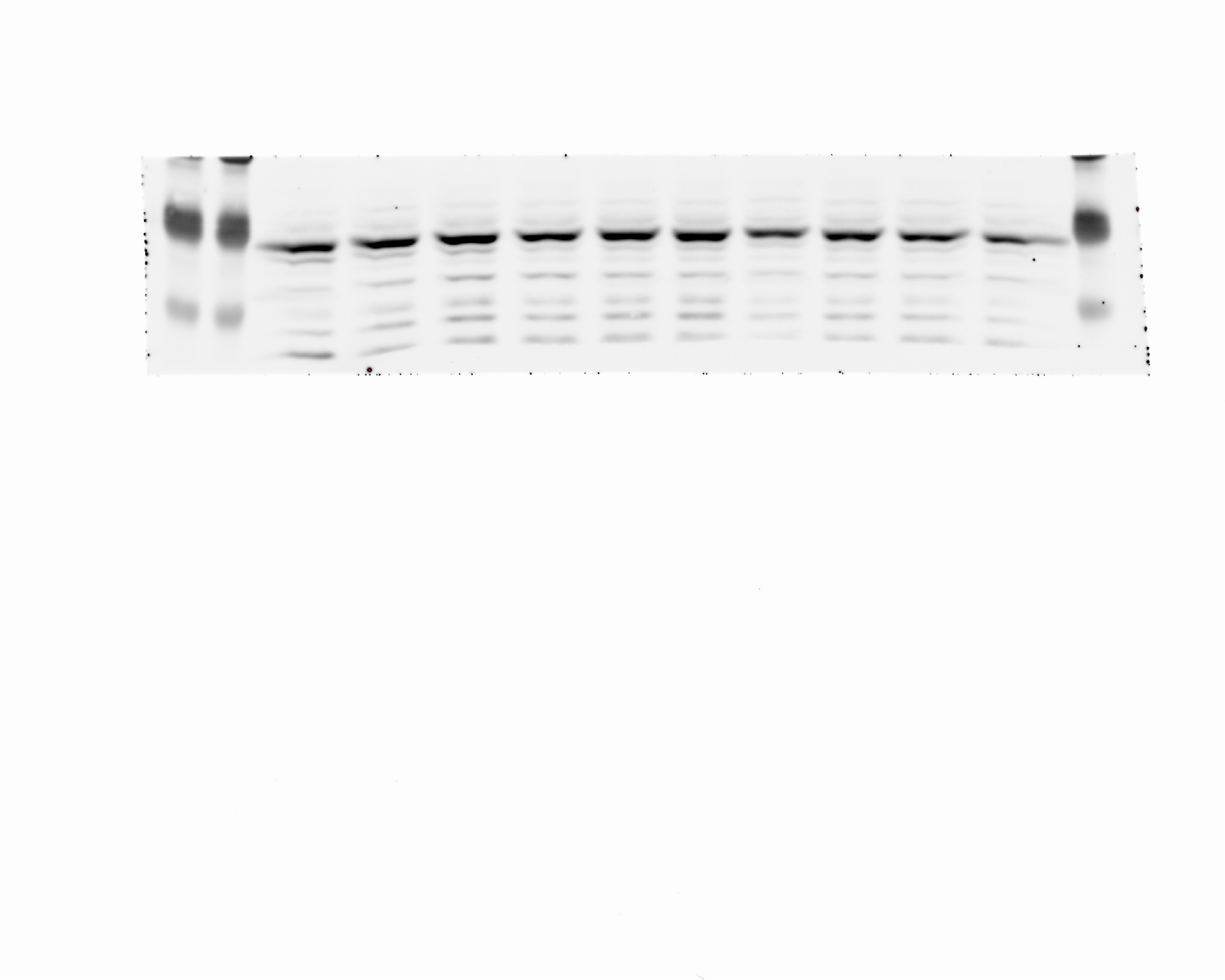

Supplement: Supplementary file 3 — Source data Fig. 1 [file 44321_2025_249_MOESM3_ESM.zip › Figure 1/1B_blot_s6rp.tif]

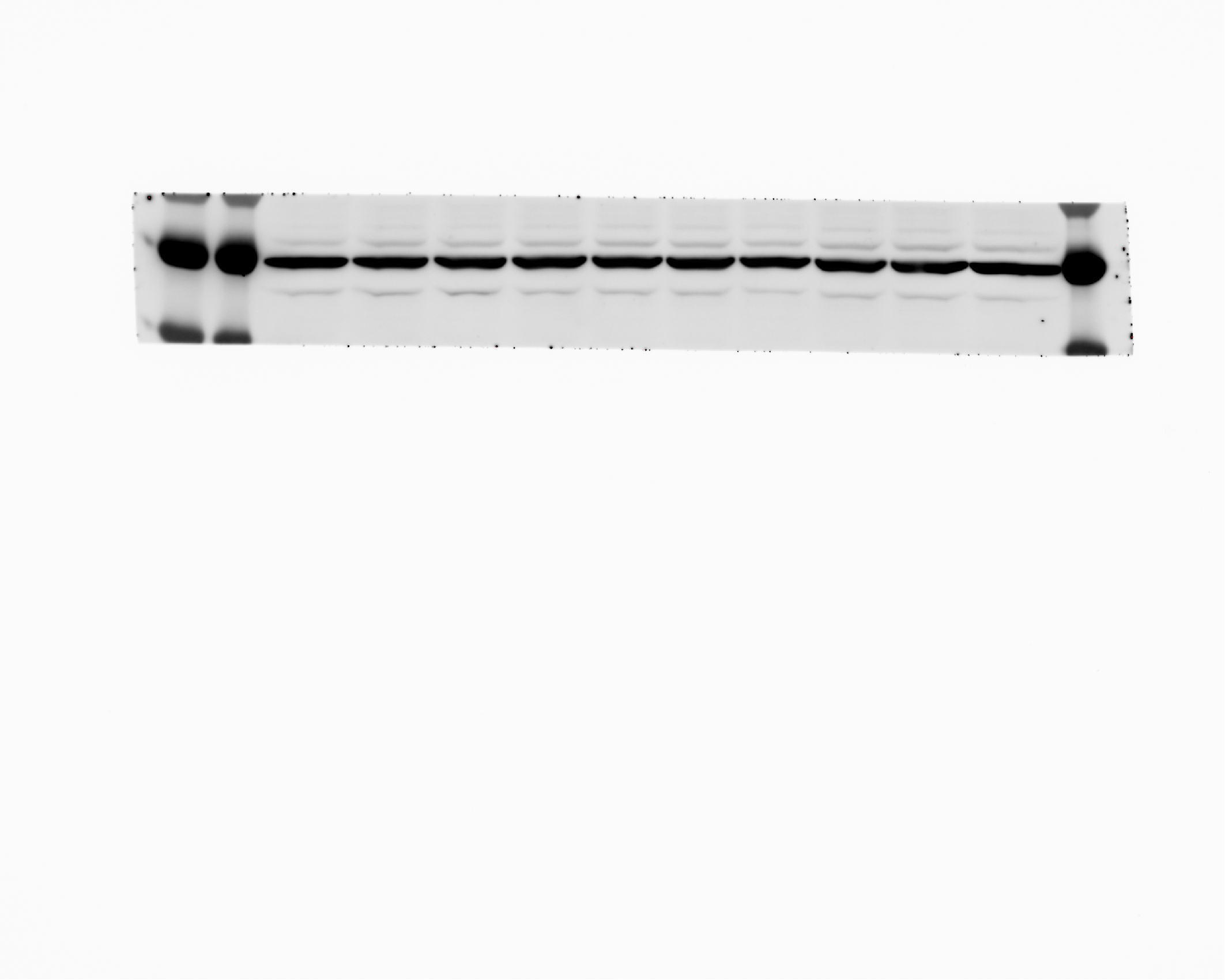

Supplement: Supplementary file 3 — Source data Fig. 1 [file 44321_2025_249_MOESM3_ESM.zip › Figure 1/1B_blot_tubulin.tif]

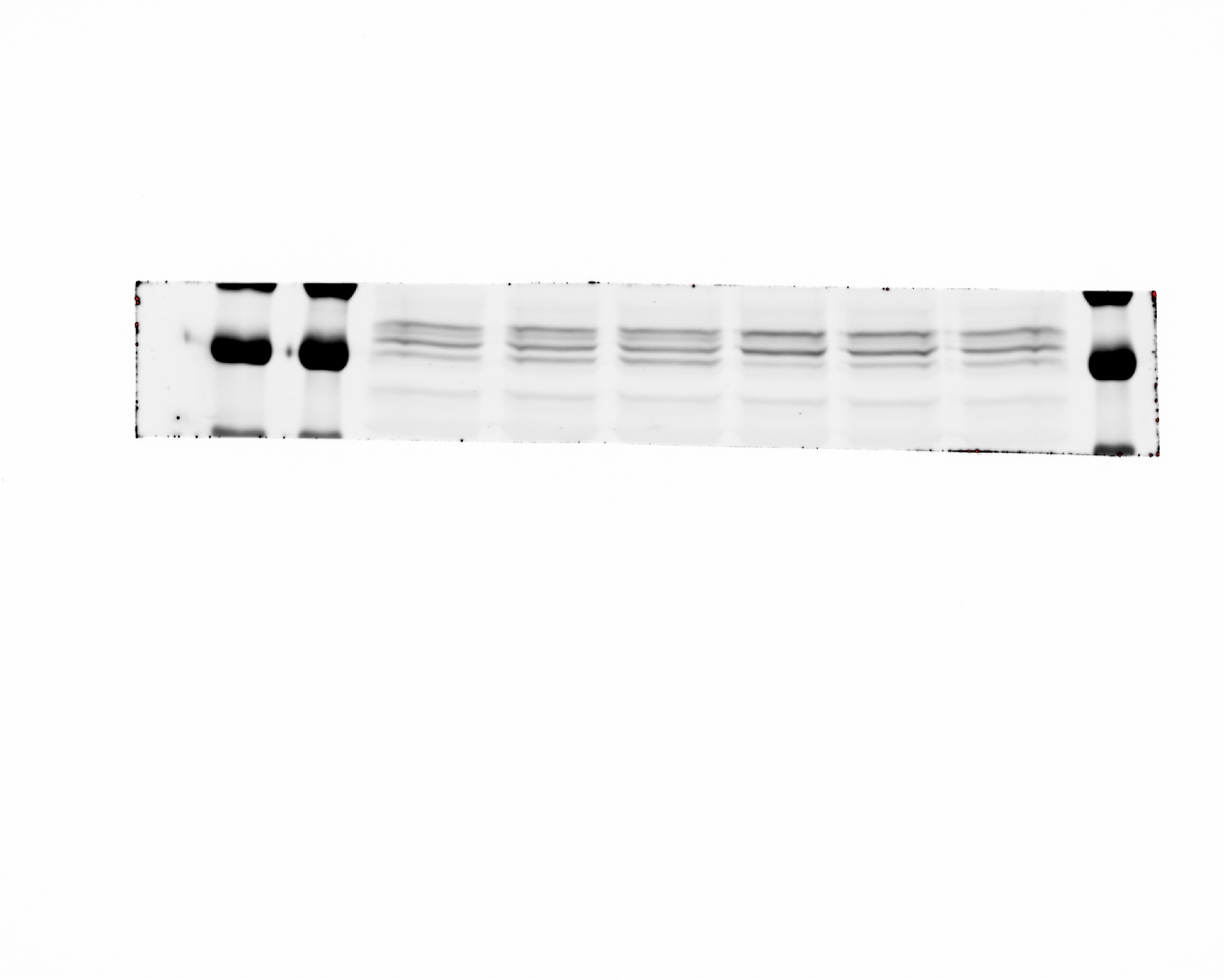

Supplement: Supplementary file 3 — Source data Fig. 1 [file 44321_2025_249_MOESM3_ESM.zip › Figure 1/1C_blot_akt.tif]

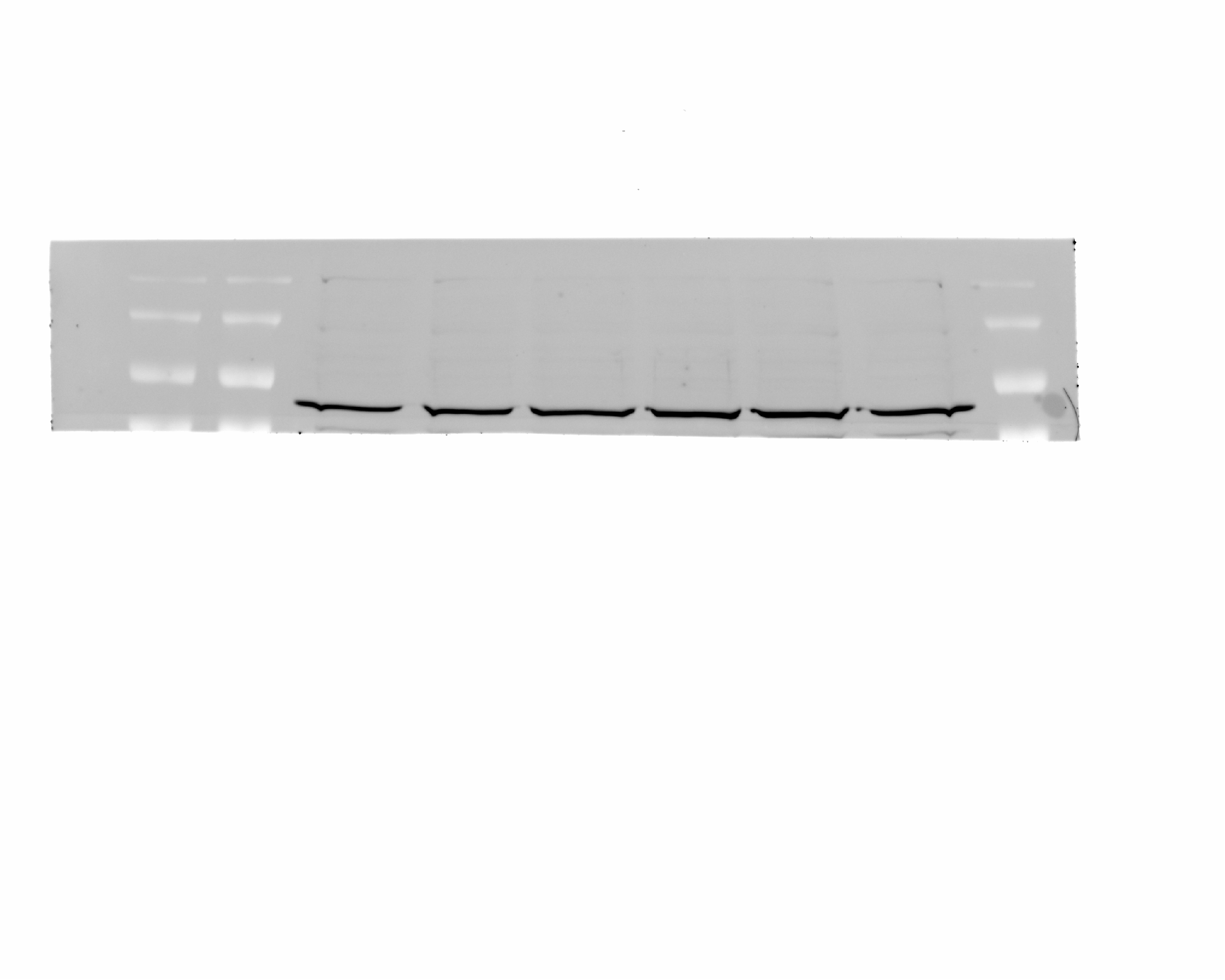

Supplement: Supplementary file 3 — Source data Fig. 1 [file 44321_2025_249_MOESM3_ESM.zip › Figure 1/1C_blot_p85.tif]

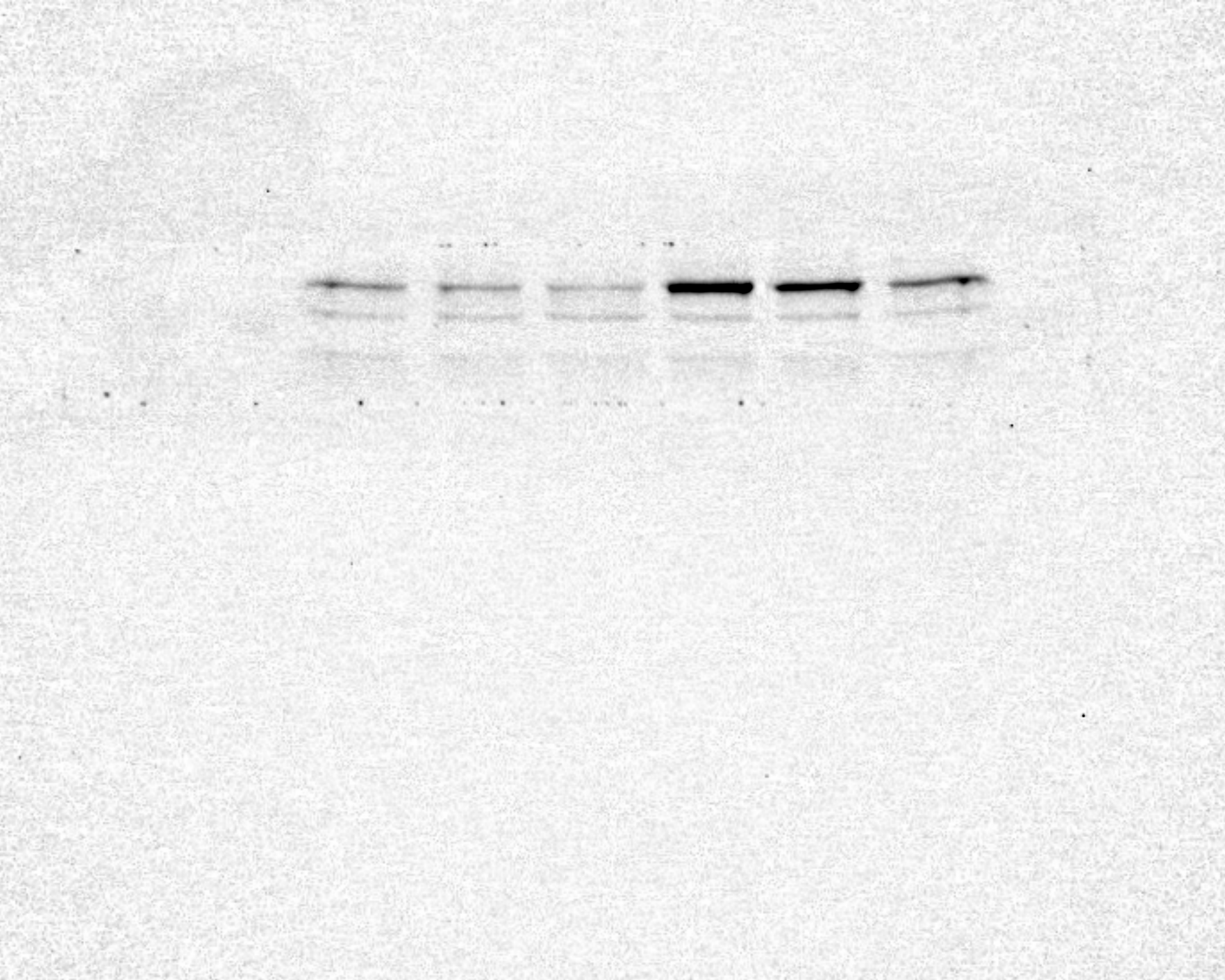

Supplement: Supplementary file 3 — Source data Fig. 1 [file 44321_2025_249_MOESM3_ESM.zip › Figure 1/1C_blot_p_akt.tif]

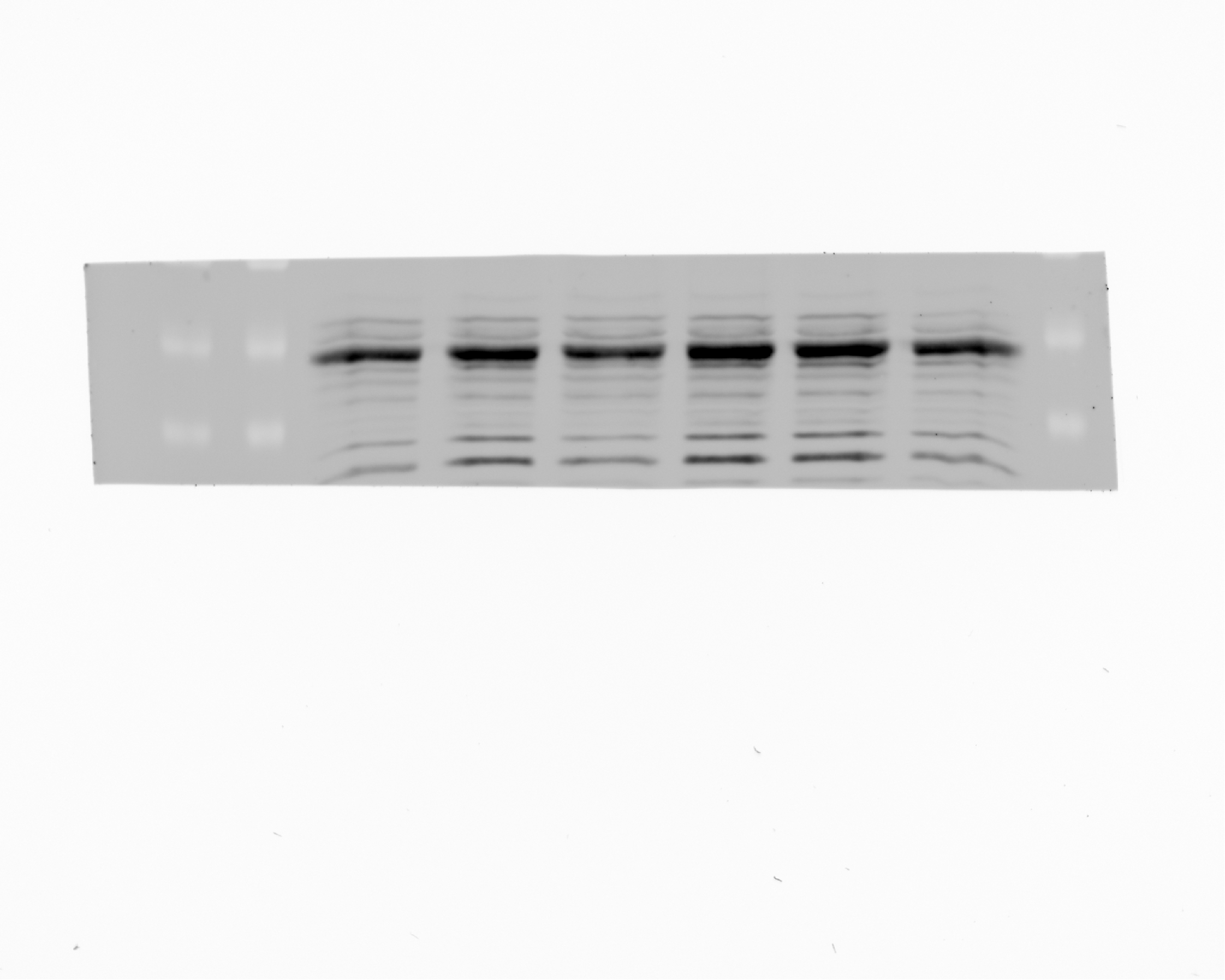

Supplement: Supplementary file 3 — Source data Fig. 1 [file 44321_2025_249_MOESM3_ESM.zip › Figure 1/1C_blot_p_s6rp.tif]

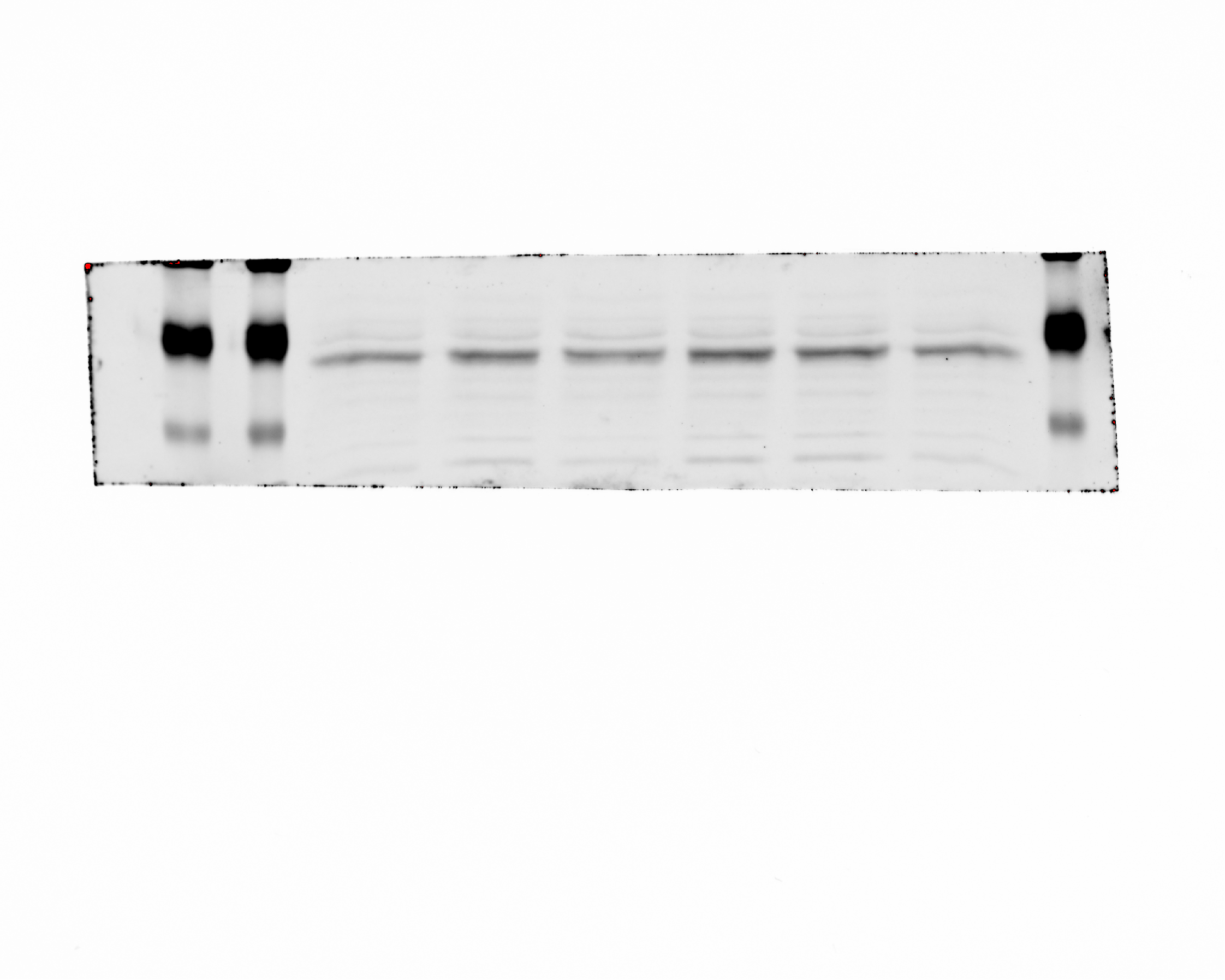

Supplement: Supplementary file 3 — Source data Fig. 1 [file 44321_2025_249_MOESM3_ESM.zip › Figure 1/1C_blot_s6rp.tif]

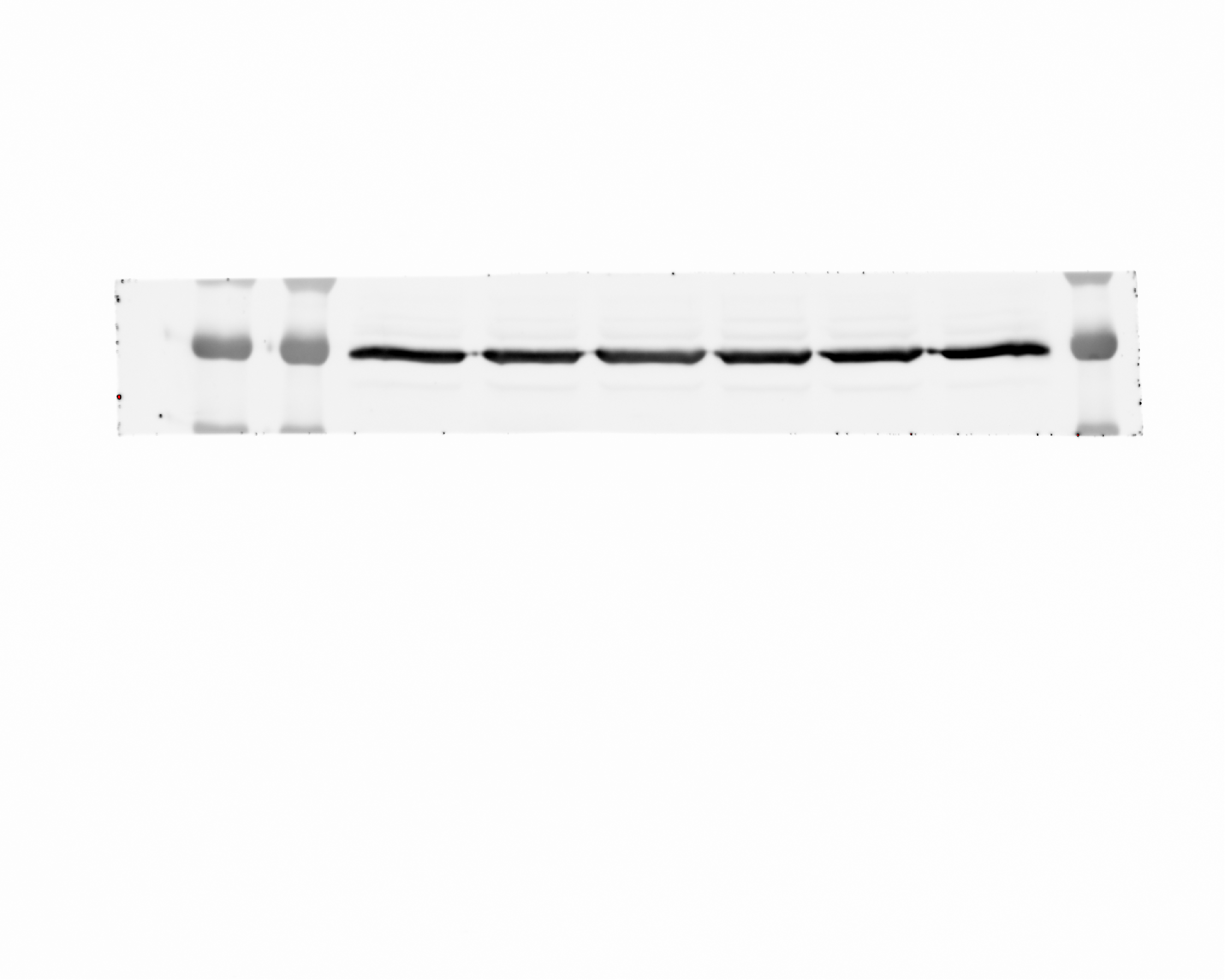

Supplement: Supplementary file 3 — Source data Fig. 1 [file 44321_2025_249_MOESM3_ESM.zip › Figure 1/1C_blot_tubulin.tif]

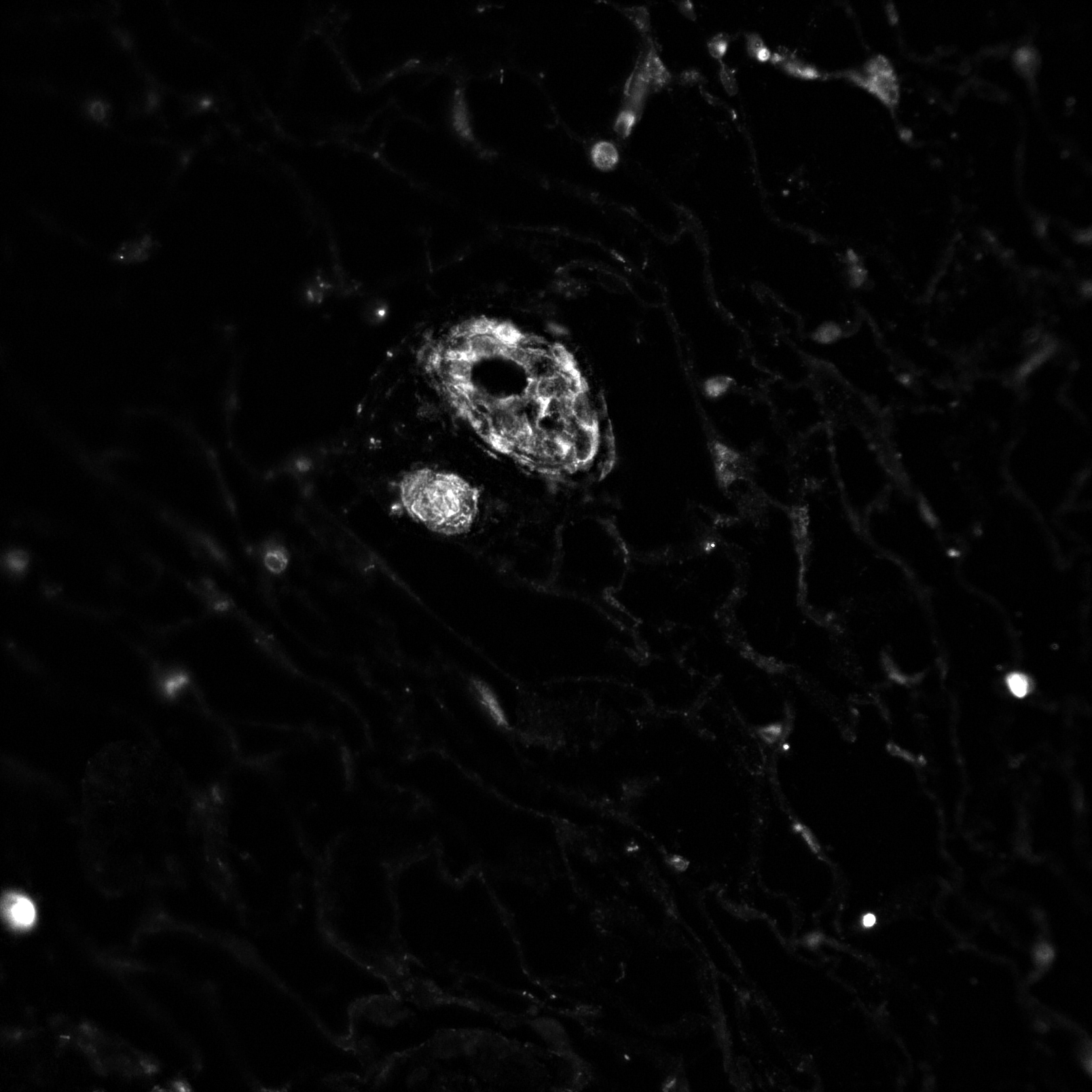

Supplement: Supplementary file 3 — Source data Fig. 1 [file 44321_2025_249_MOESM3_ESM.zip › Figure 1/P-AKT_Panel_1.tif]

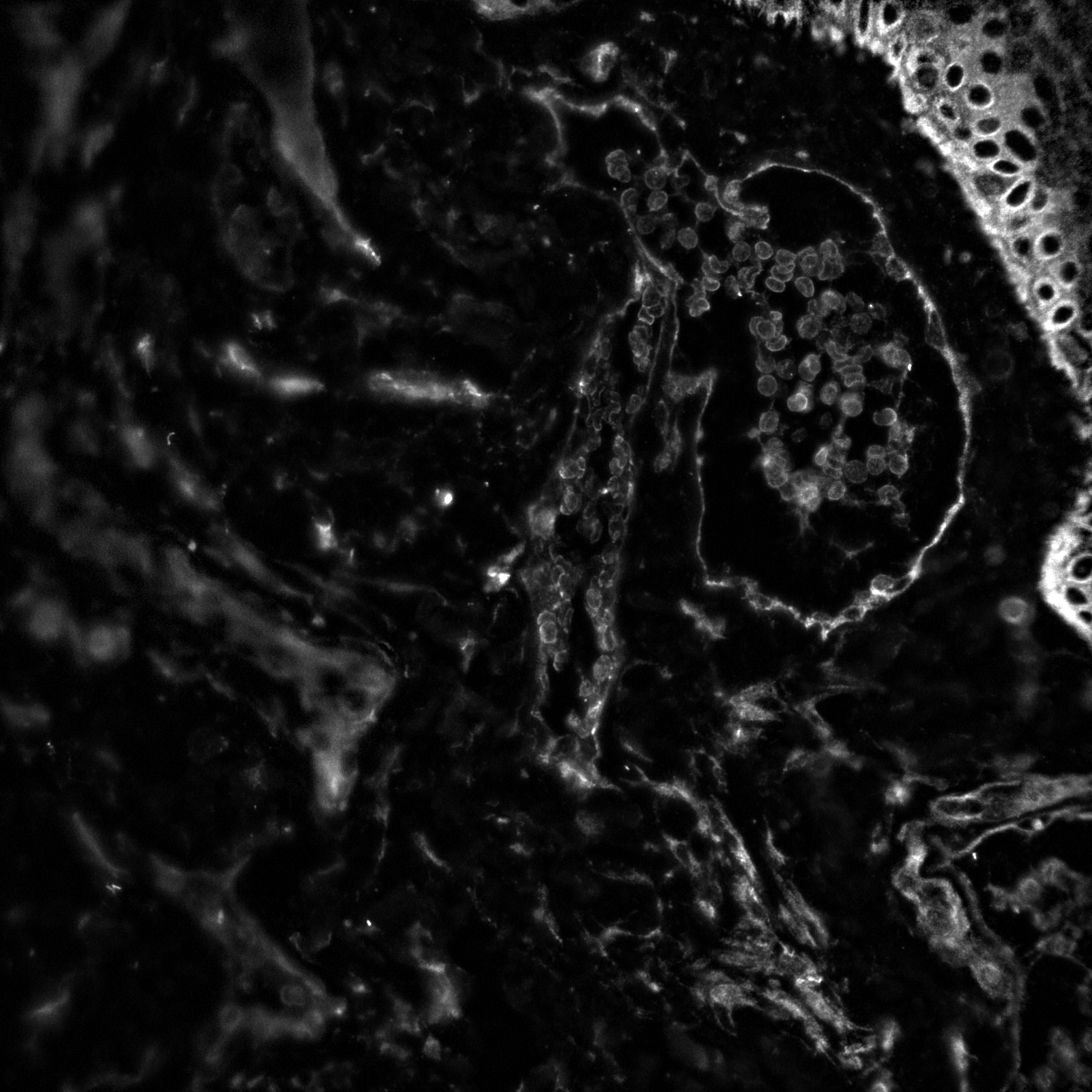

Supplement: Supplementary file 3 — Source data Fig. 1 [file 44321_2025_249_MOESM3_ESM.zip › Figure 1/P-AKT_Panel_2.tif]

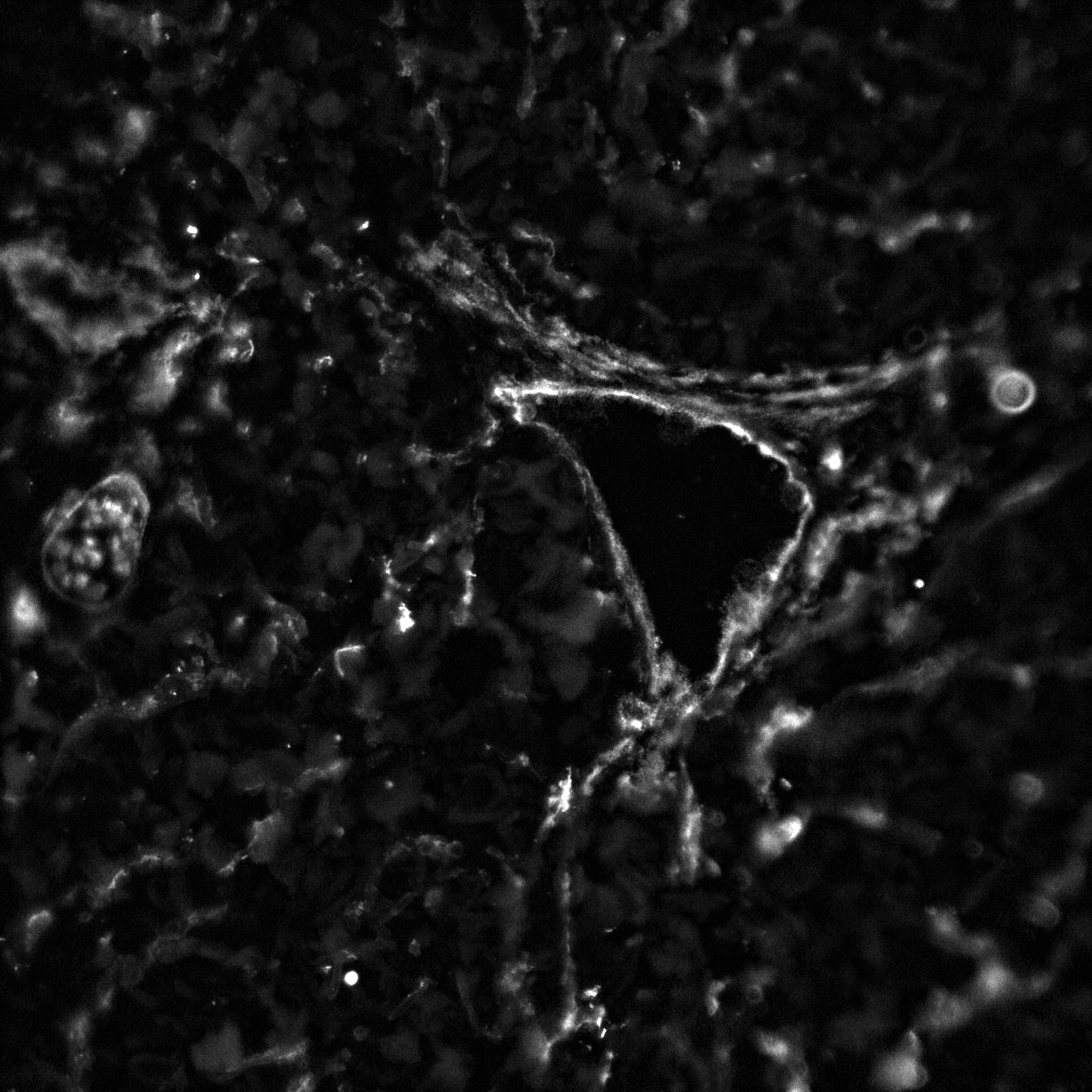

Supplement: Supplementary file 3 — Source data Fig. 1 [file 44321_2025_249_MOESM3_ESM.zip › Figure 1/P-AKT_Panel_3.tif]

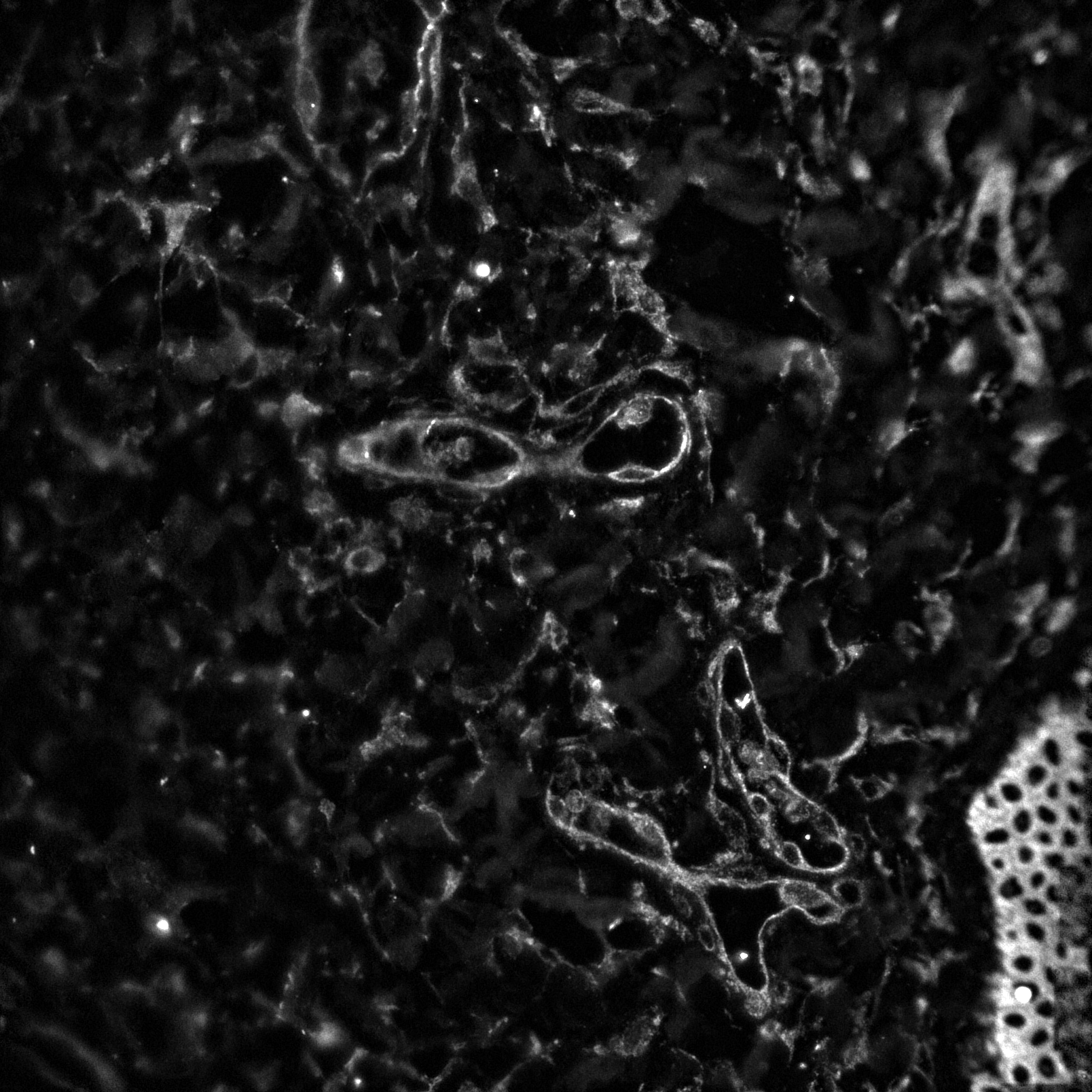

Supplement: Supplementary file 3 — Source data Fig. 1 [file 44321_2025_249_MOESM3_ESM.zip › Figure 1/P-AKT_Panel_4.tif]

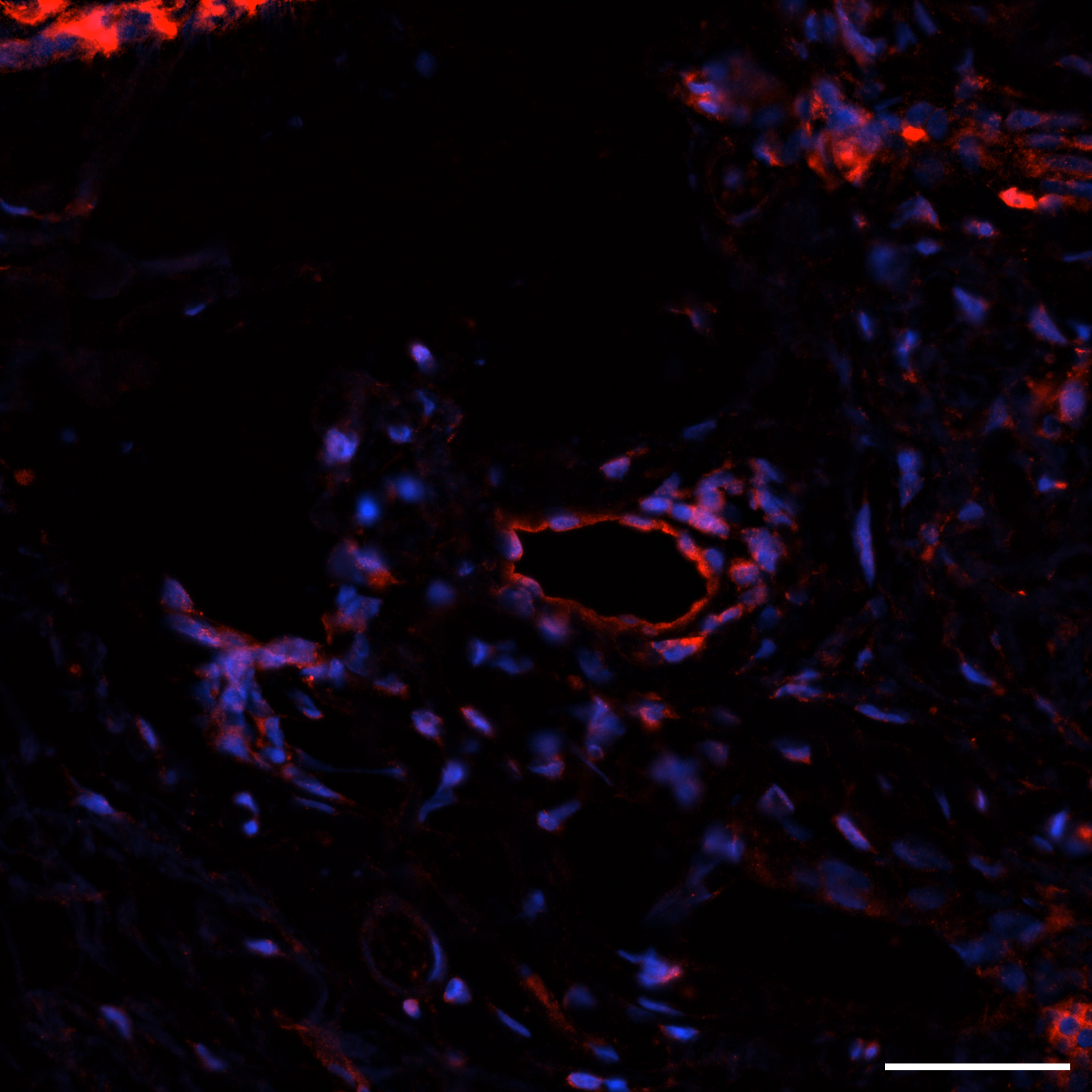

Supplement: Supplementary file 3 — Source data Fig. 1 [file 44321_2025_249_MOESM3_ESM.zip › Figure 1/P-S6RP_Panel_1.tif]

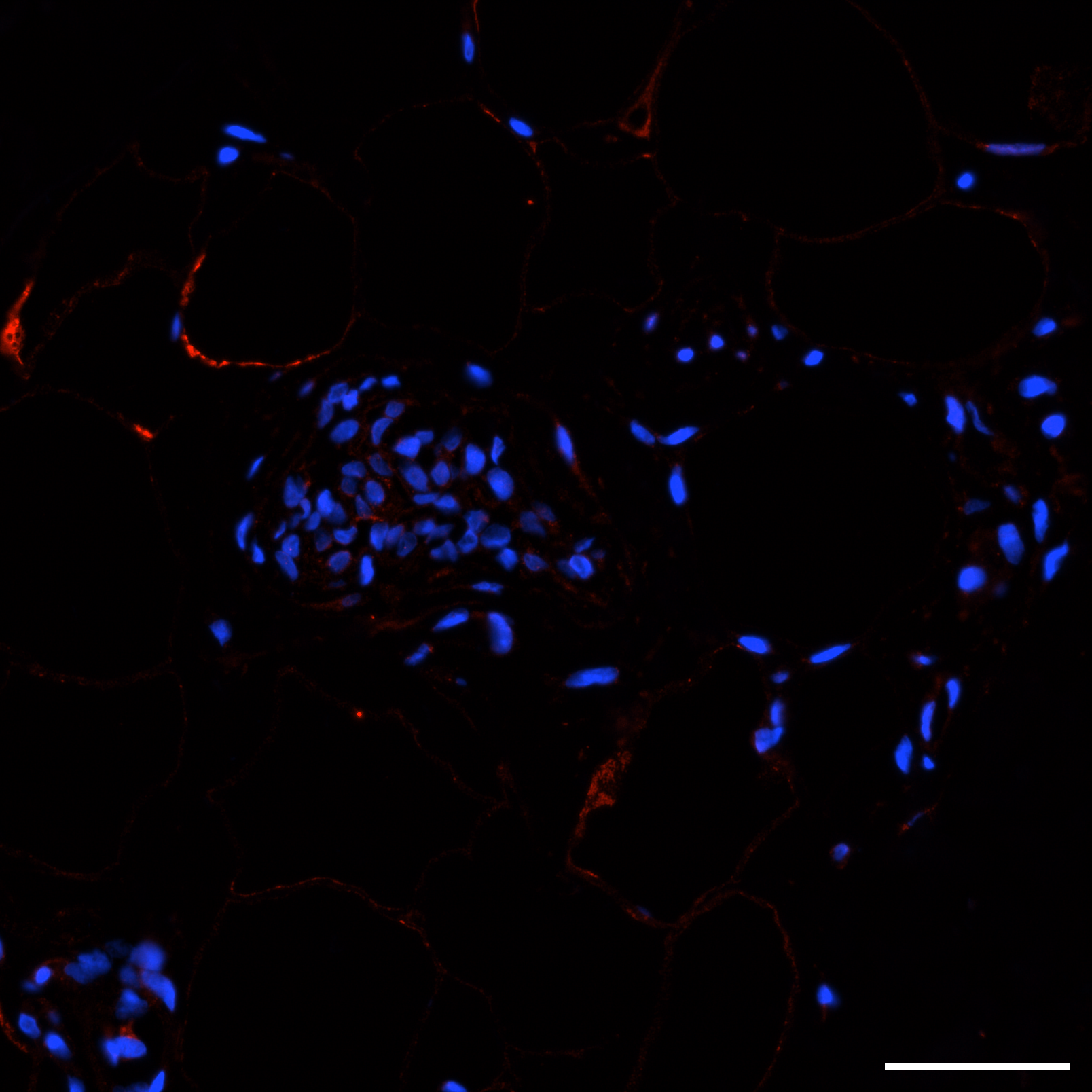

Supplement: Supplementary file 3 — Source data Fig. 1 [file 44321_2025_249_MOESM3_ESM.zip › Figure 1/P-S6RP_Panel_2.tif]

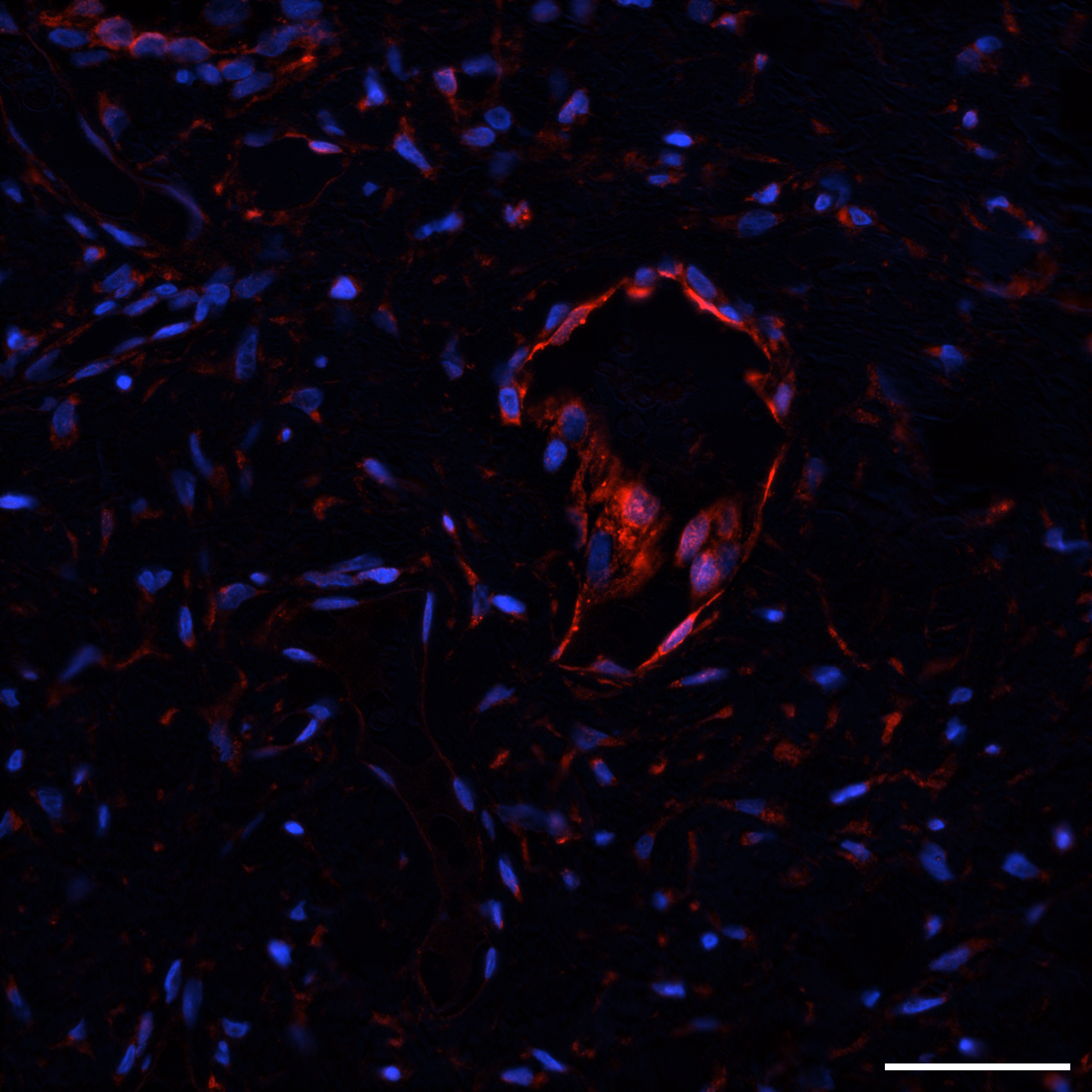

Supplement: Supplementary file 3 — Source data Fig. 1 [file 44321_2025_249_MOESM3_ESM.zip › Figure 1/P-S6RP_Panel_3.tif]

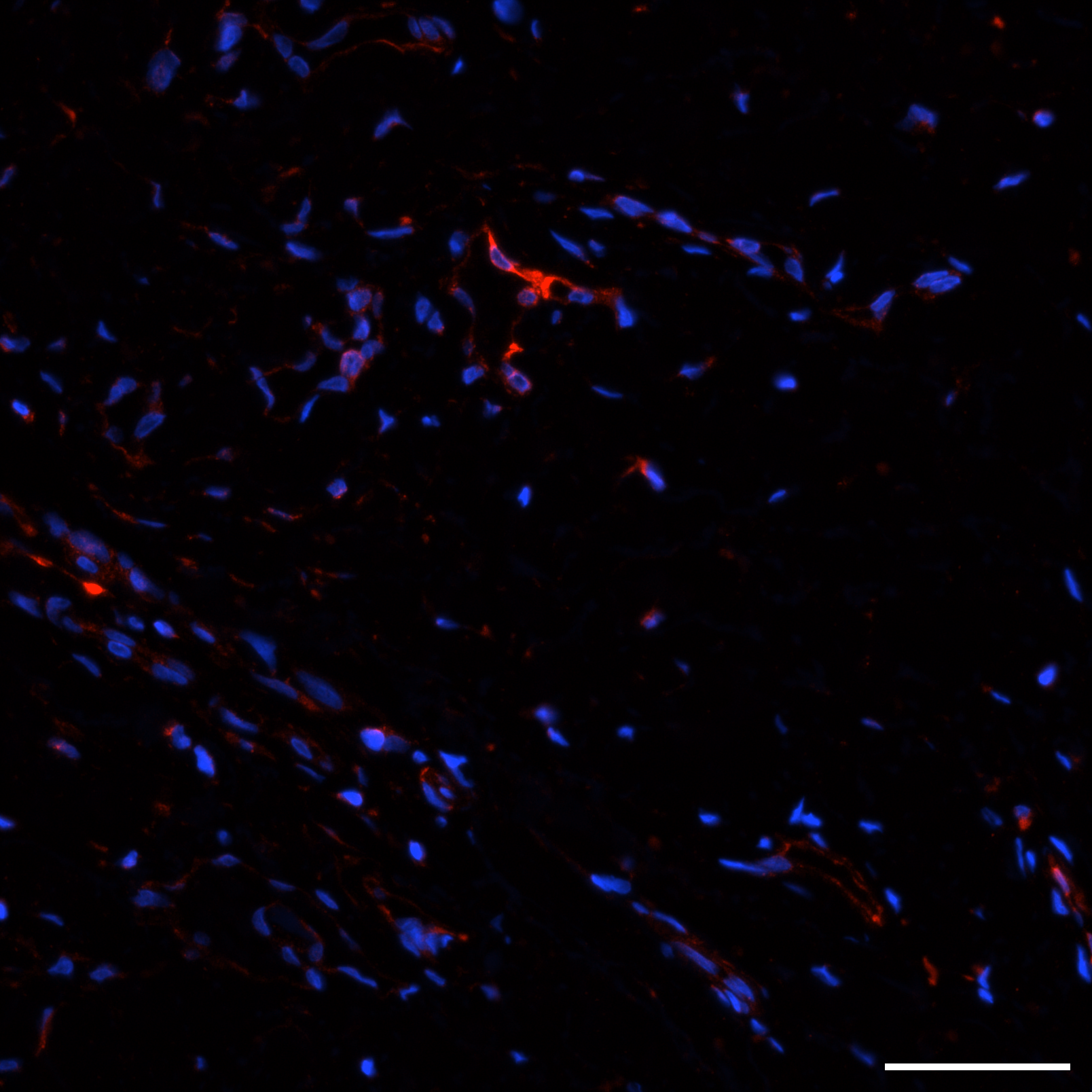

Supplement: Supplementary file 3 — Source data Fig. 1 [file 44321_2025_249_MOESM3_ESM.zip › Figure 1/P-S6RP_Panel_4.tif]

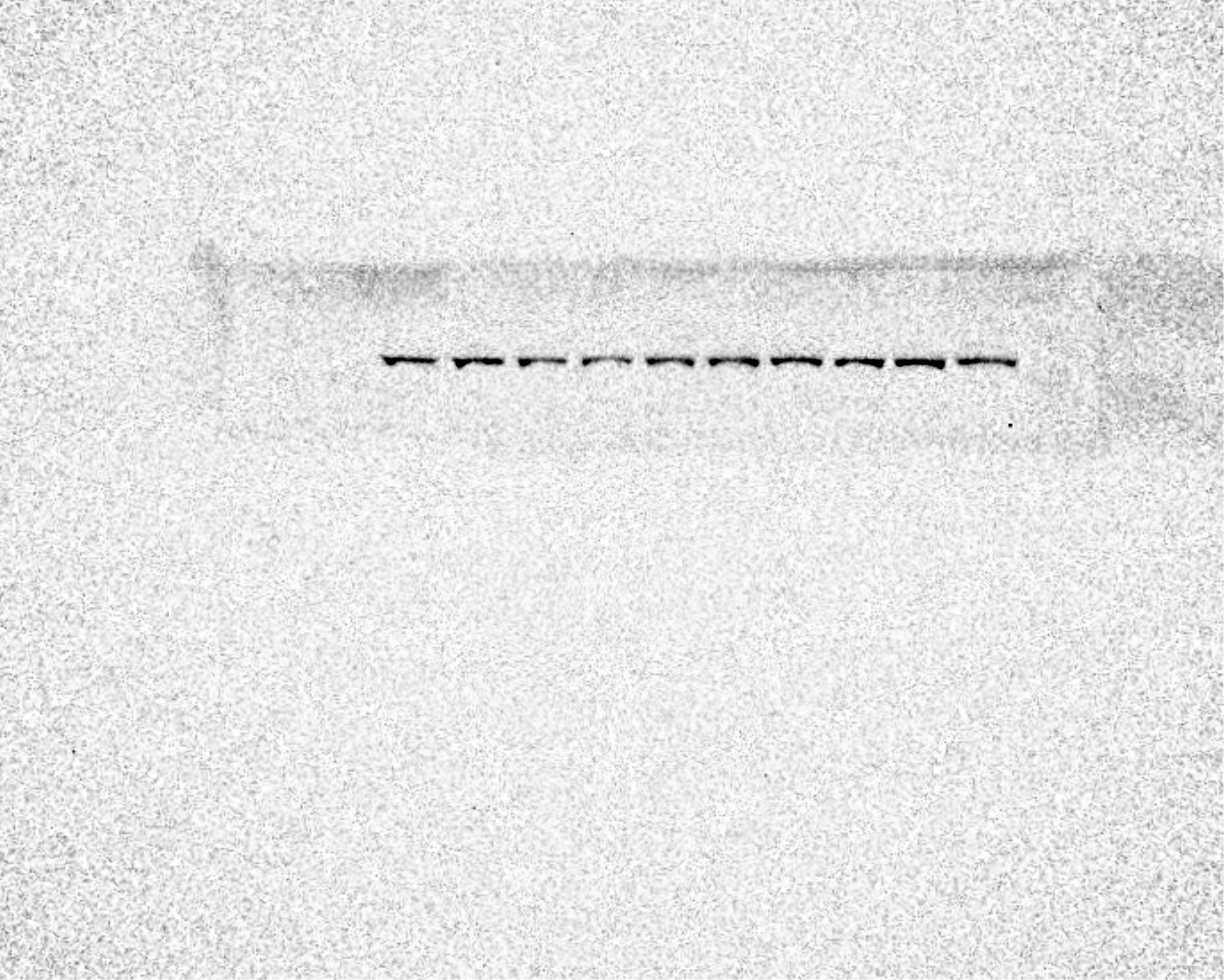

Supplement: Supplementary file 4 — Source data Fig. 2 [file 44321_2025_249_MOESM4_ESM.zip › Figure 2/2A_blot_p110a.tif]

Figure 2A – Blot – p110α

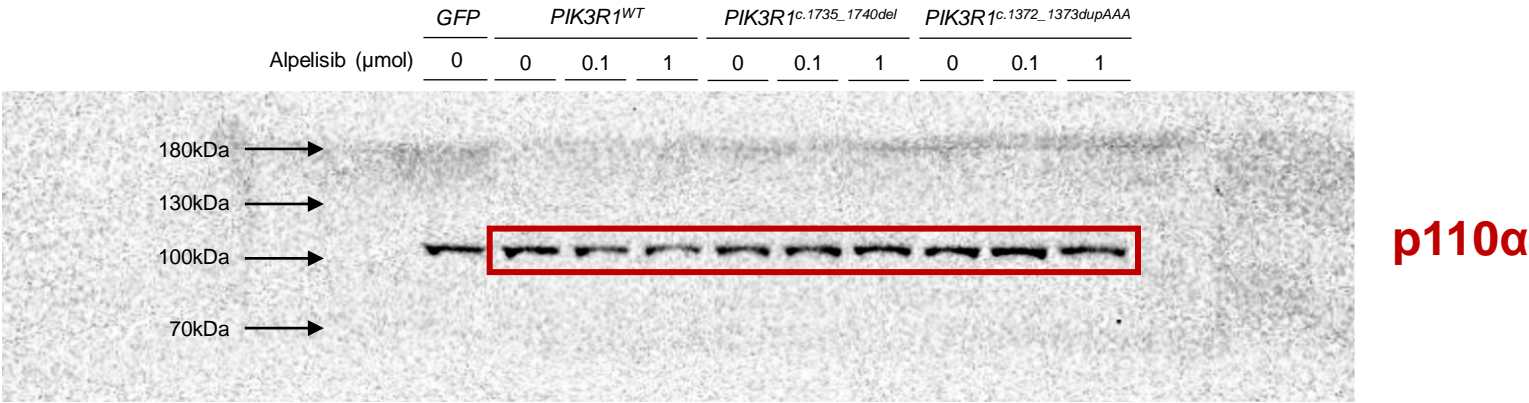

Figure 2A – Blot –  $\alpha$ -tubulin

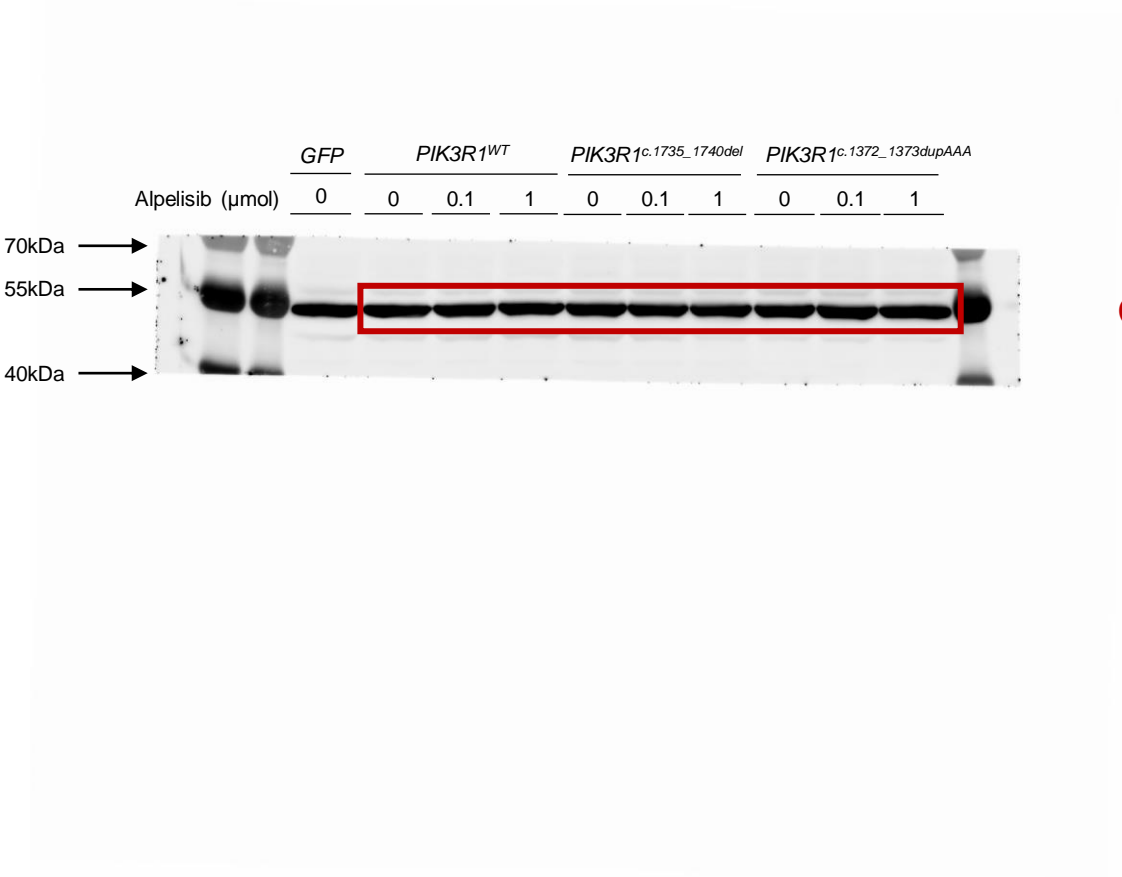

$\alpha$ -tubulin

Supplement: Supplementary file 4 — Source data Fig. 2 [file 44321_2025_249_MOESM4_ESM.zip › Figure 2/2A_blot_summary.pdf]

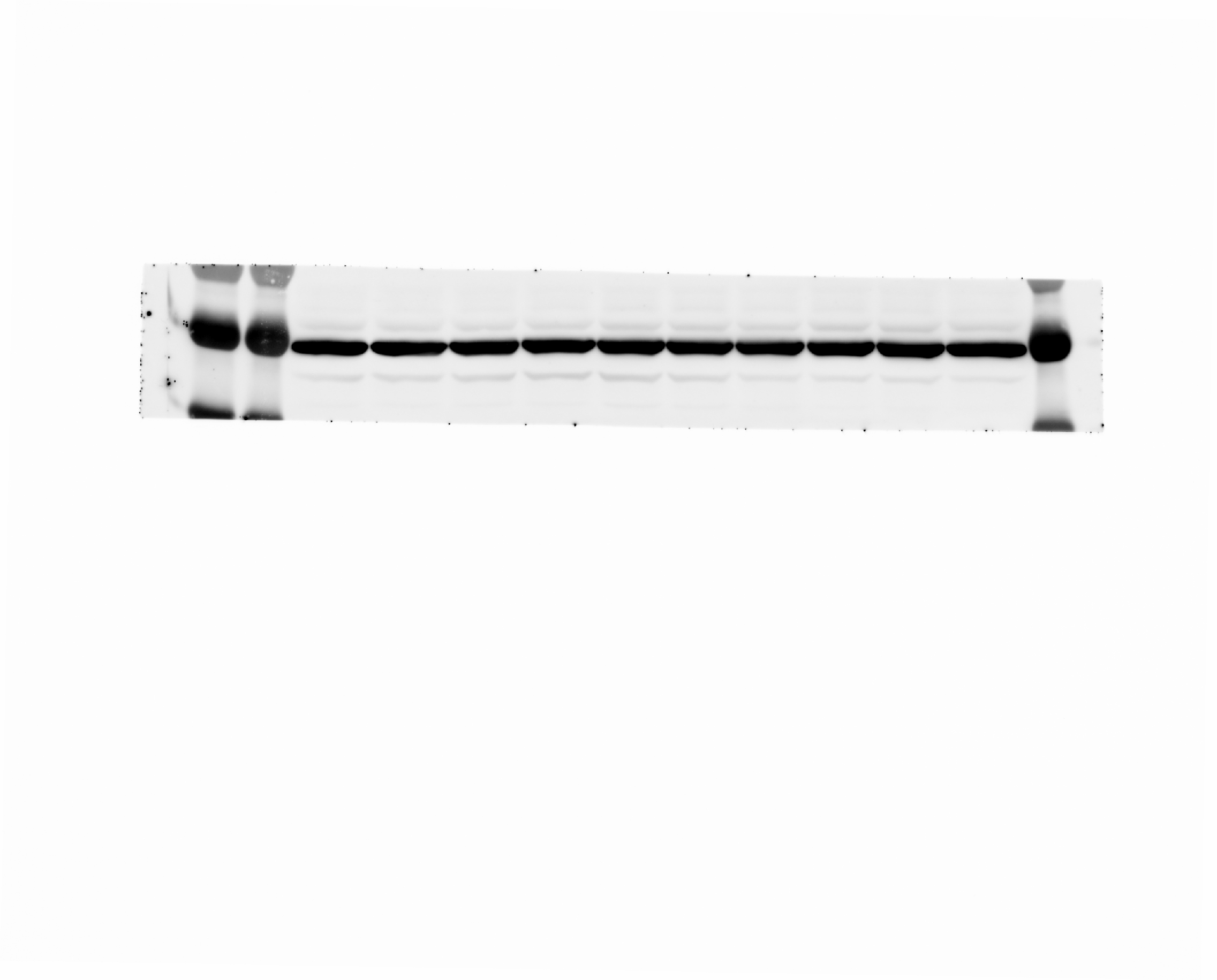

Supplement: Supplementary file 4 — Source data Fig. 2 [file 44321_2025_249_MOESM4_ESM.zip › Figure 2/2A_blot_tubulin.tif]

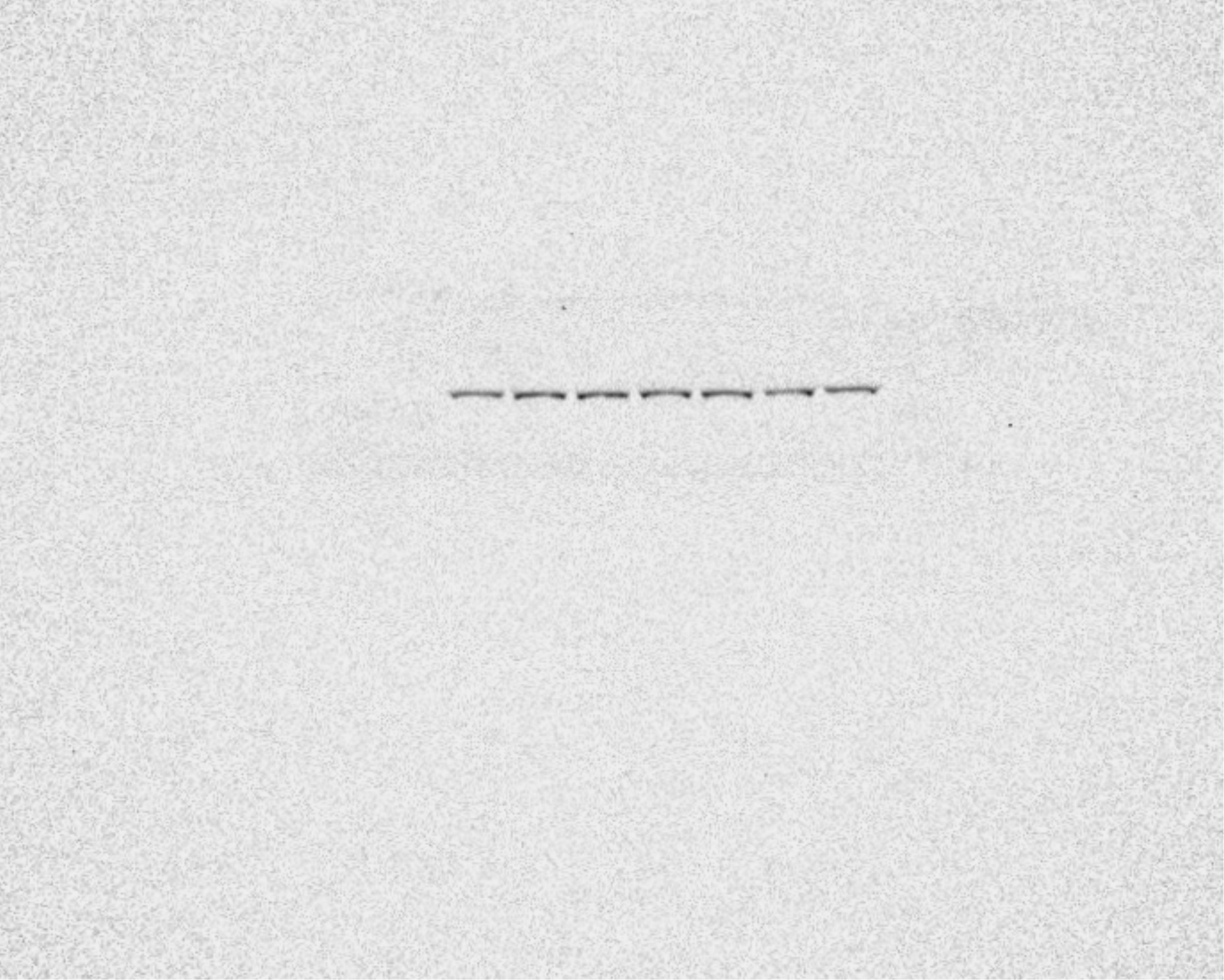

Supplement: Supplementary file 4 — Source data Fig. 2 [file 44321_2025_249_MOESM4_ESM.zip › Figure 2/2B_blot_p110.tif]

Figure 2B – Blot – p110α

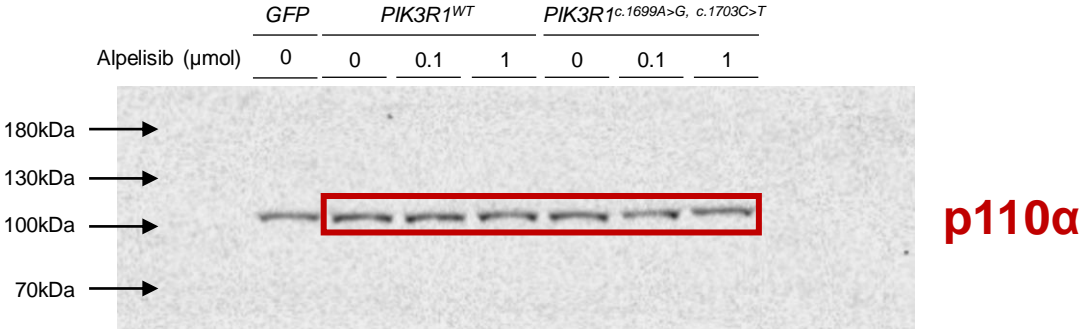

Figure 2B – Blot –  $\alpha$ -tubulin

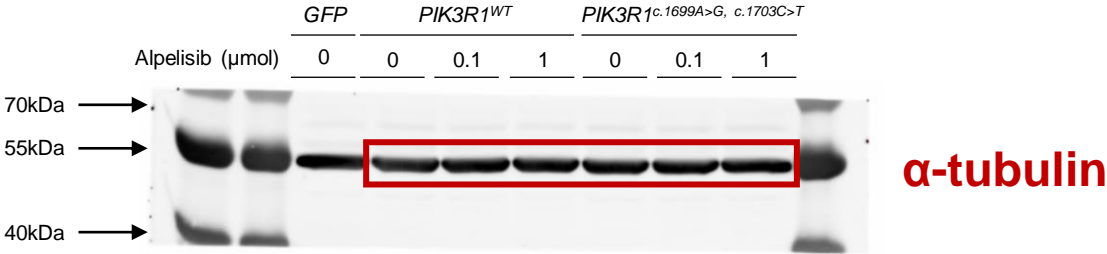

Supplement: Supplementary file 4 — Source data Fig. 2 [file 44321_2025_249_MOESM4_ESM.zip › Figure 2/2B_blot_summary.pdf]

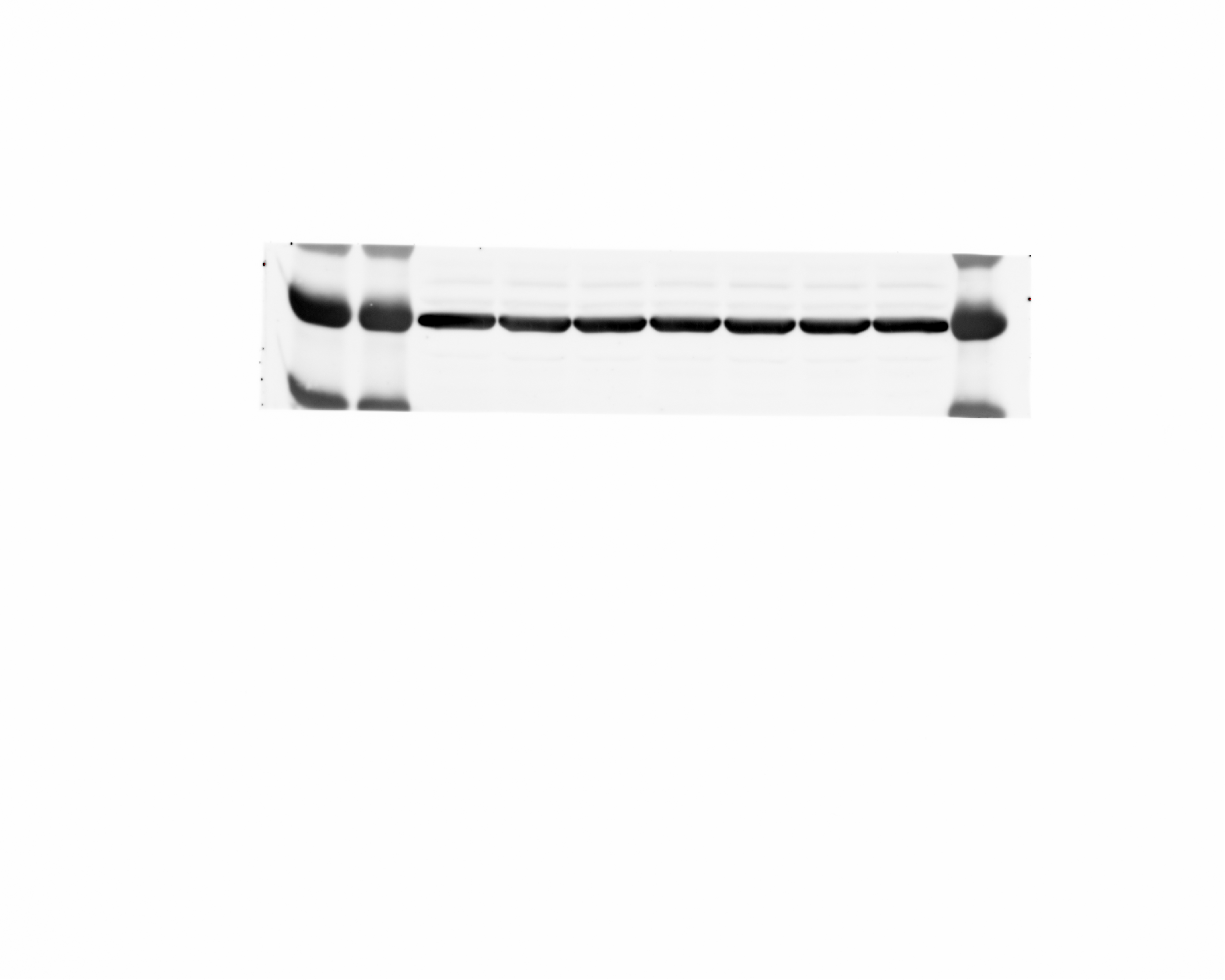

Supplement: Supplementary file 4 — Source data Fig. 2 [file 44321_2025_249_MOESM4_ESM.zip › Figure 2/2B_blot_tubulin.tif]

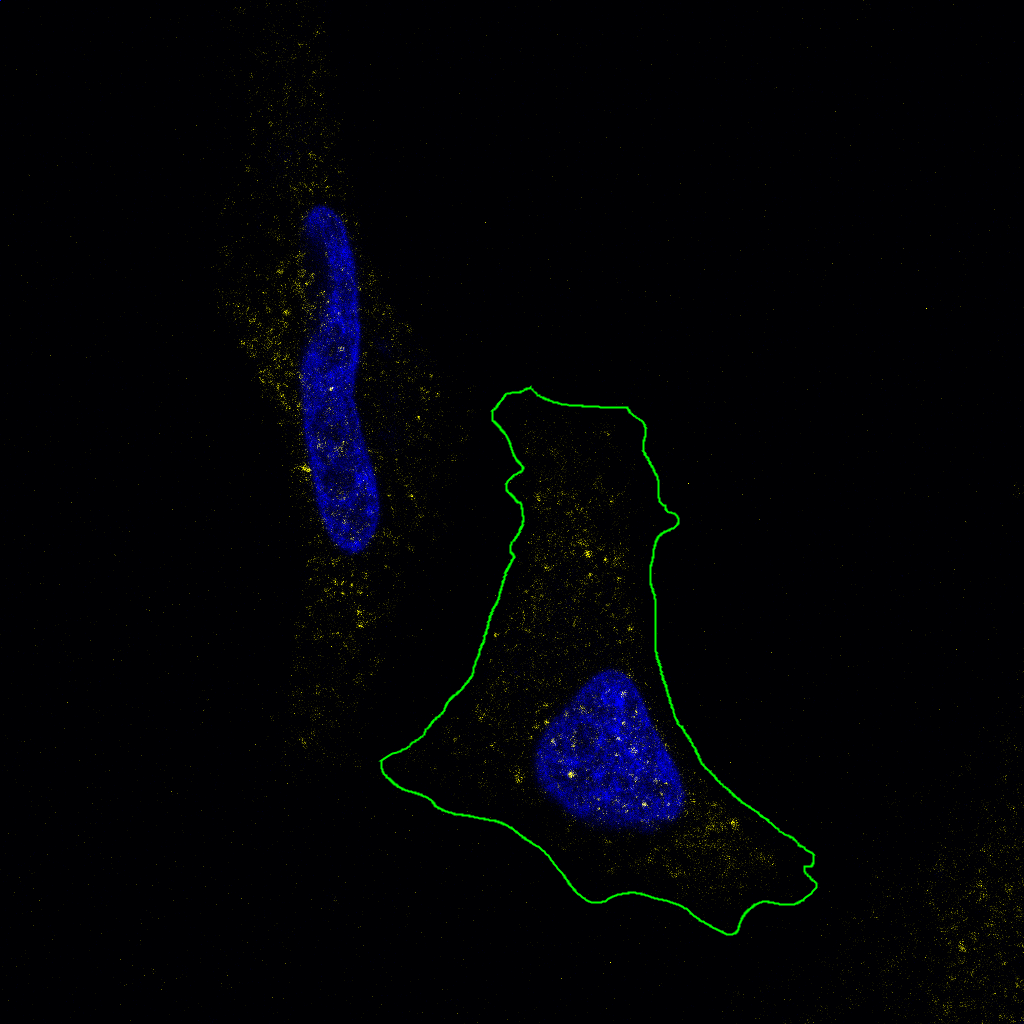

Supplement: Supplementary file 4 — Source data Fig. 2 [file 44321_2025_249_MOESM4_ESM.zip › Figure 2/2C_Row_1_Panel_1.tif]

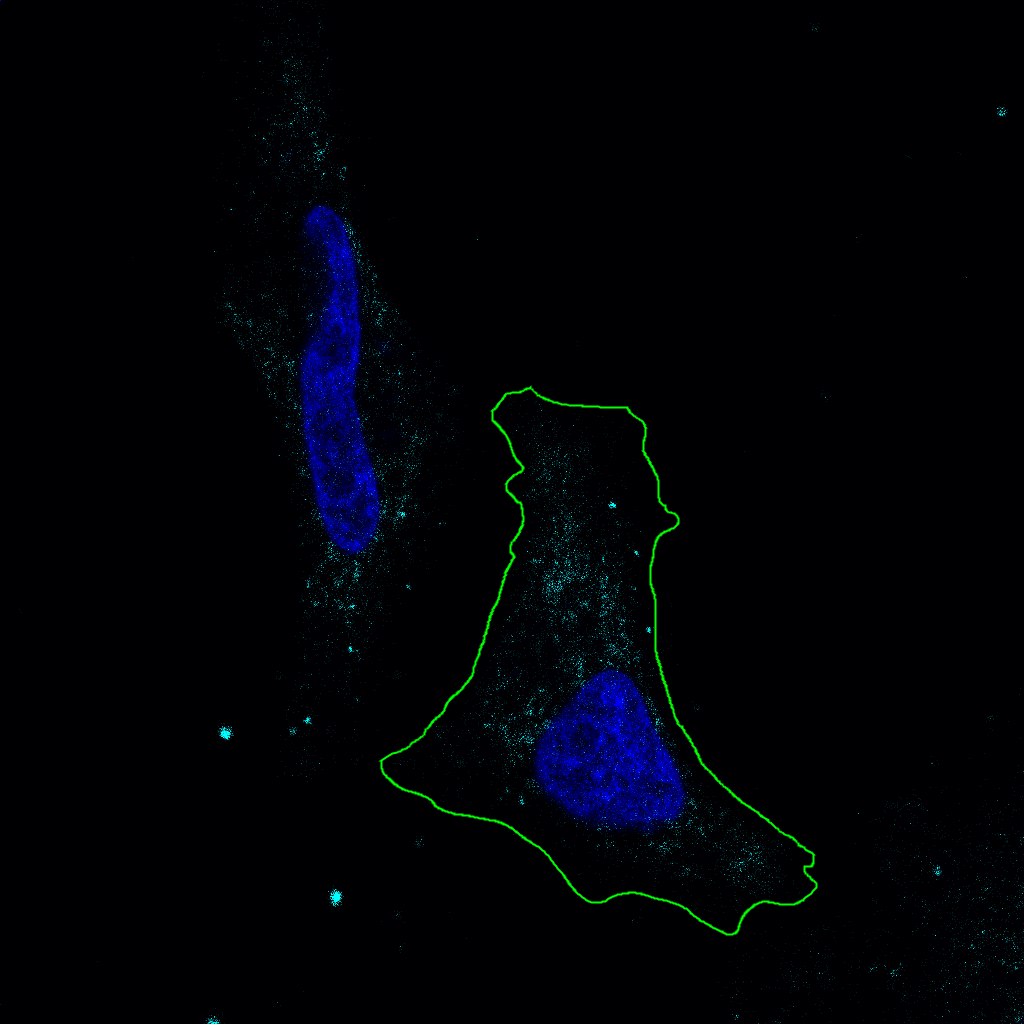

Supplement: Supplementary file 4 — Source data Fig. 2 [file 44321_2025_249_MOESM4_ESM.zip › Figure 2/2C_Row_1_Panel_2.tif]

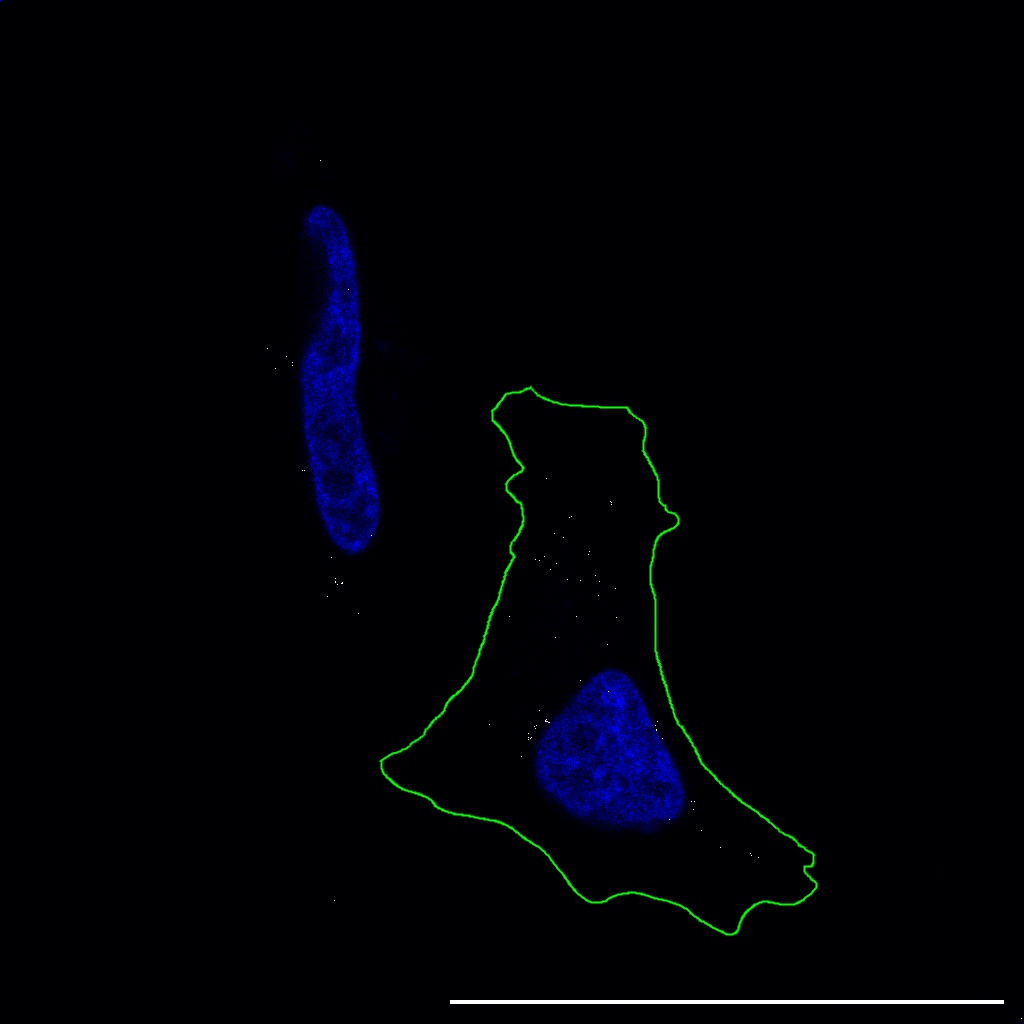

Supplement: Supplementary file 4 — Source data Fig. 2 [file 44321_2025_249_MOESM4_ESM.zip › Figure 2/2C_Row_1_Panel_3.tif]

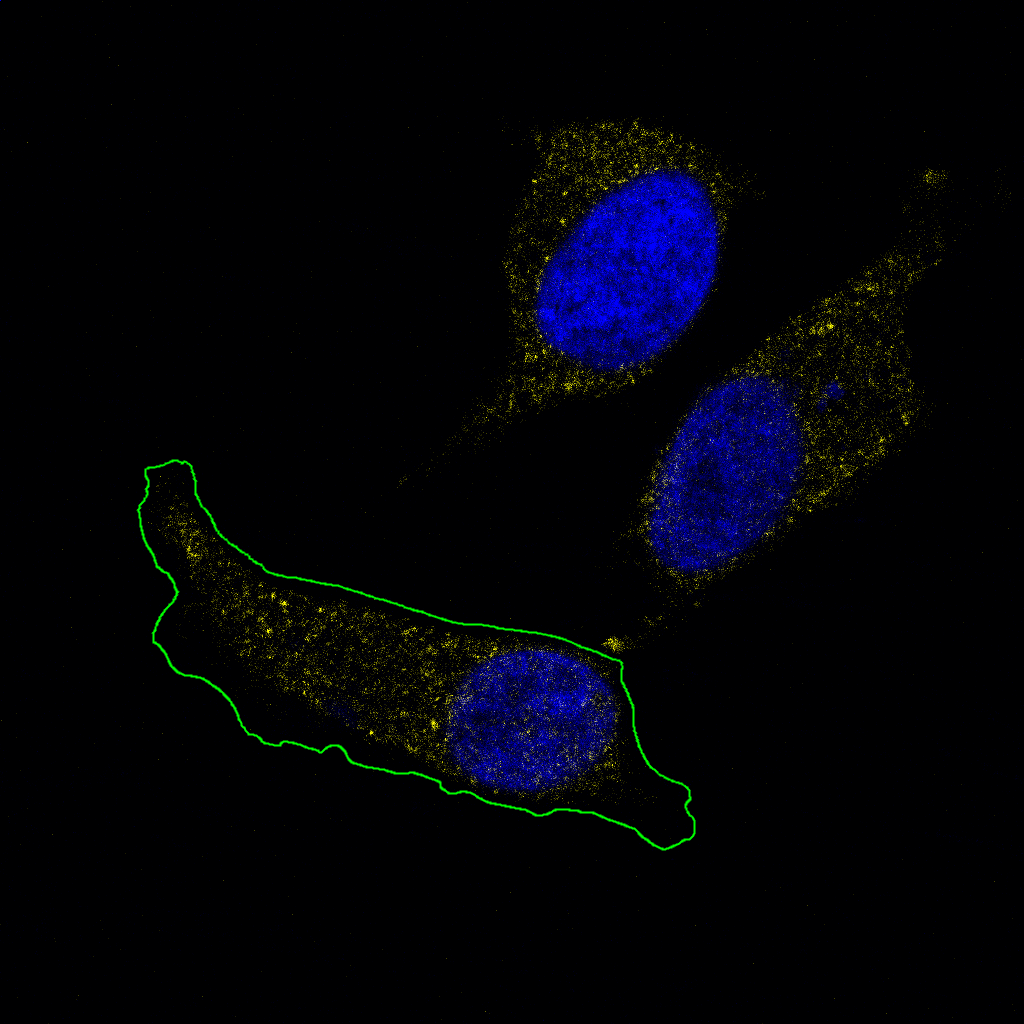

Supplement: Supplementary file 4 — Source data Fig. 2 [file 44321_2025_249_MOESM4_ESM.zip › Figure 2/2C_Row_2_Panel_1.tif]

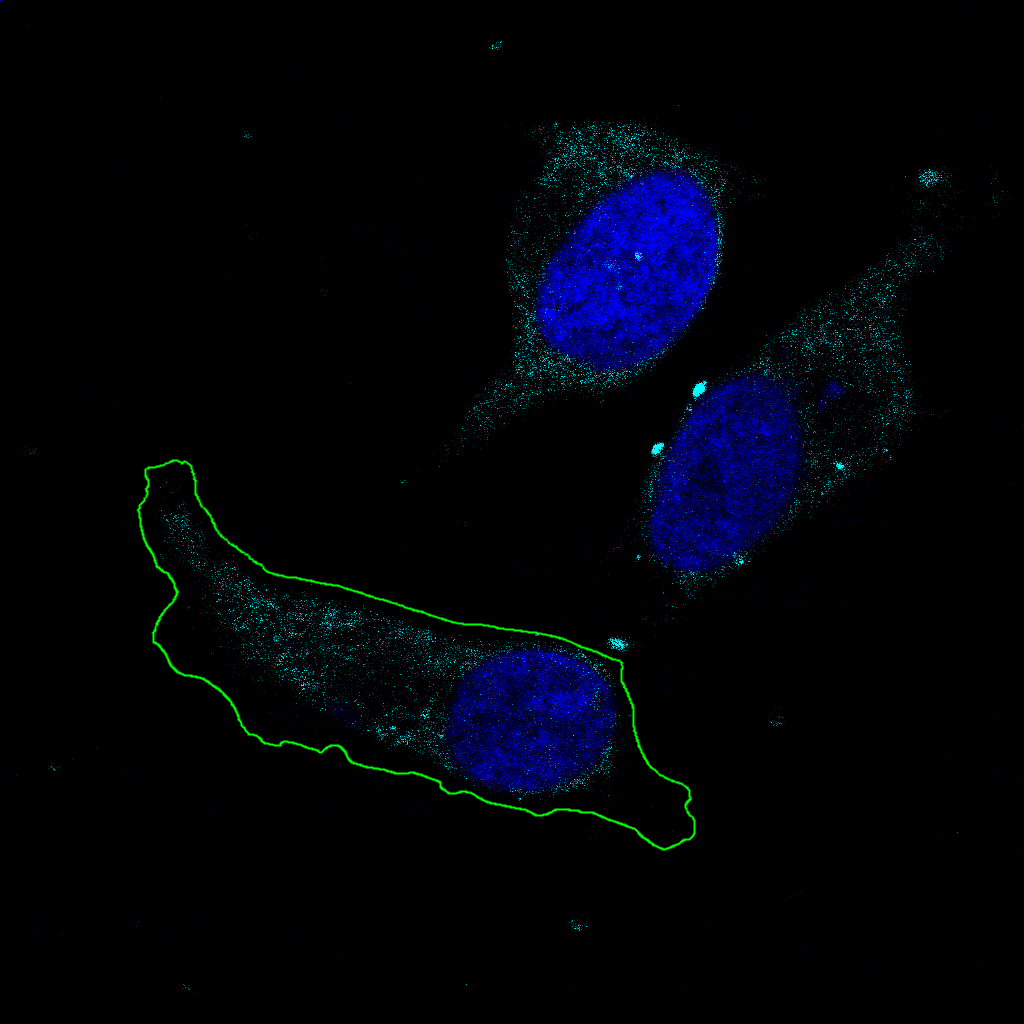

Supplement: Supplementary file 4 — Source data Fig. 2 [file 44321_2025_249_MOESM4_ESM.zip › Figure 2/2C_Row_2_Panel_2.tif]

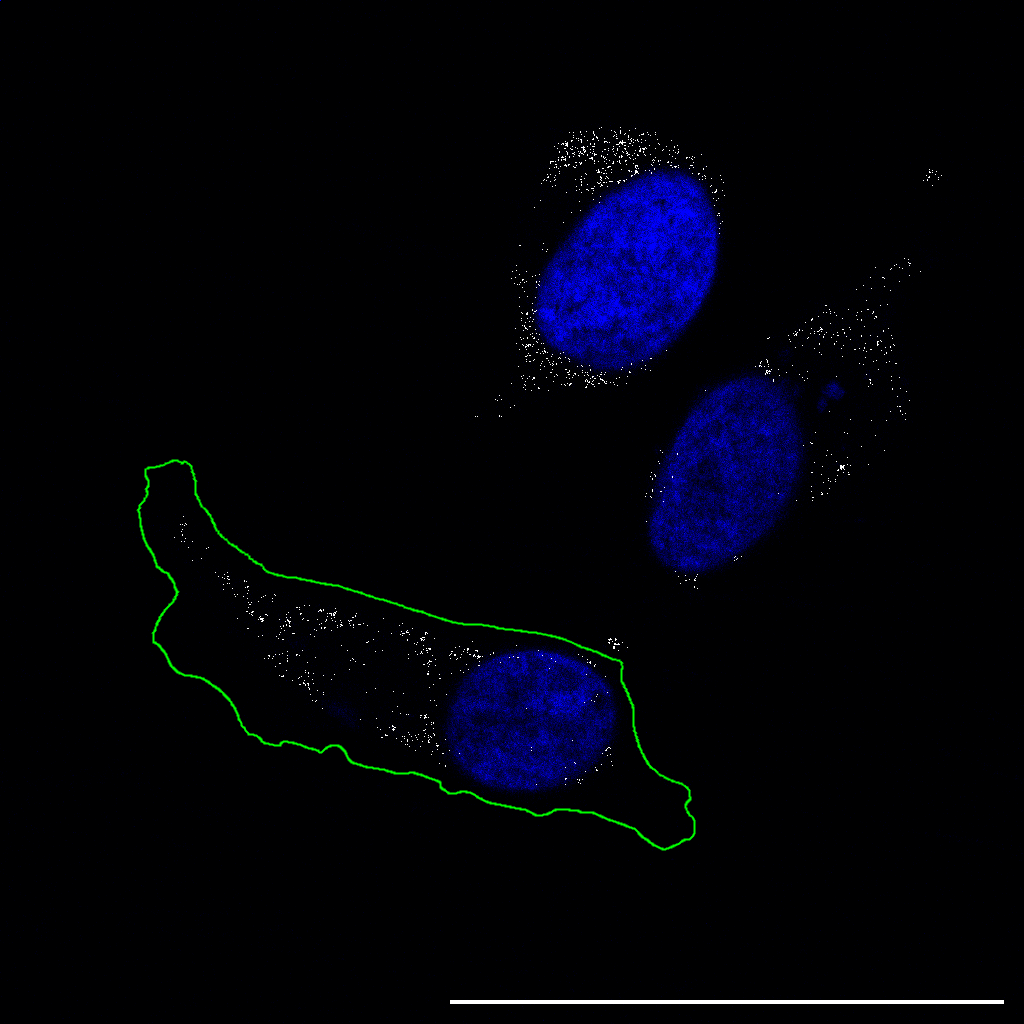

Supplement: Supplementary file 4 — Source data Fig. 2 [file 44321_2025_249_MOESM4_ESM.zip › Figure 2/2C_Row_2_Panel_3.tif]

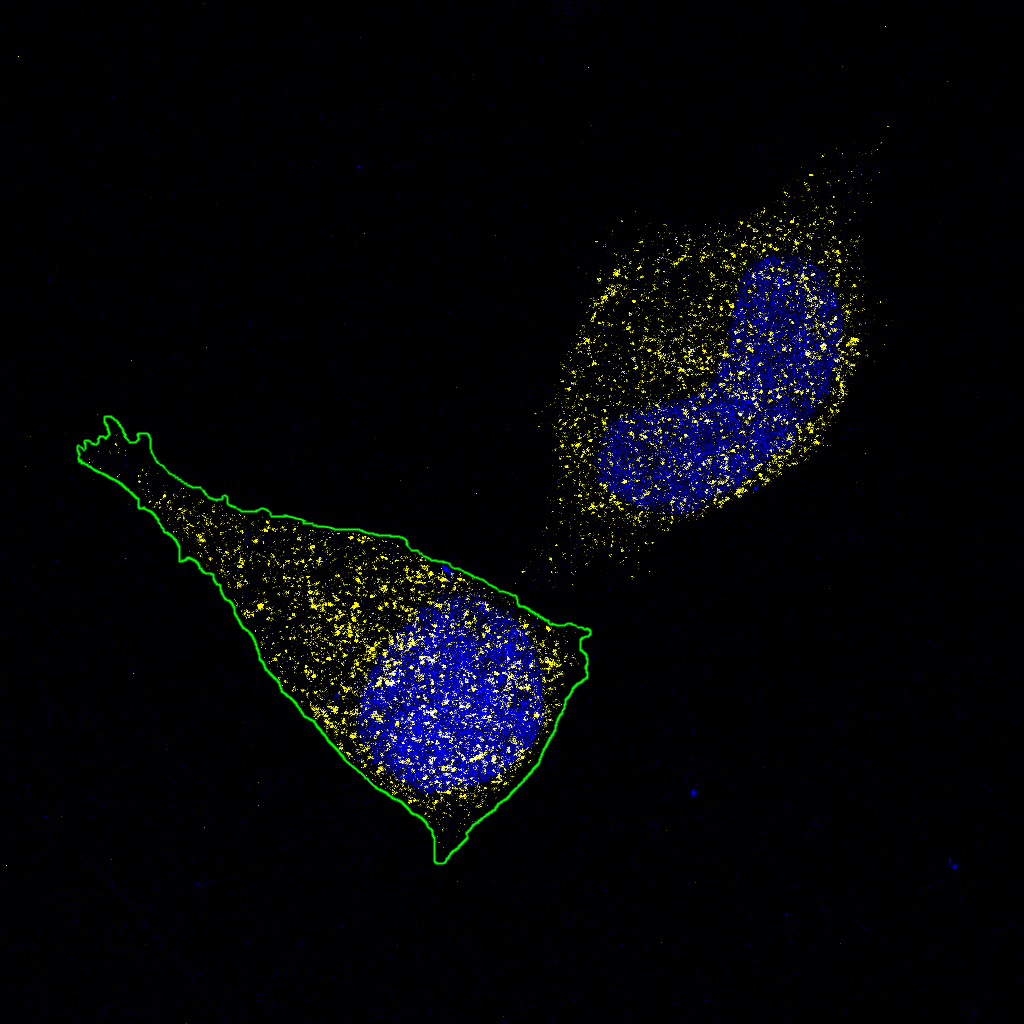

Supplement: Supplementary file 4 — Source data Fig. 2 [file 44321_2025_249_MOESM4_ESM.zip › Figure 2/2C_Row_3_Panel_1.tif]

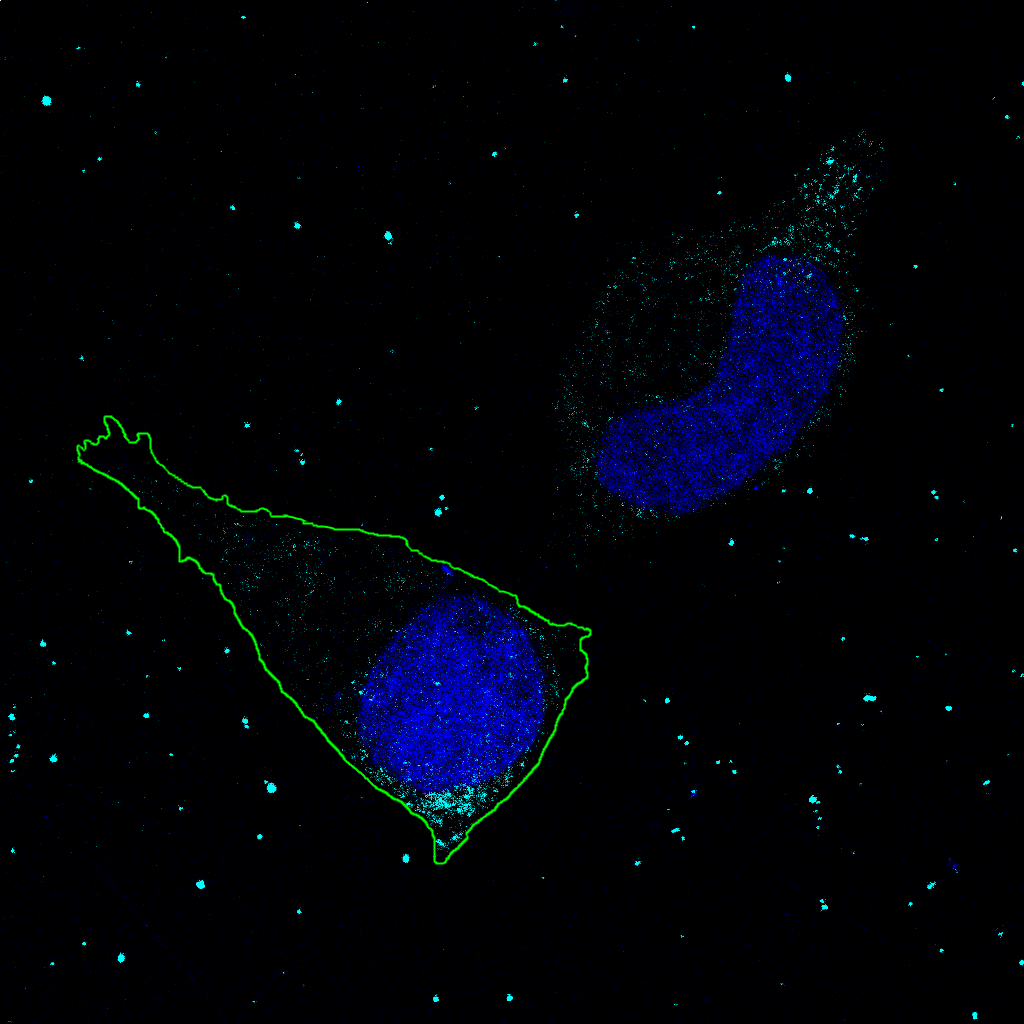

Supplement: Supplementary file 4 — Source data Fig. 2 [file 44321_2025_249_MOESM4_ESM.zip › Figure 2/2C_Row_3_Panel_2.tif]

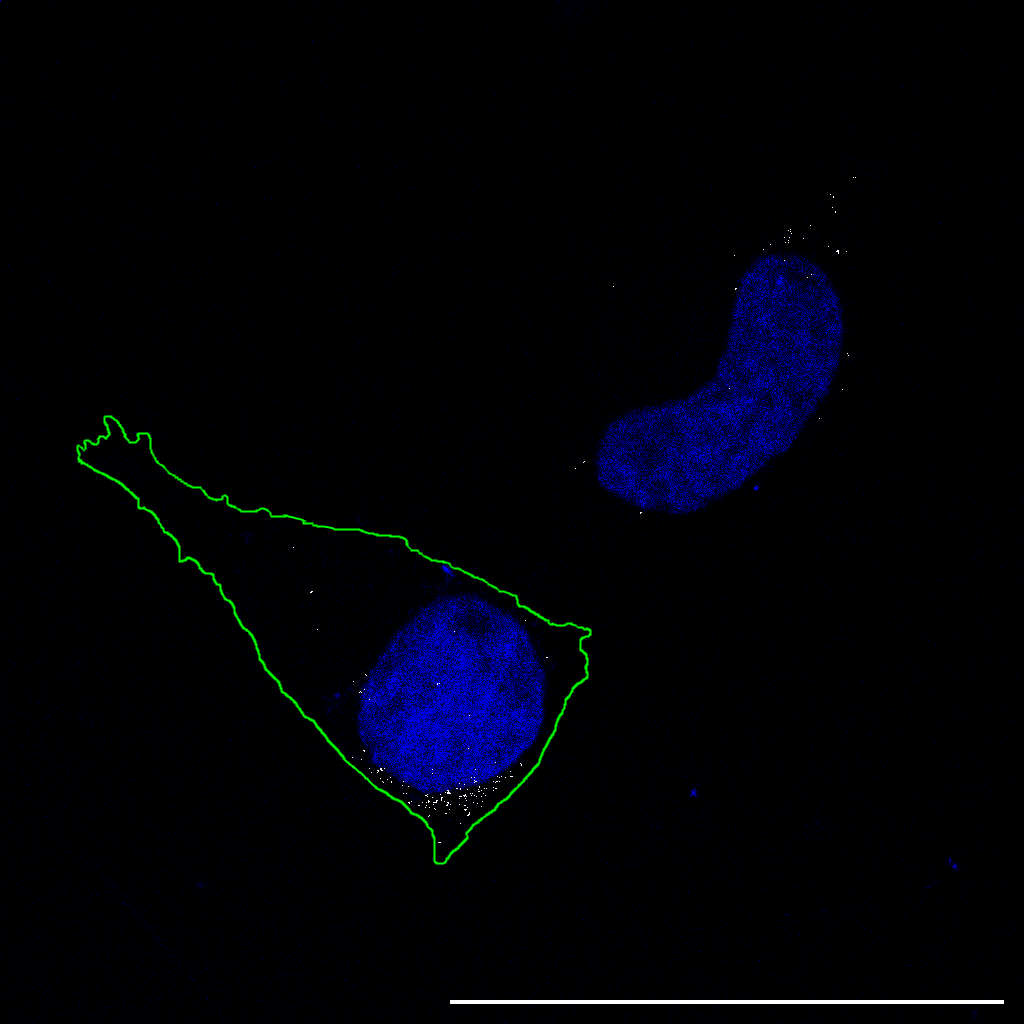

Supplement: Supplementary file 4 — Source data Fig. 2 [file 44321_2025_249_MOESM4_ESM.zip › Figure 2/2C_Row_3_Panel_3.tif]

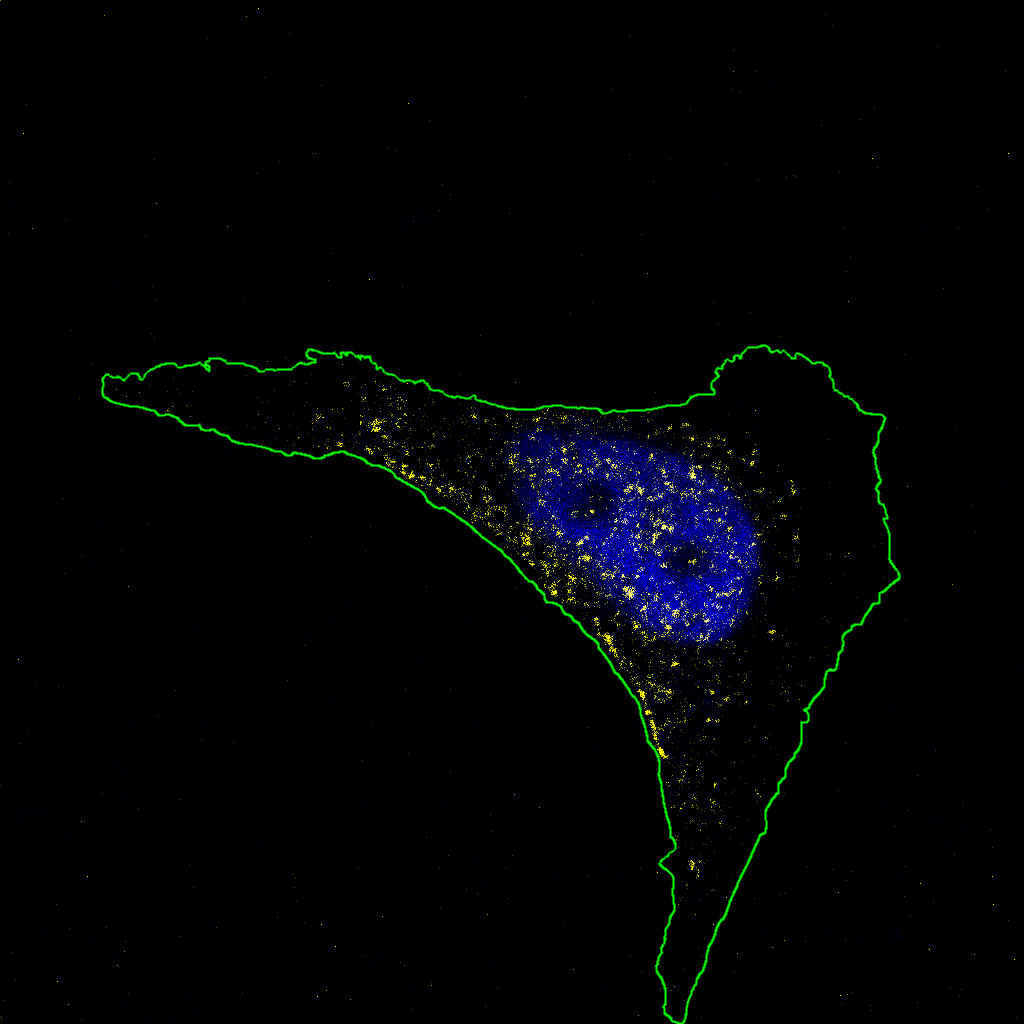

Supplement: Supplementary file 4 — Source data Fig. 2 [file 44321_2025_249_MOESM4_ESM.zip › Figure 2/2C_Row_4_Panel_1.tif]

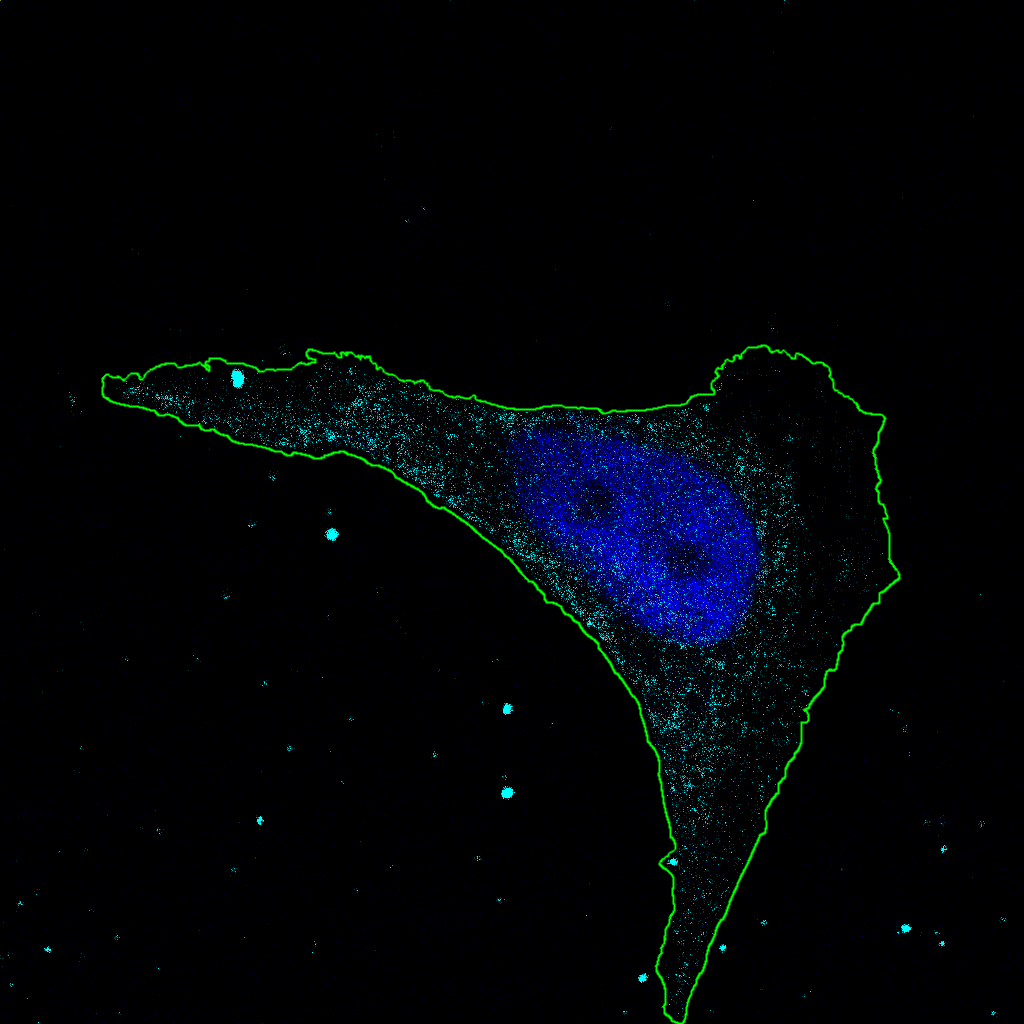

Supplement: Supplementary file 4 — Source data Fig. 2 [file 44321_2025_249_MOESM4_ESM.zip › Figure 2/2C_Row_4_Panel_2.tif]

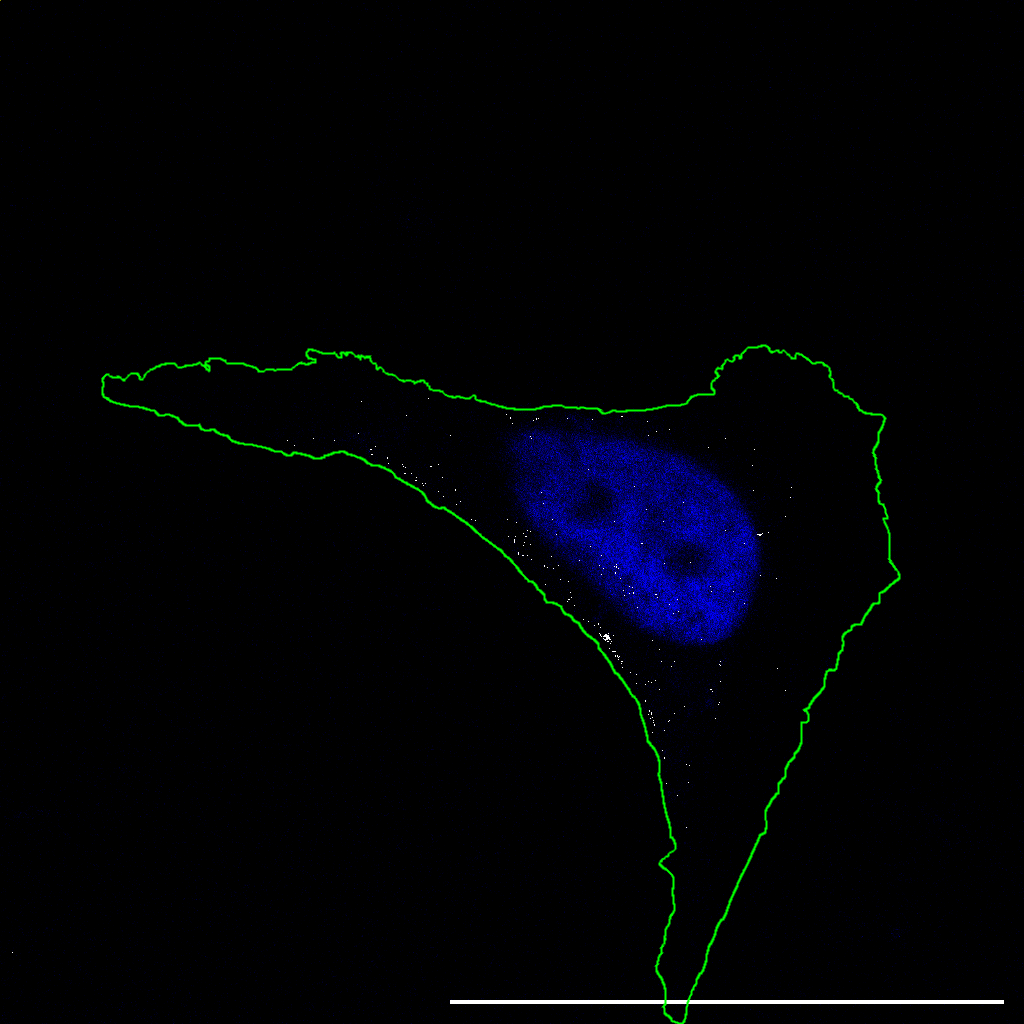

Supplement: Supplementary file 4 — Source data Fig. 2 [file 44321_2025_249_MOESM4_ESM.zip › Figure 2/2C_Row_4_Panel_3.tif]

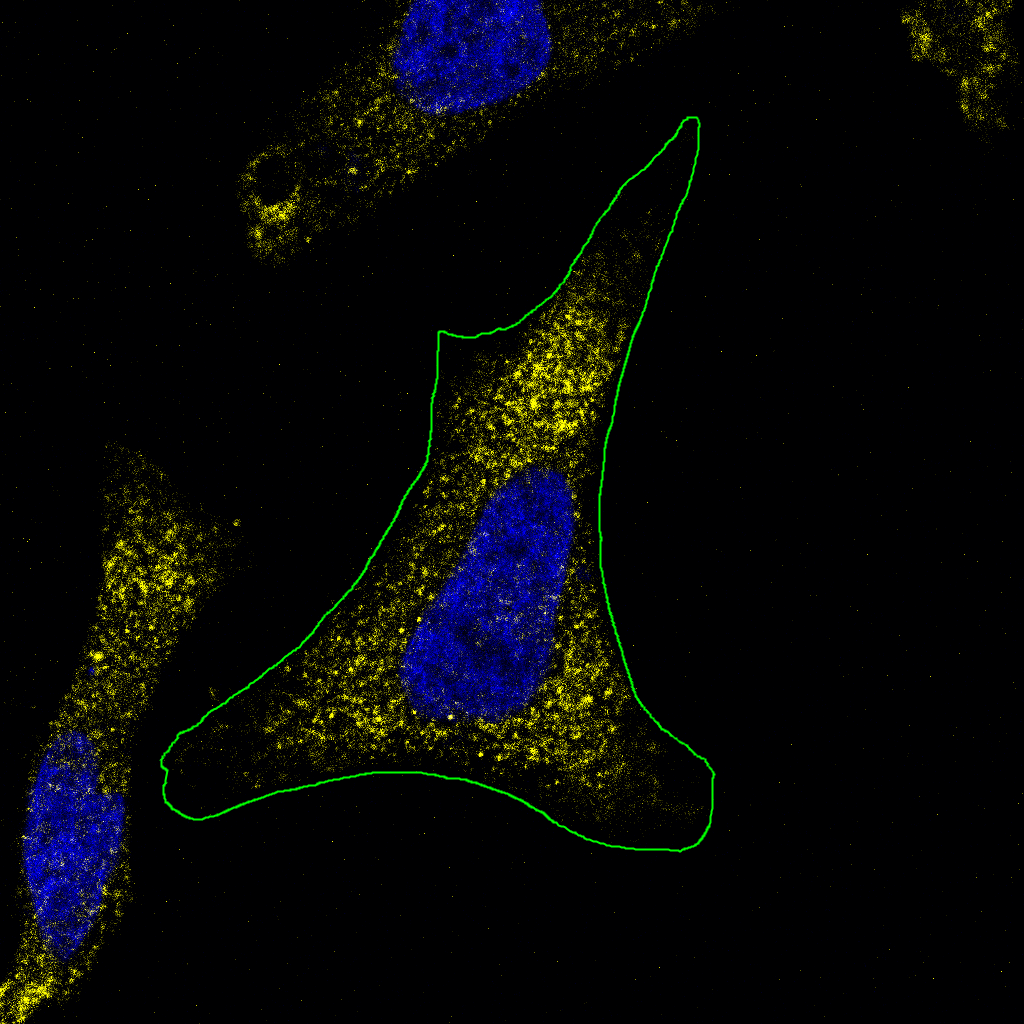

Supplement: Supplementary file 4 — Source data Fig. 2 [file 44321_2025_249_MOESM4_ESM.zip › Figure 2/2C_Row_5_Panel_1.tif]

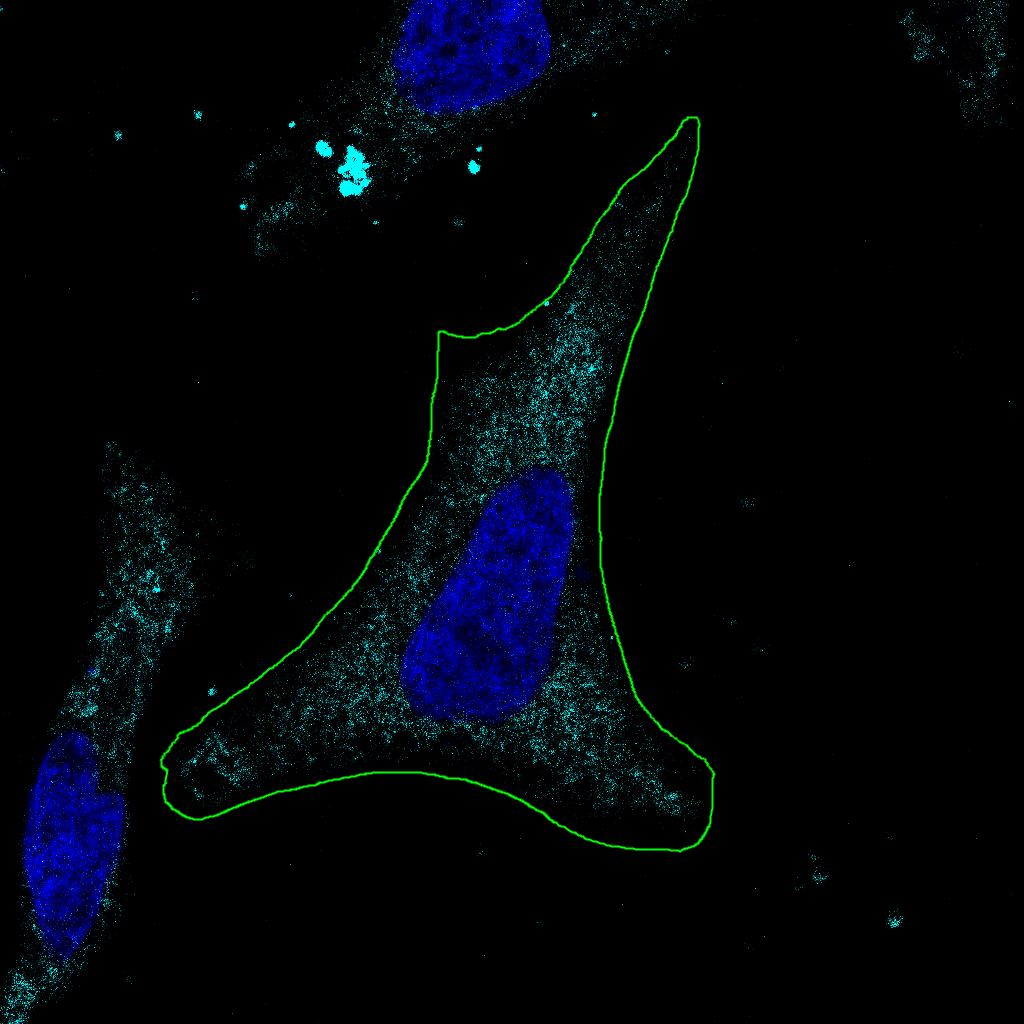

Supplement: Supplementary file 4 — Source data Fig. 2 [file 44321_2025_249_MOESM4_ESM.zip › Figure 2/2C_Row_5_Panel_2.tif]

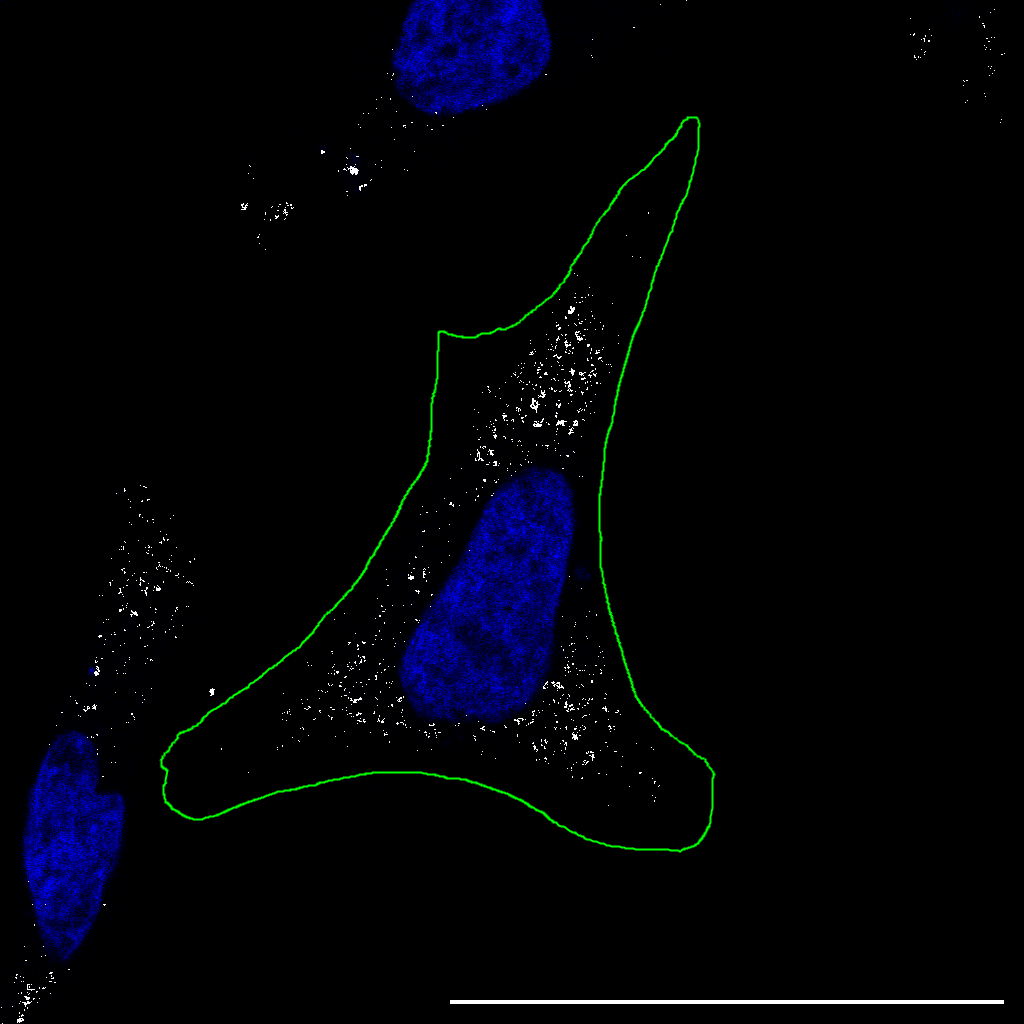

Supplement: Supplementary file 4 — Source data Fig. 2 [file 44321_2025_249_MOESM4_ESM.zip › Figure 2/2C_Row_5_Panel_3.tif]

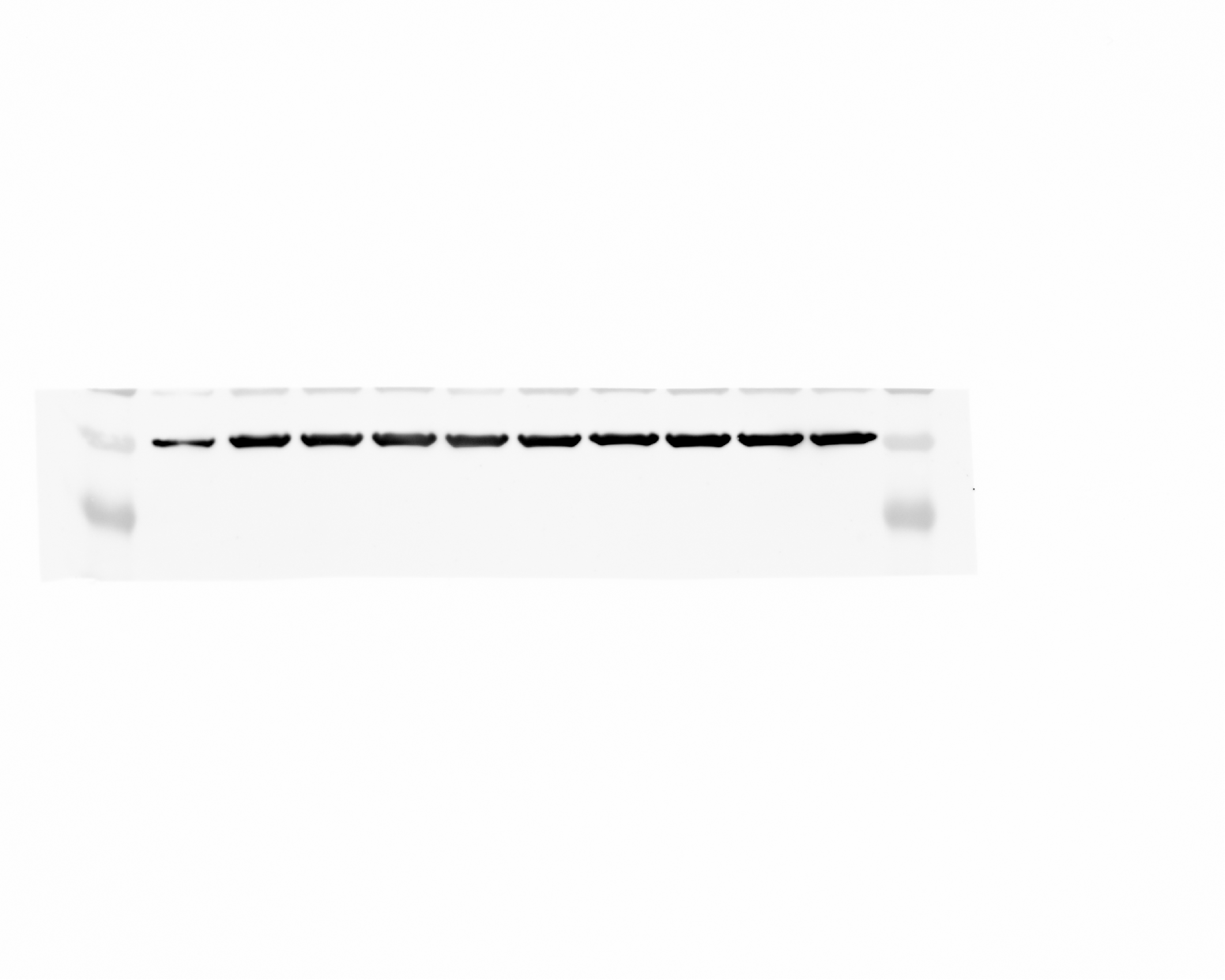

Supplement: Supplementary file 5 — Source data Fig. 3 [file 44321_2025_249_MOESM5_ESM.zip › Figure 3/3A_blot_actin.tif]

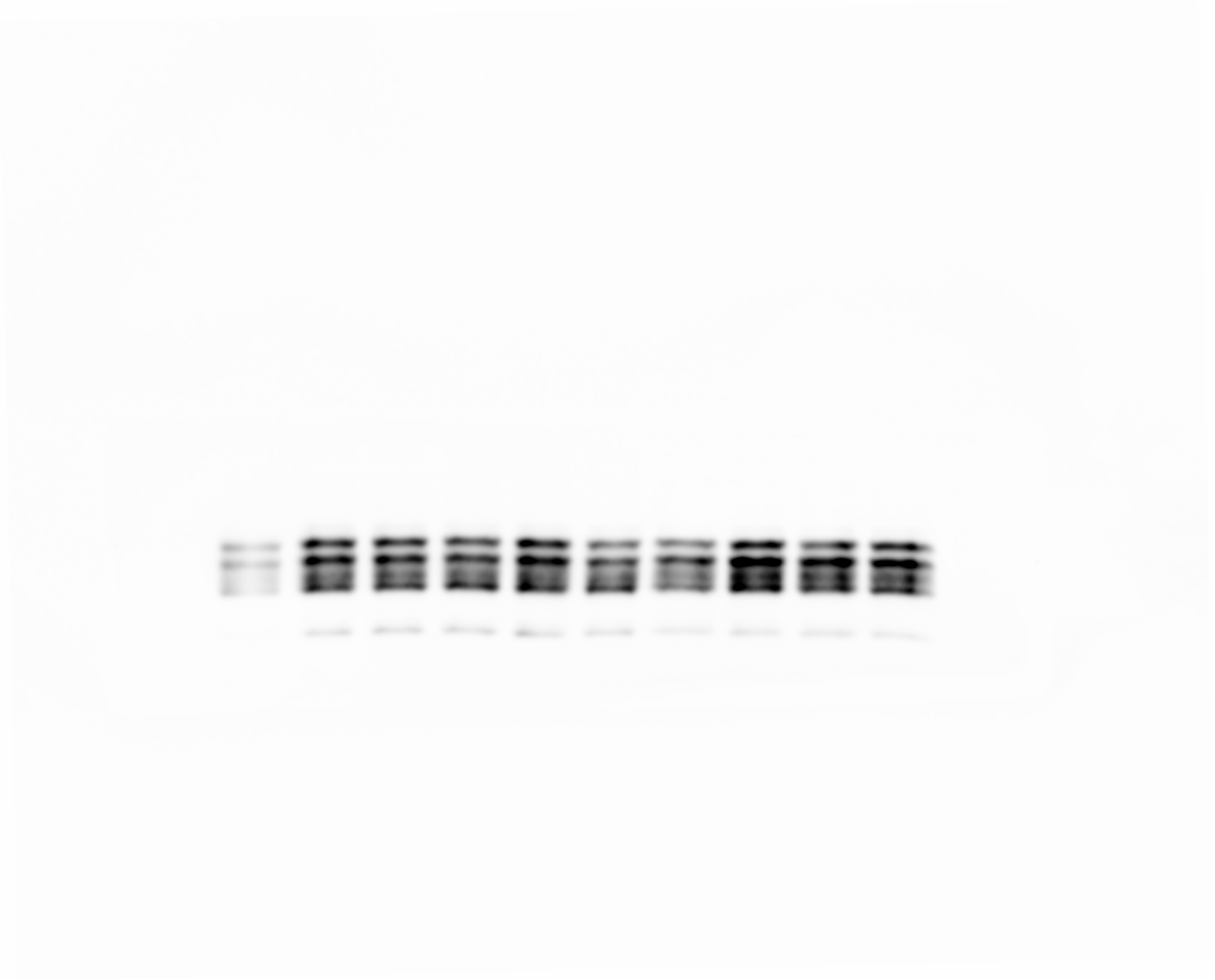

Supplement: Supplementary file 5 — Source data Fig. 3 [file 44321_2025_249_MOESM5_ESM.zip › Figure 3/3A_blot_p_4ebp1.tif]

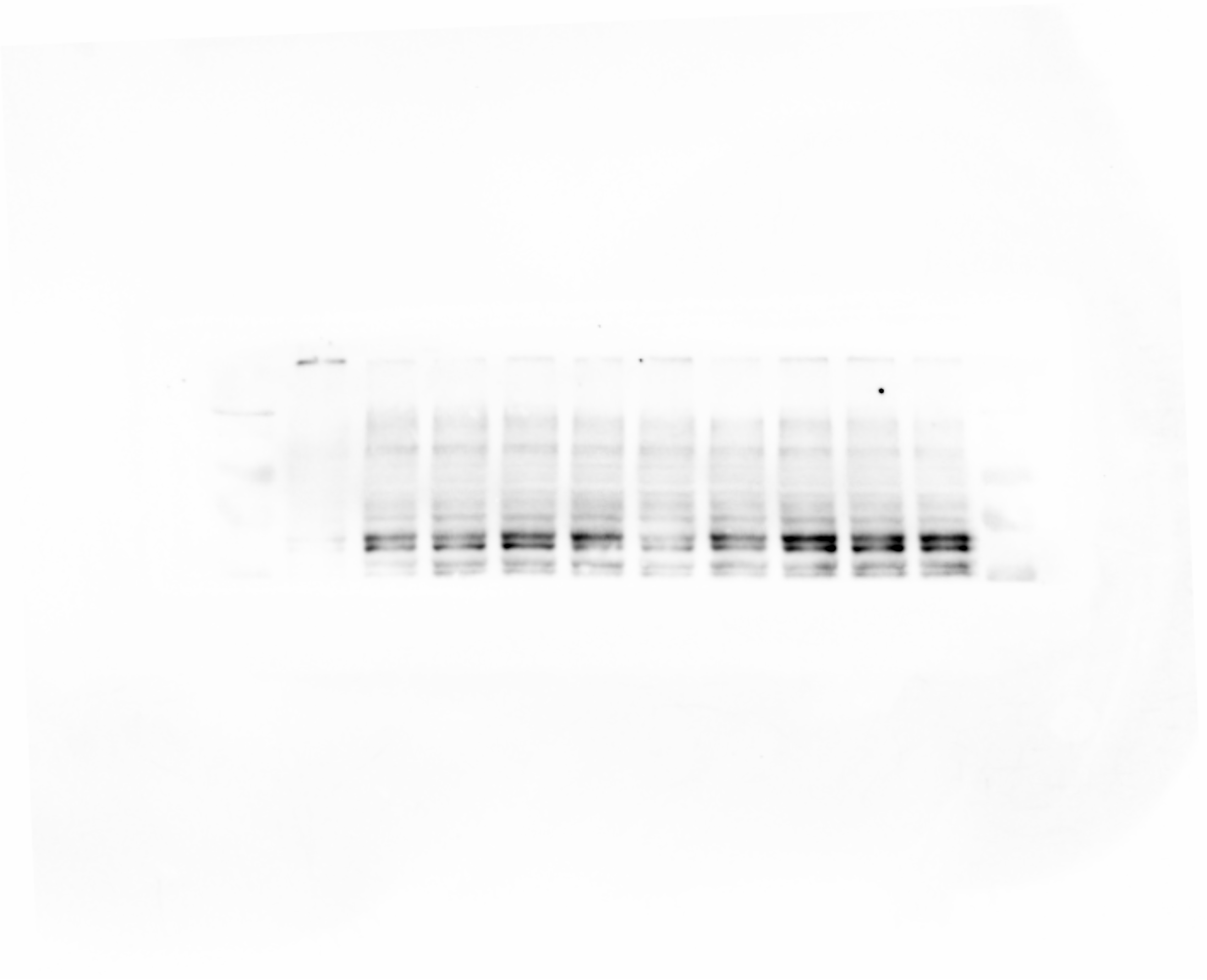

Supplement: Supplementary file 5 — Source data Fig. 3 [file 44321_2025_249_MOESM5_ESM.zip › Figure 3/3A_blot_p_70s6k.tif]

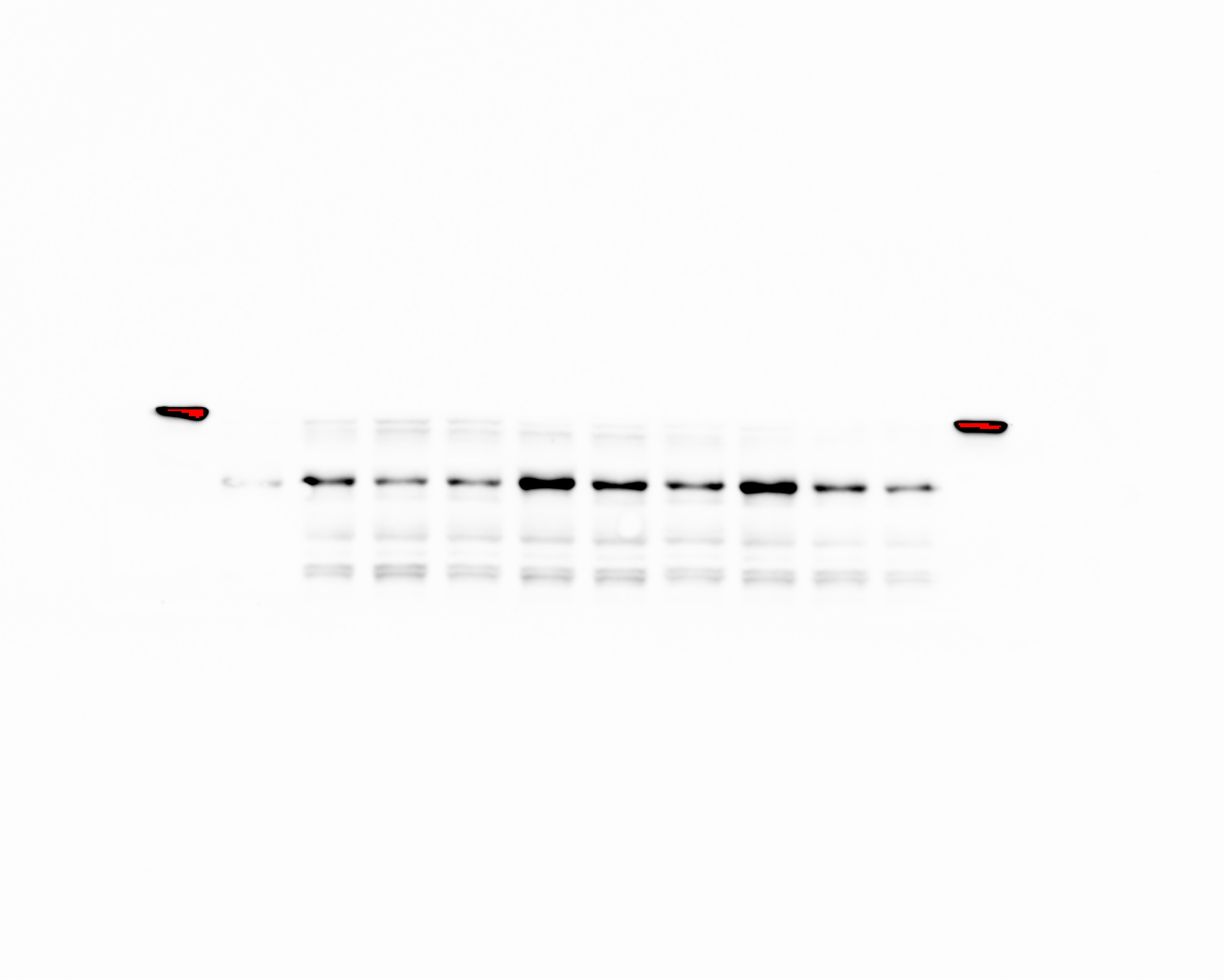

Supplement: Supplementary file 5 — Source data Fig. 3 [file 44321_2025_249_MOESM5_ESM.zip › Figure 3/3A_blot_p_pras40.tif]

### Figure 3A – Blot – P-PRAS40

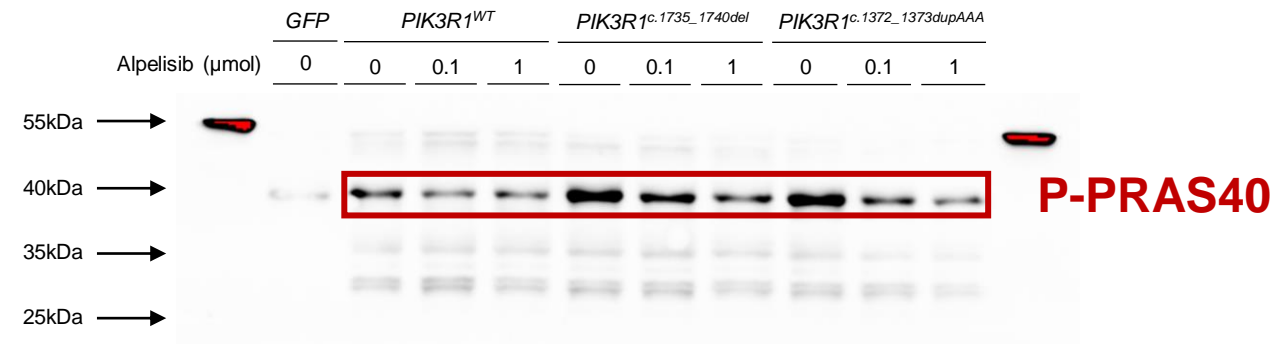

### Figure 3A – Blot – P-p70S6K

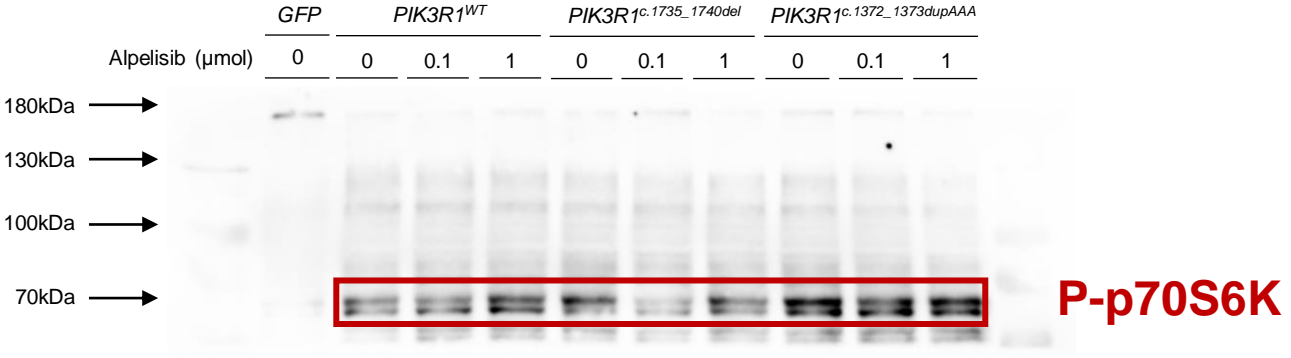

Figure 3A – Blot – P-4eBP1

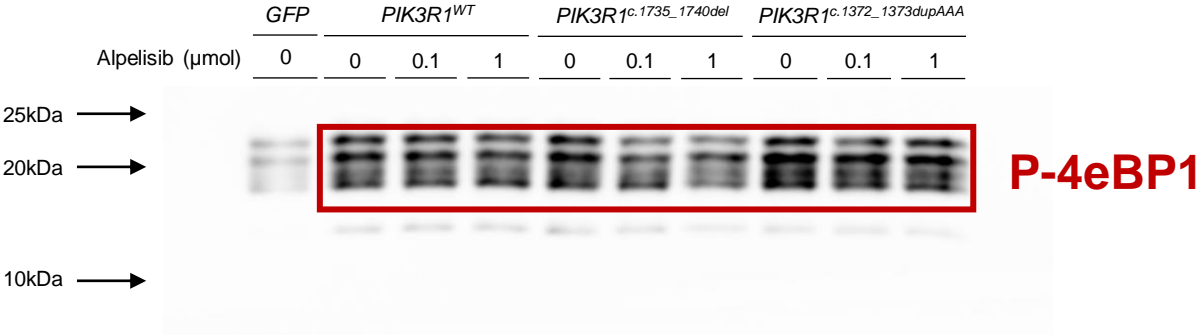

### Figure 3A – Blot – $\beta$ -actin

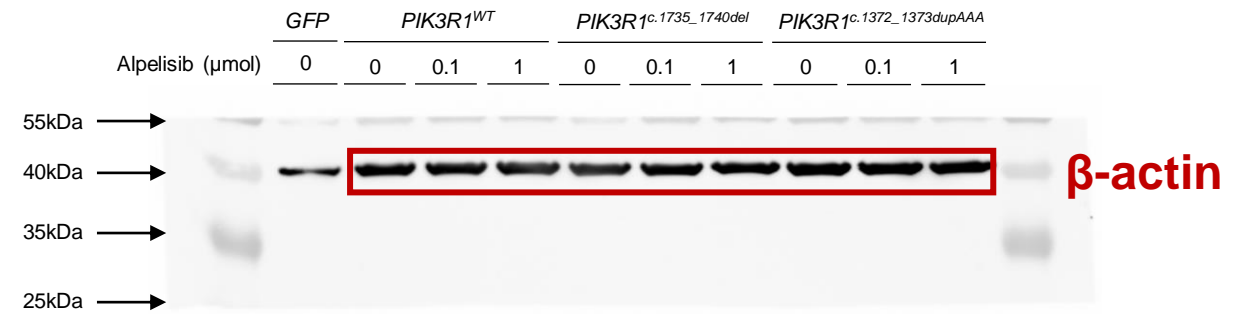

Supplement: Supplementary file 5 — Source data Fig. 3 [file 44321_2025_249_MOESM5_ESM.zip › Figure 3/3A_blot_summary.pdf]

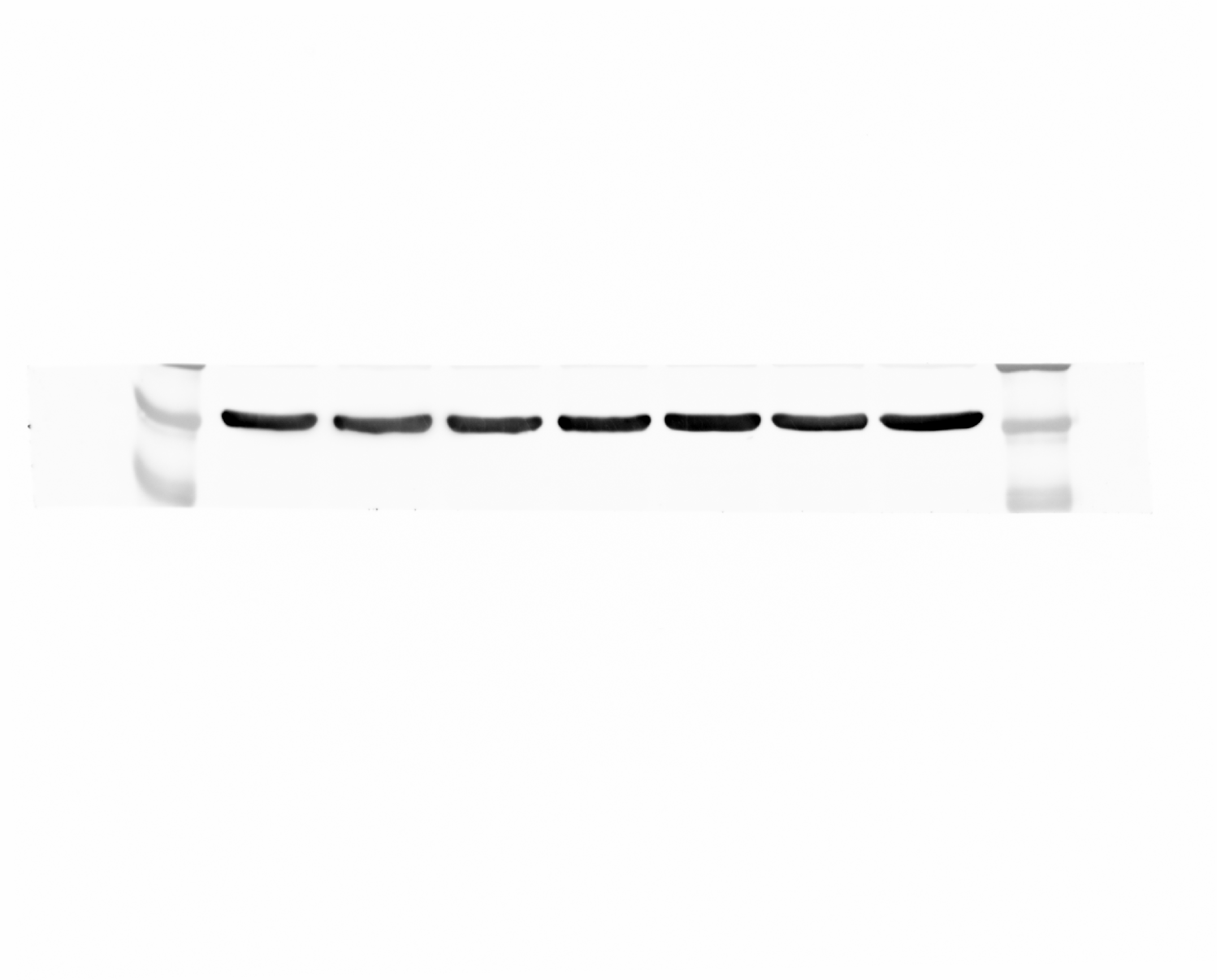

Supplement: Supplementary file 5 — Source data Fig. 3 [file 44321_2025_249_MOESM5_ESM.zip › Figure 3/3B_blot_actin.tif]

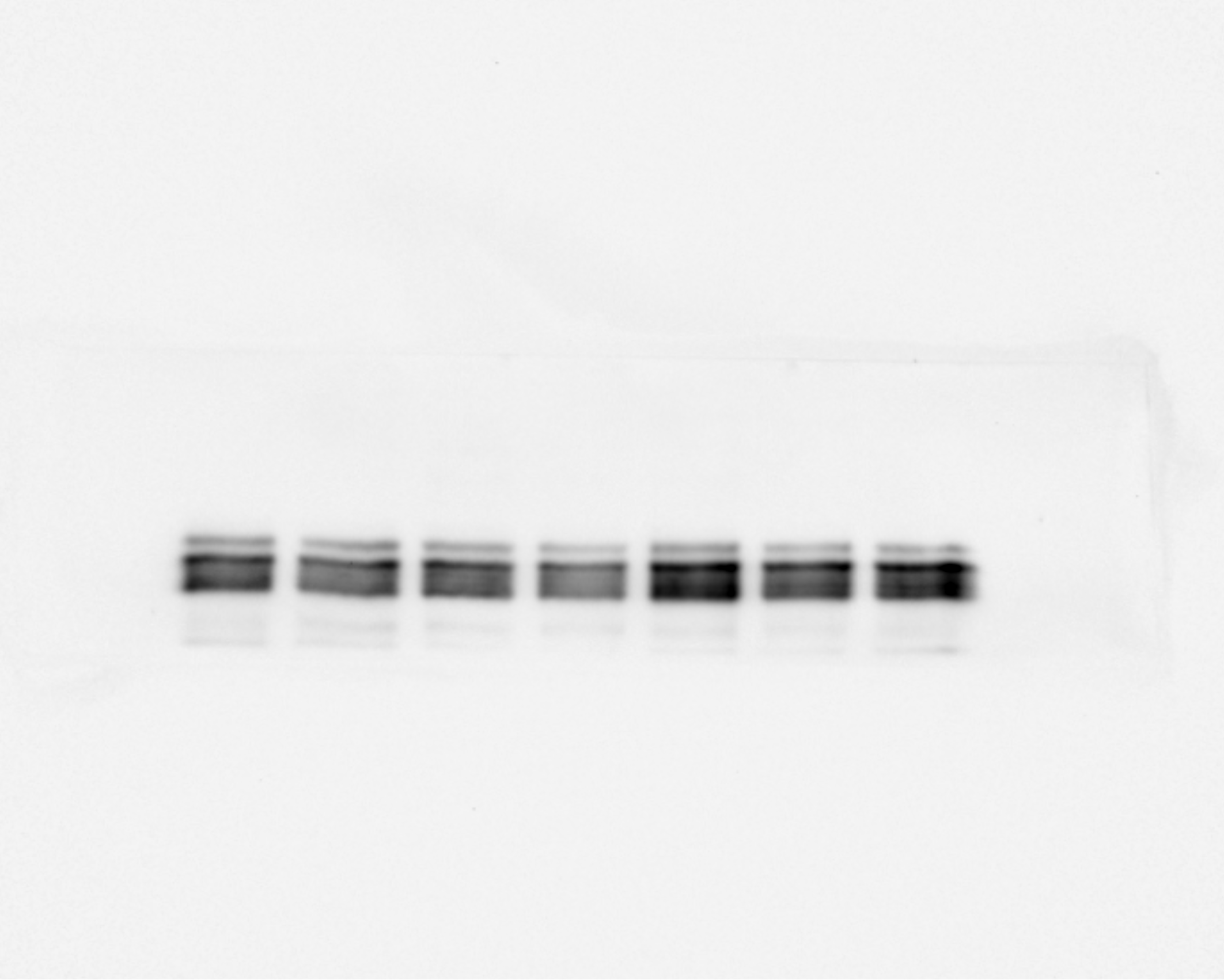

Supplement: Supplementary file 5 — Source data Fig. 3 [file 44321_2025_249_MOESM5_ESM.zip › Figure 3/3B_blot_p_4ebp1.tif]

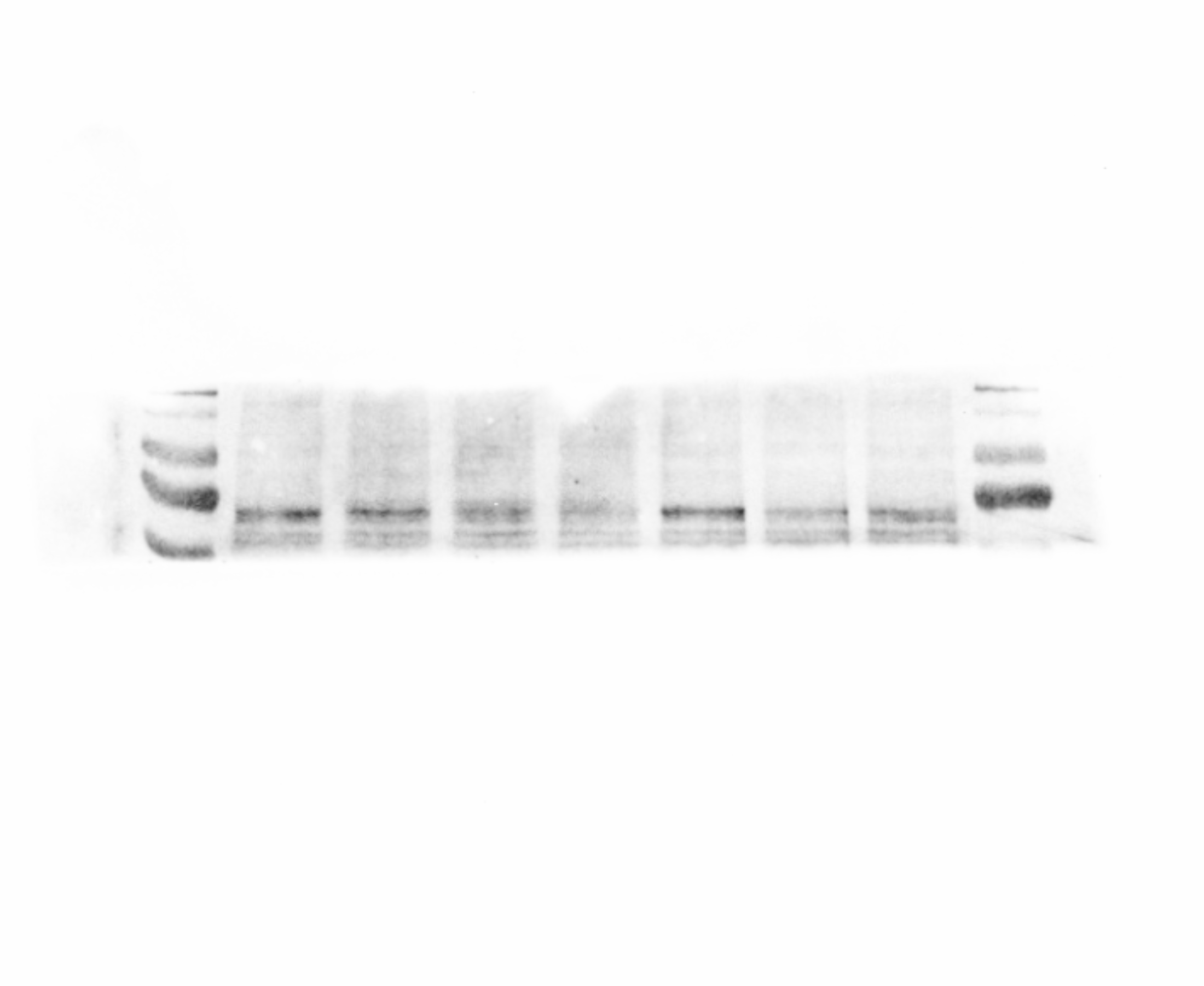

Supplement: Supplementary file 5 — Source data Fig. 3 [file 44321_2025_249_MOESM5_ESM.zip › Figure 3/3B_blot_p_p70s6k.tif]

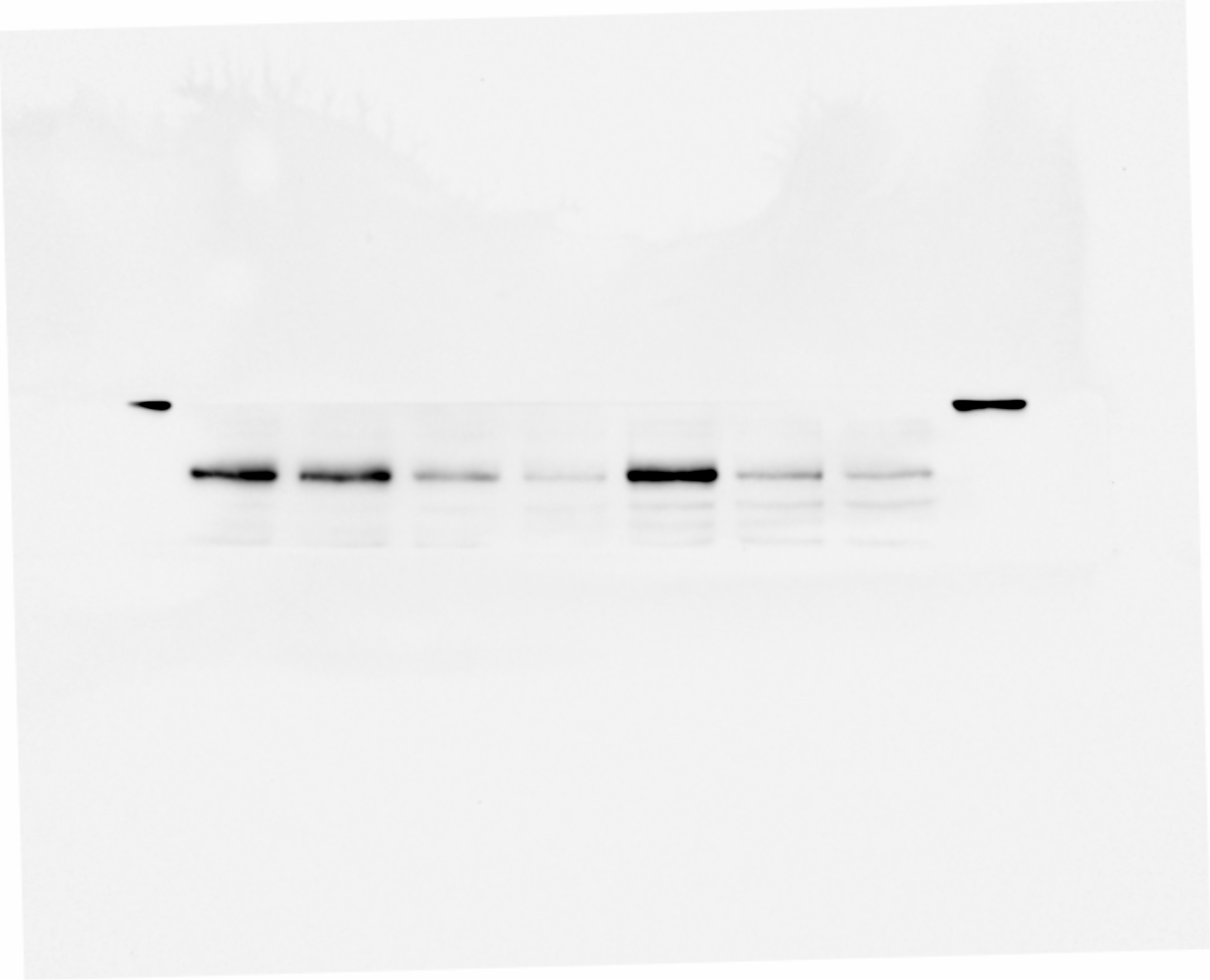

Supplement: Supplementary file 5 — Source data Fig. 3 [file 44321_2025_249_MOESM5_ESM.zip › Figure 3/3B_blot_p_pras40.tif]

Figure 3B – Blot – P-PRAS40

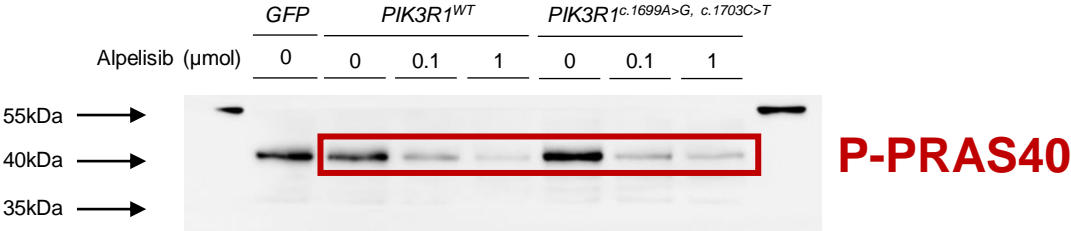

Figure 3B – Blot – P-p70S6K

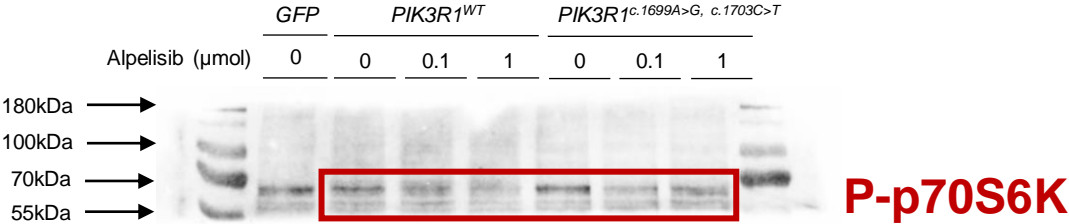

Figure 3B – Blot – P-4eBP1

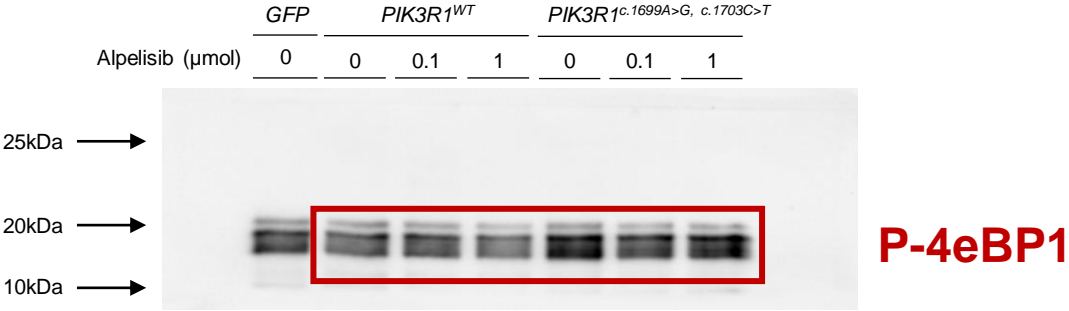

### Figure 3B – Blot – $\beta$ -actin

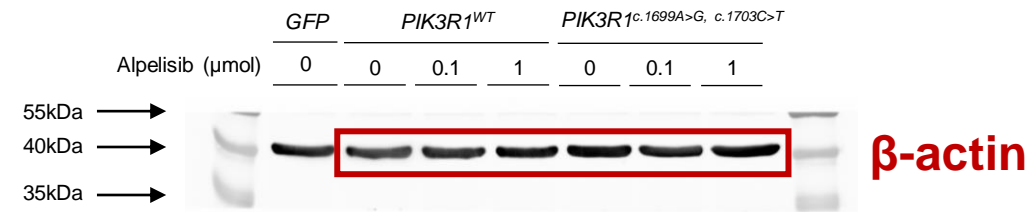

Supplement: Supplementary file 5 — Source data Fig. 3 [file 44321_2025_249_MOESM5_ESM.zip › Figure 3/3B_blot_summary.pdf]

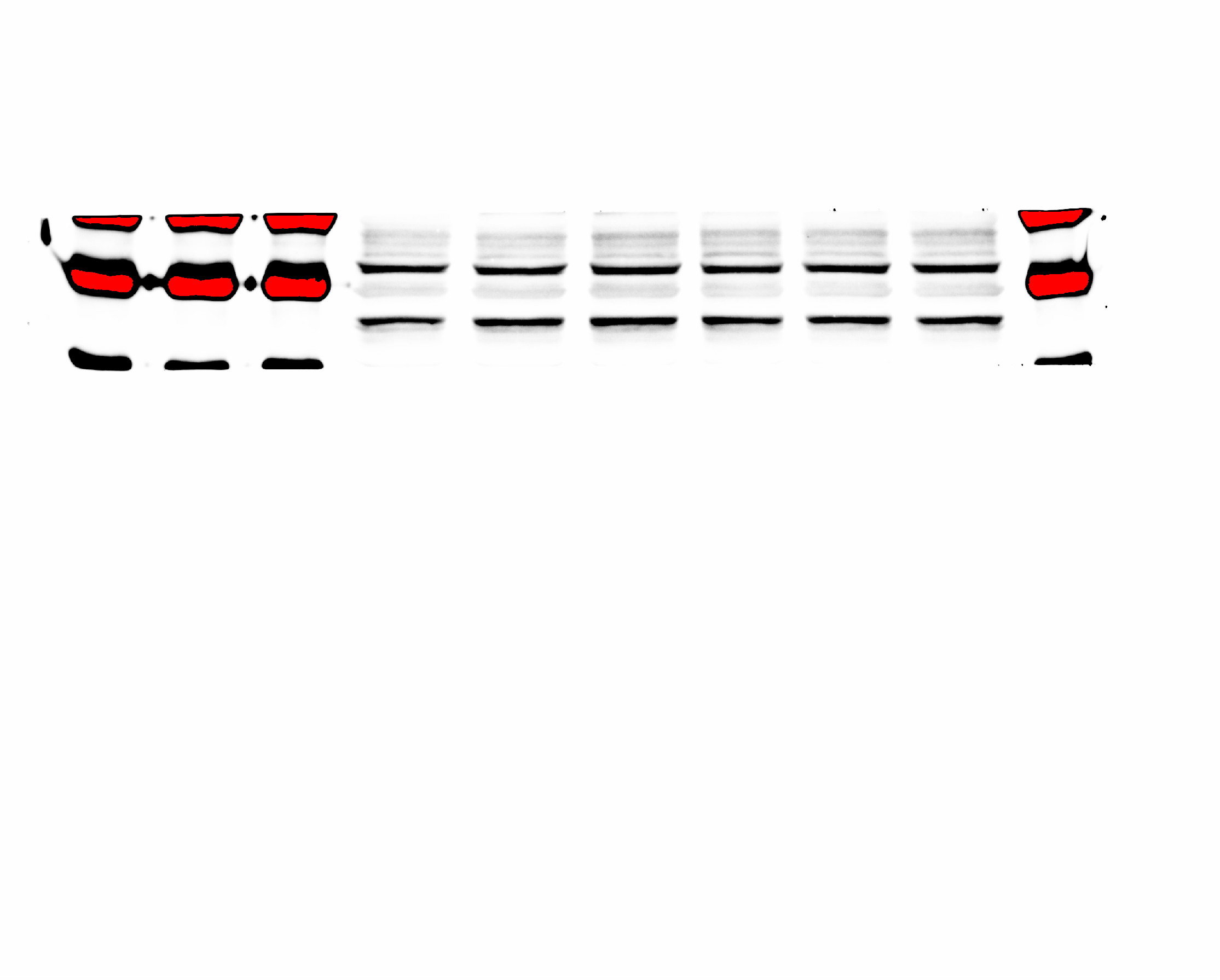

Supplement: Supplementary file 7 — Figure Source Data EV [file 44321_2025_249_MOESM7_ESM.zip › Figures EV/EV1_blot_akt.tif]

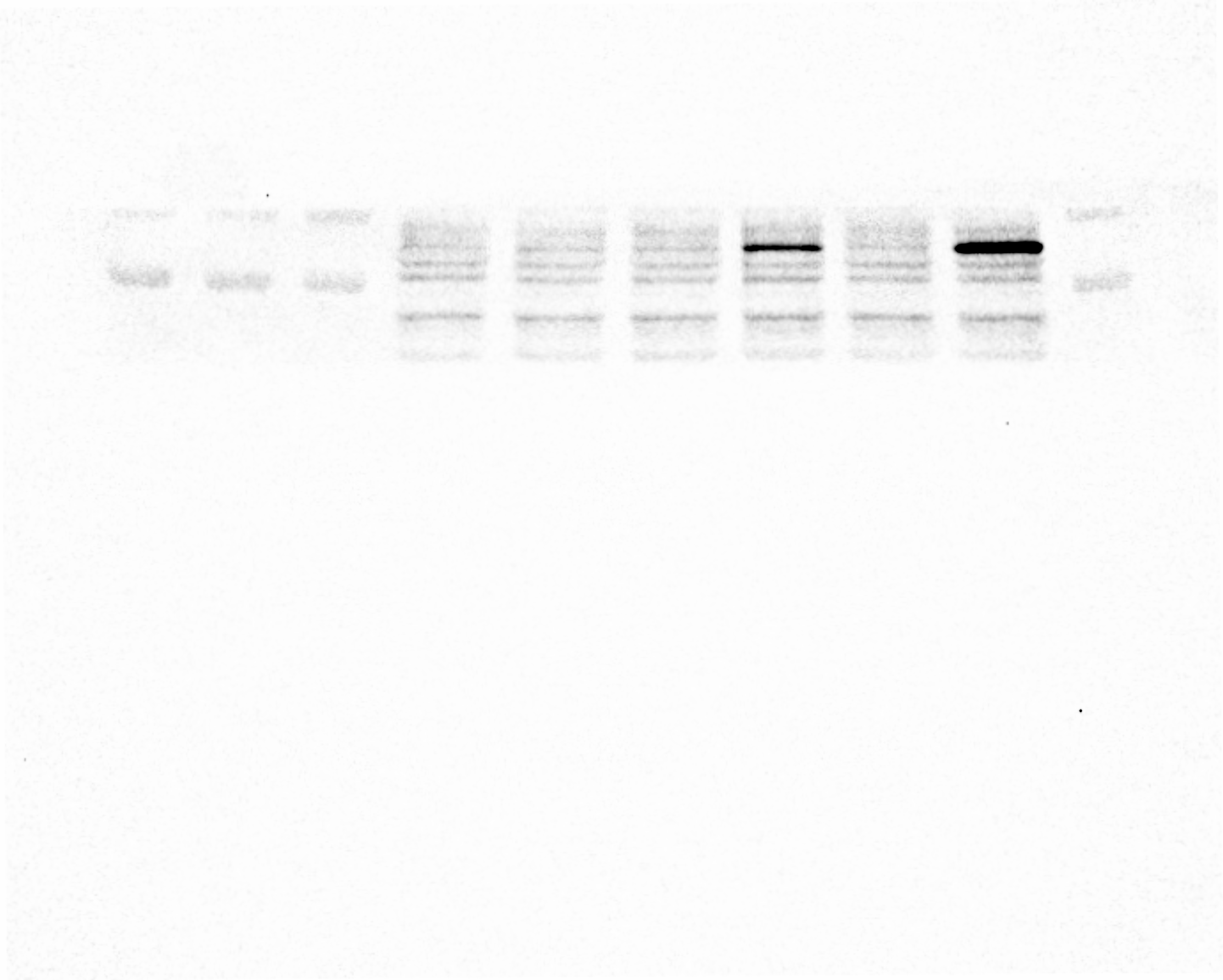

Supplement: Supplementary file 7 — Figure Source Data EV [file 44321_2025_249_MOESM7_ESM.zip › Figures EV/EV1_blot_p_akt.tif]

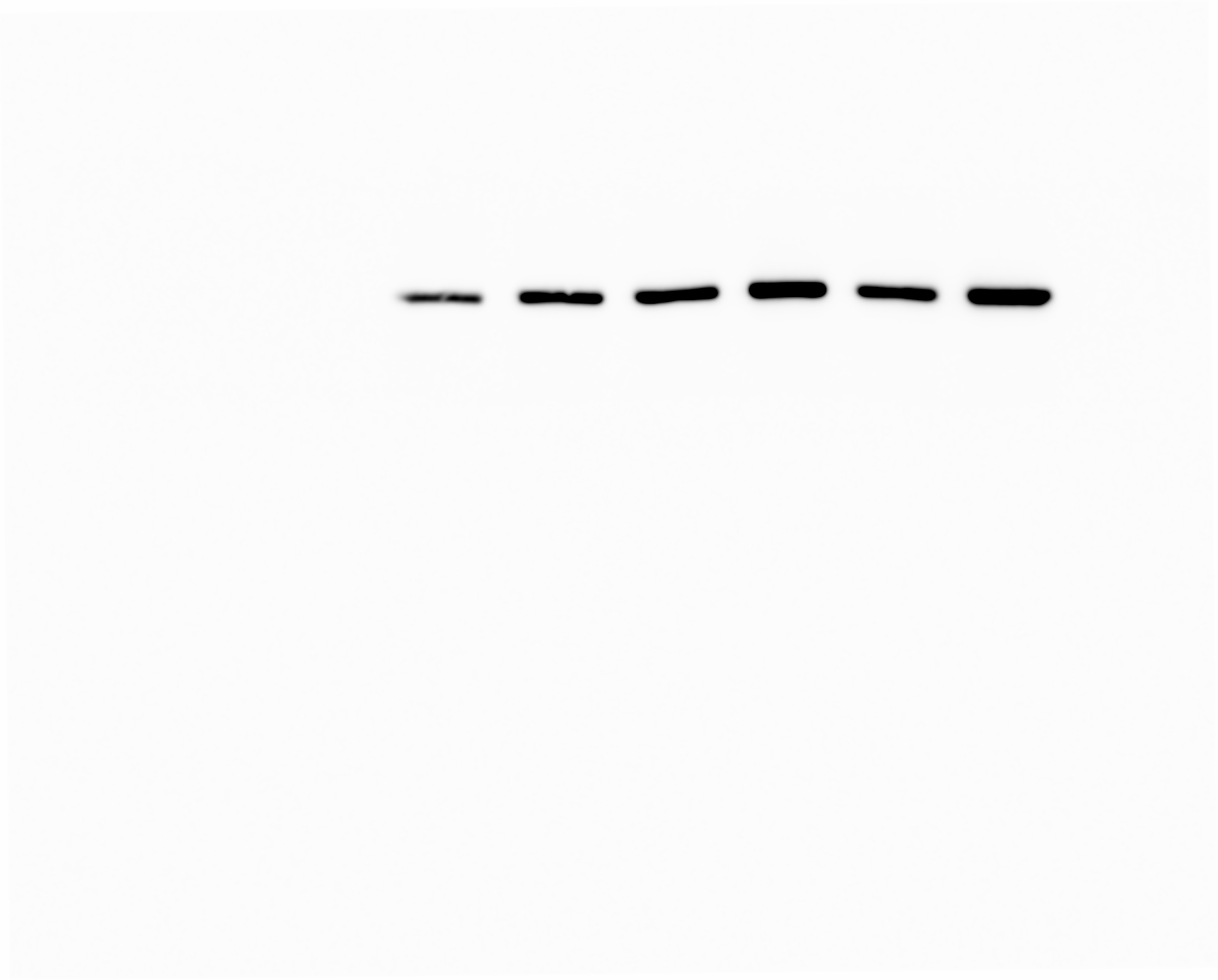

Supplement: Supplementary file 7 — Figure Source Data EV [file 44321_2025_249_MOESM7_ESM.zip › Figures EV/EV1_blot_p_s6rp.tif]

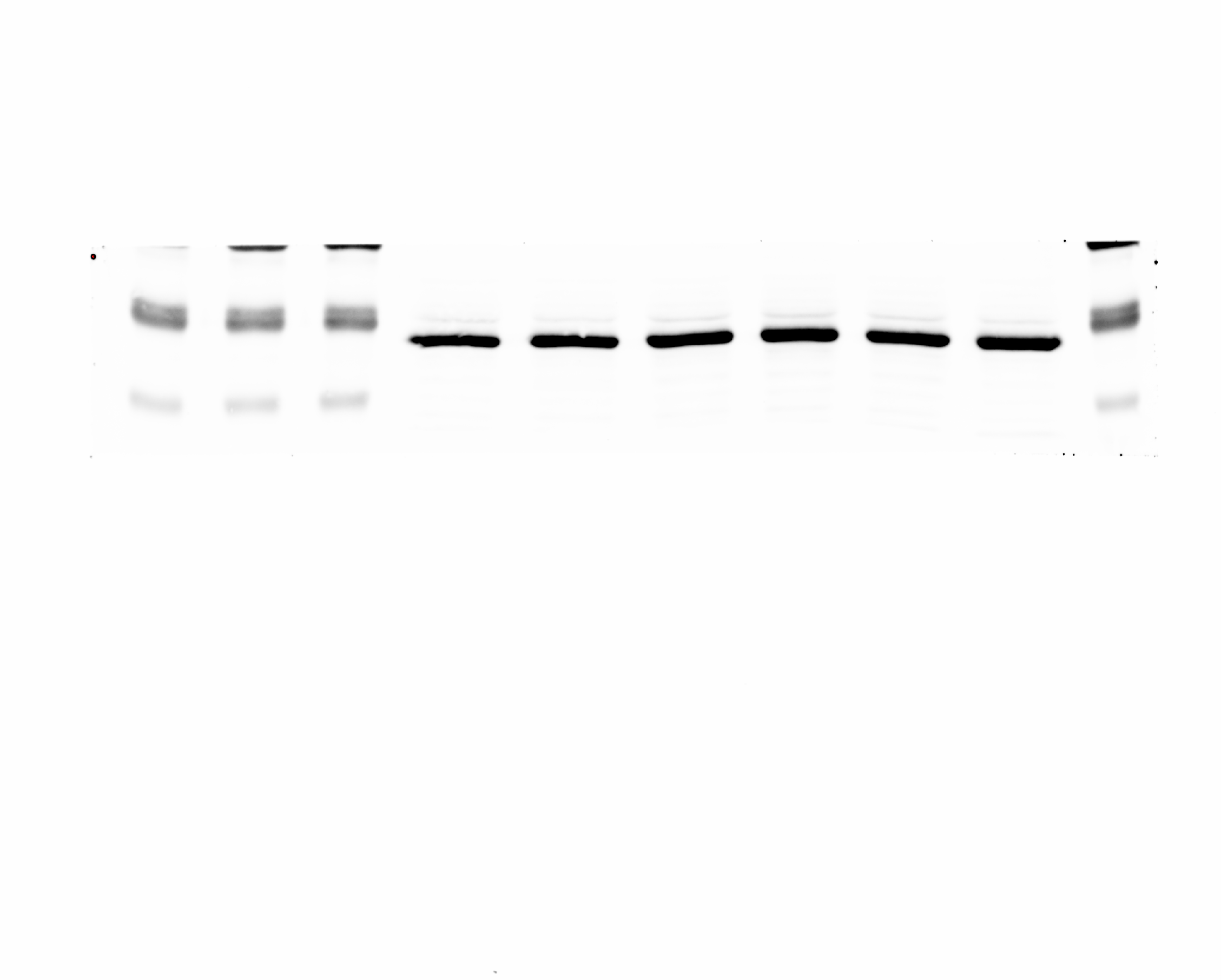

Supplement: Supplementary file 7 — Figure Source Data EV [file 44321_2025_249_MOESM7_ESM.zip › Figures EV/EV1_blot_s6rp.tif]

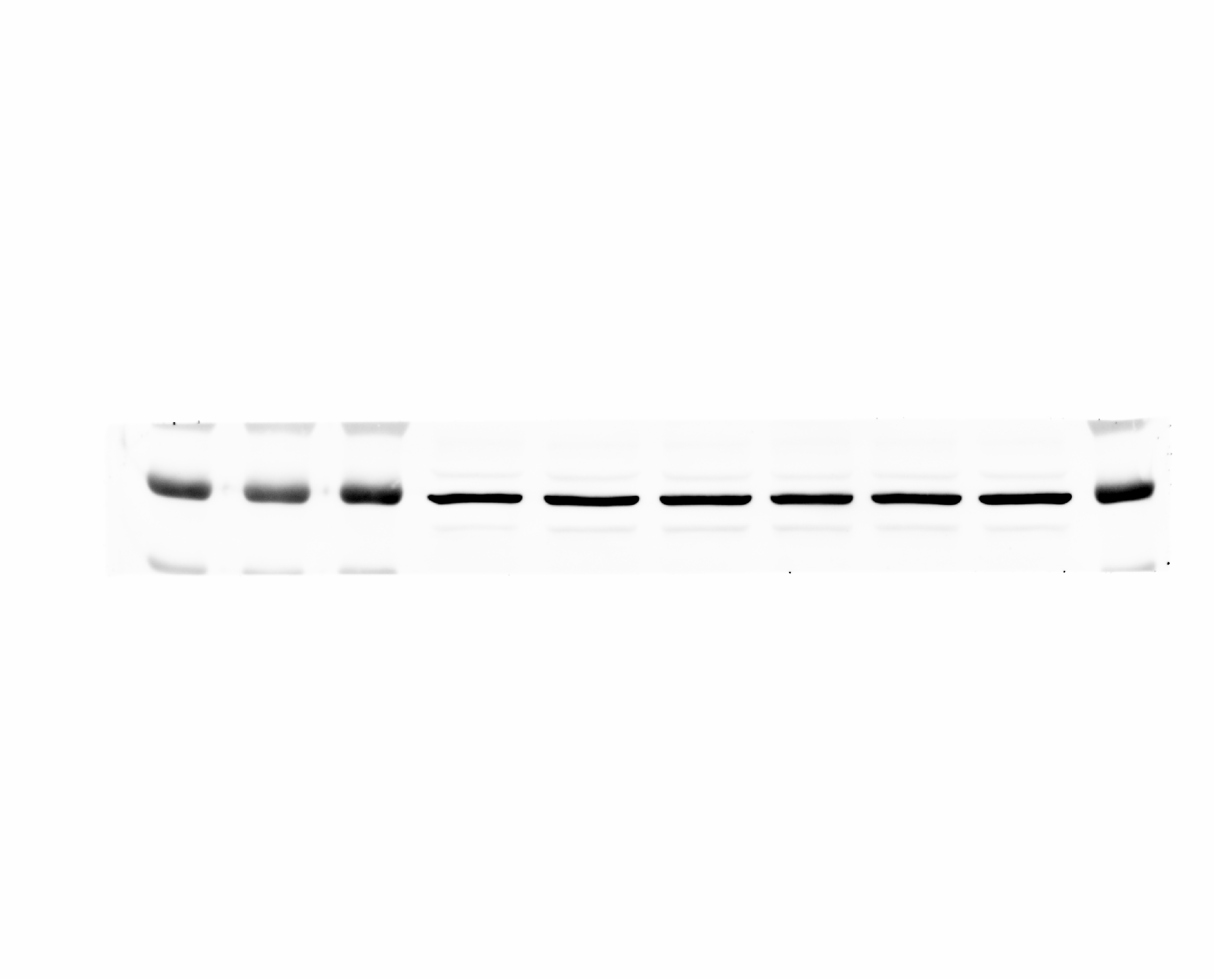

Supplement: Supplementary file 7 — Figure Source Data EV [file 44321_2025_249_MOESM7_ESM.zip › Figures EV/EV1_blot_tubulin.tif]

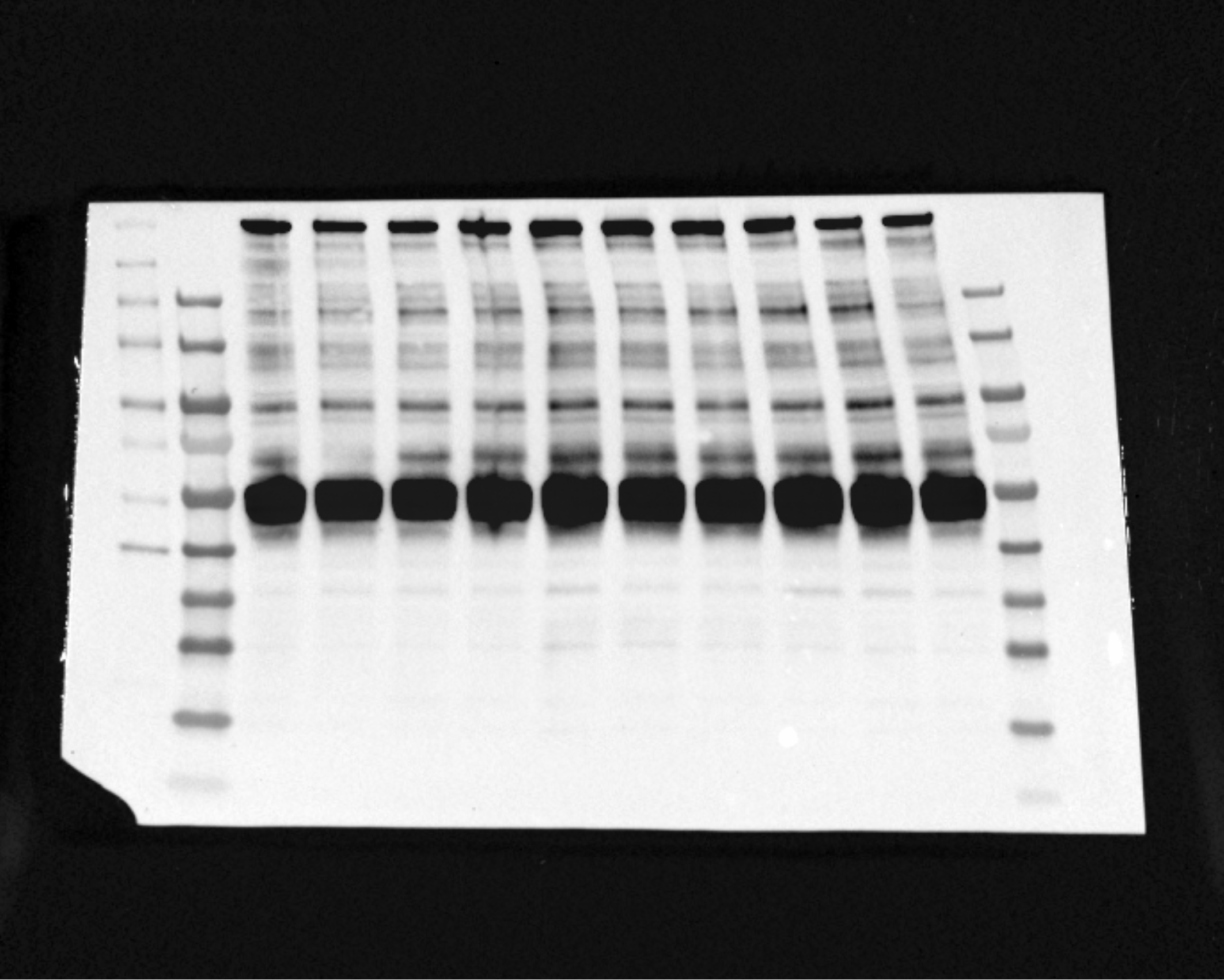

Supplement: Supplementary file 7 — Figure Source Data EV [file 44321_2025_249_MOESM7_ESM.zip › Figures EV/EV2A_blot_akt_substrates.tif]

Expanded View 2A – Blot – AKT substrates

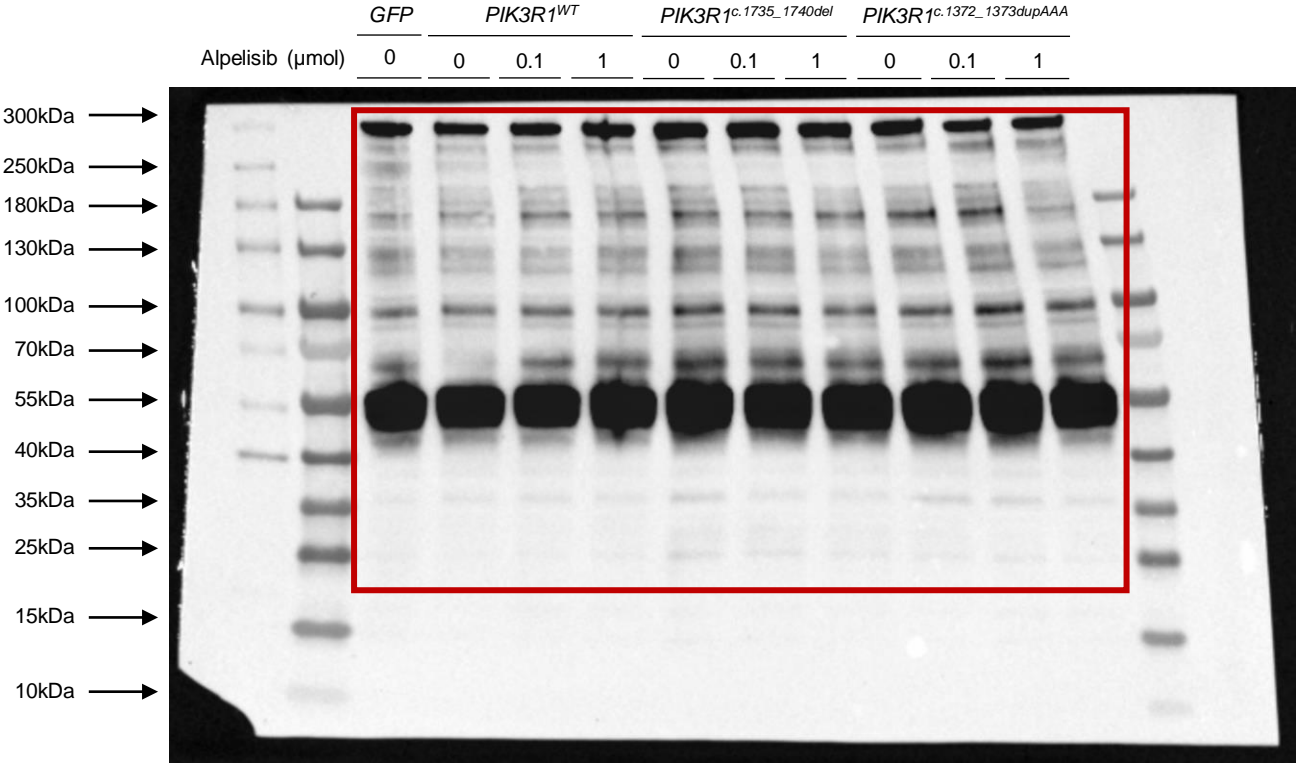

**AKT substrates  
(RXXS\*/T\*)**

Expanded View 2A – Blot –  $\alpha$ -tubulin

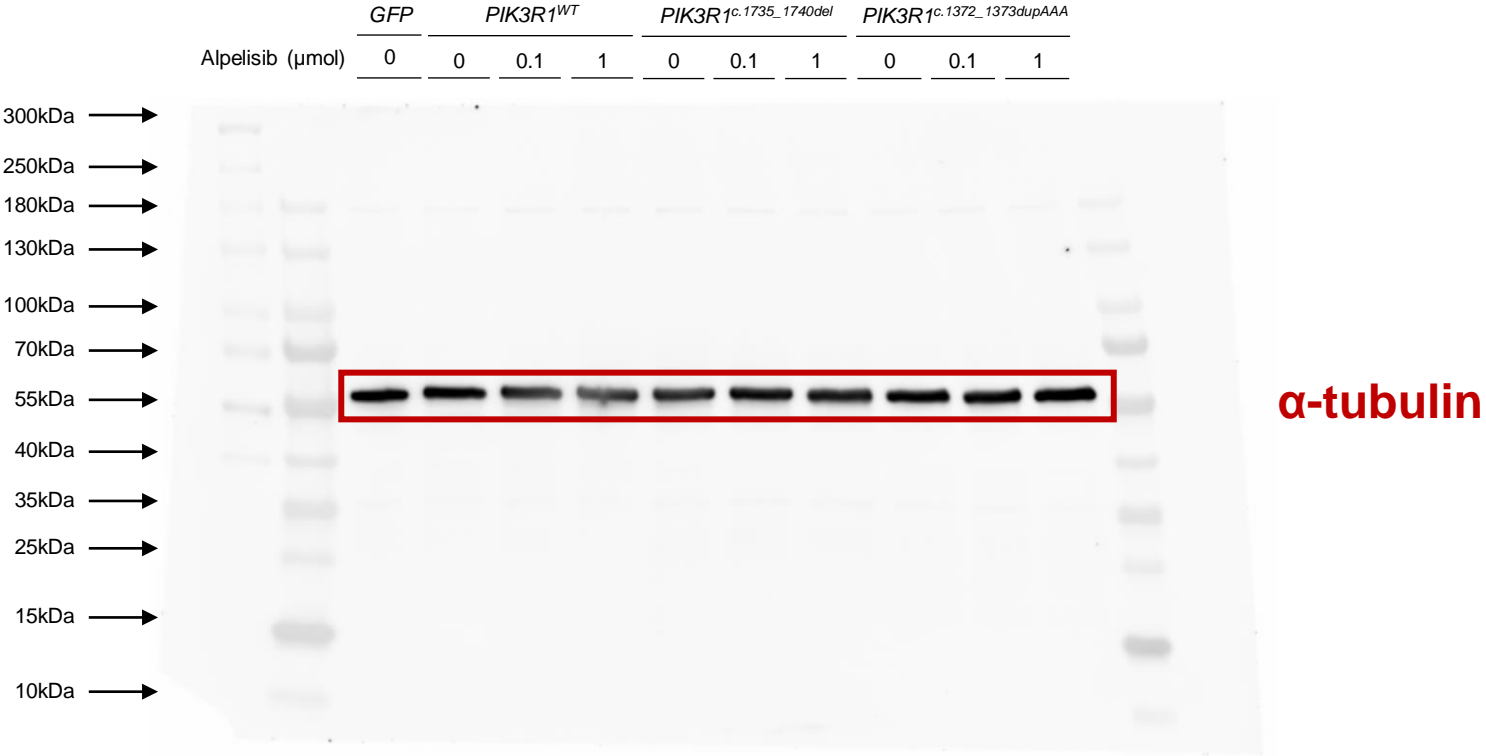

Supplement: Supplementary file 7 — Figure Source Data EV [file 44321_2025_249_MOESM7_ESM.zip › Figures EV/EV2A_blot_summary.pdf]

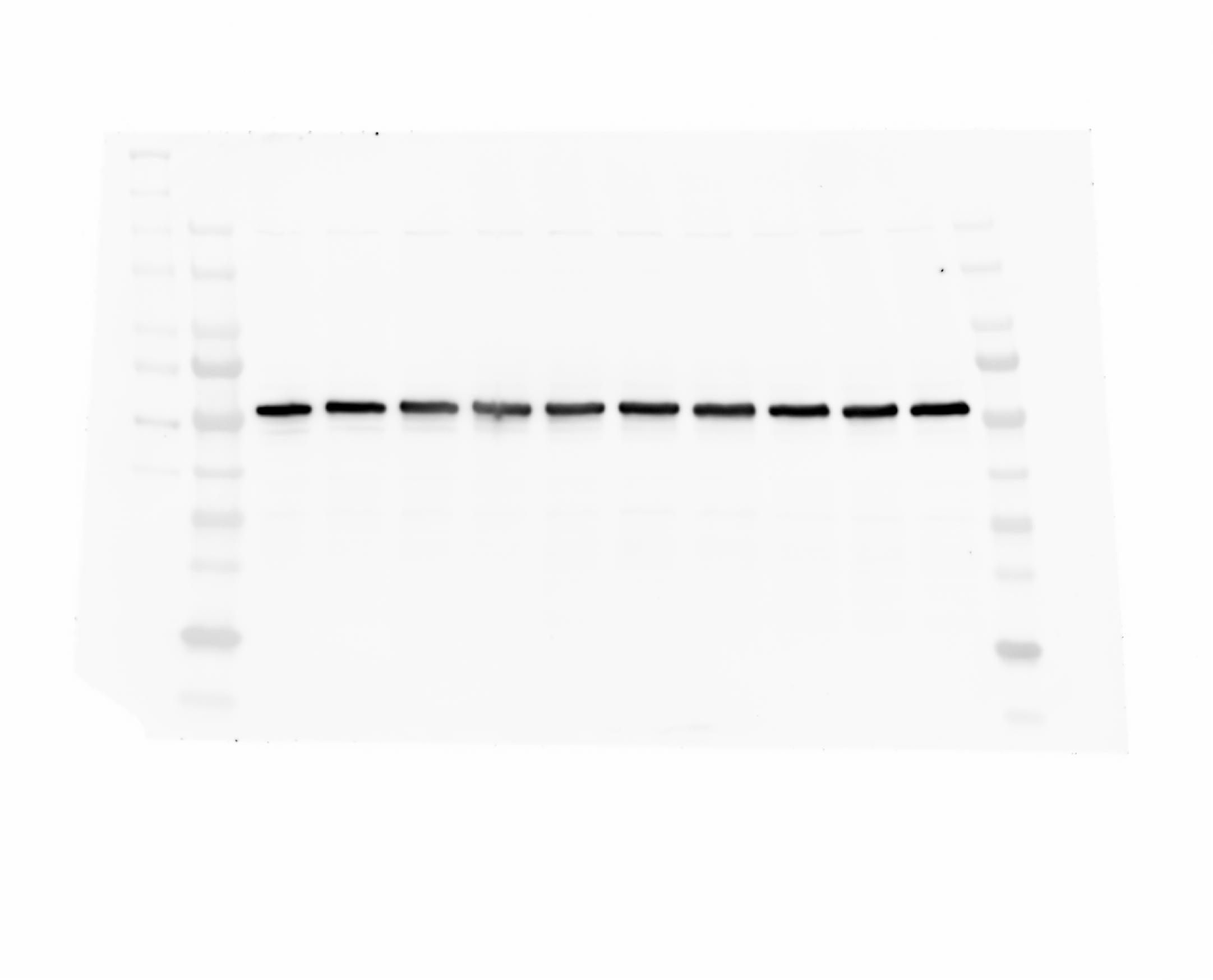

Supplement: Supplementary file 7 — Figure Source Data EV [file 44321_2025_249_MOESM7_ESM.zip › Figures EV/EV2A_blot_tubulin.tif]

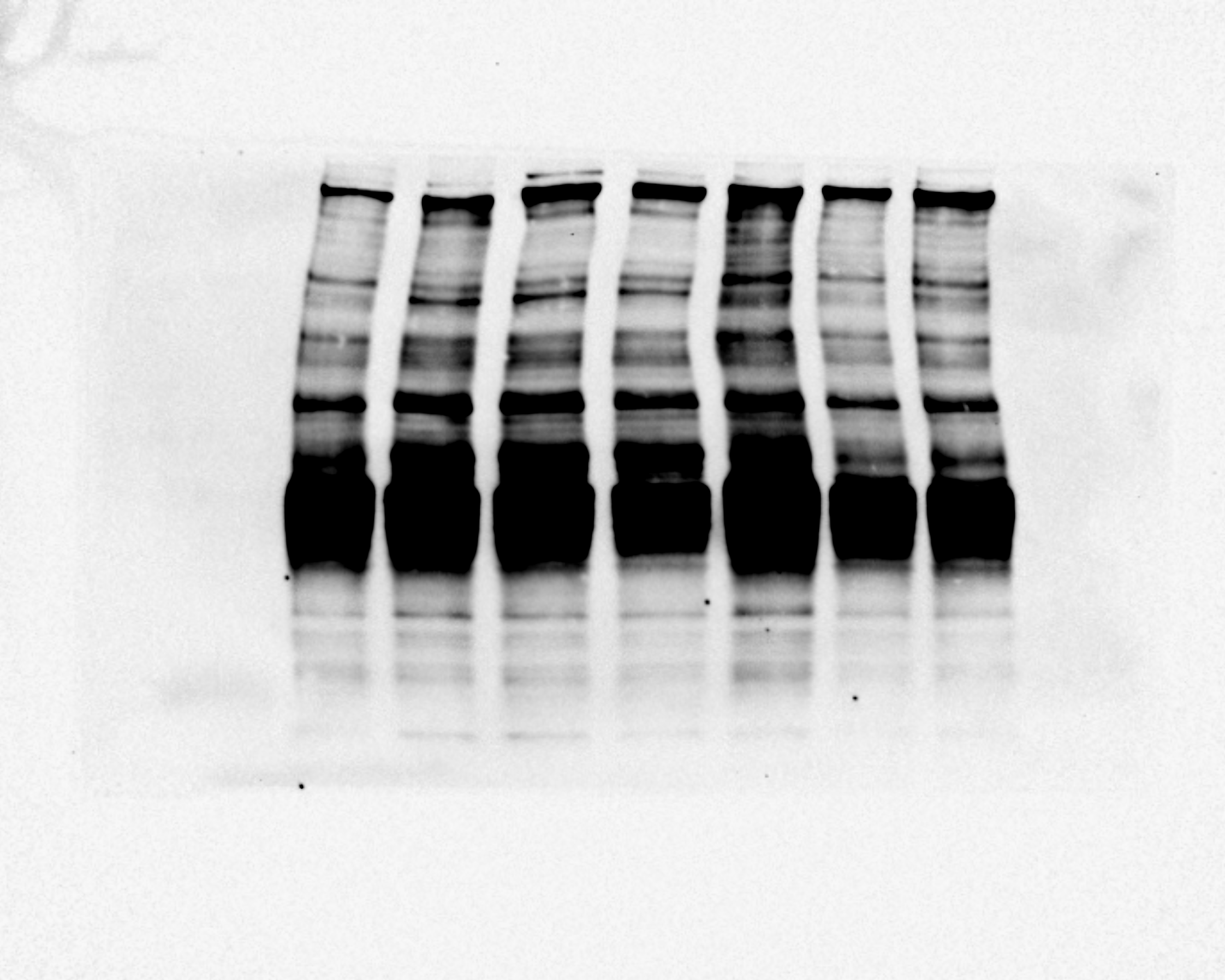

Supplement: Supplementary file 7 — Figure Source Data EV [file 44321_2025_249_MOESM7_ESM.zip › Figures EV/EV2B_blot_akt_substrates.tif]

Expanded View 2B – Blot – AKT substrates

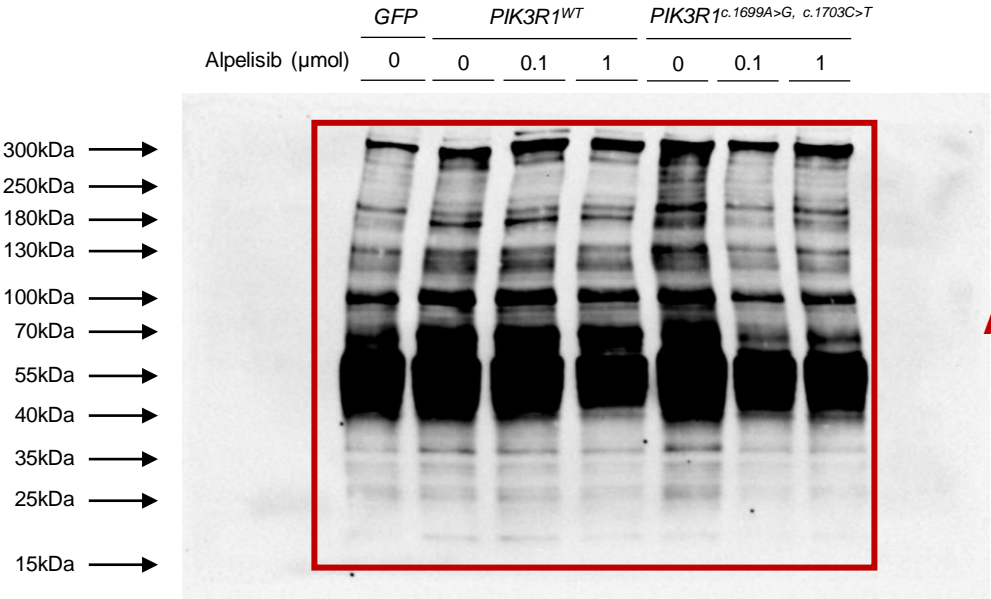

**AKT substrates  
(RXXS\*/T\*)**

Expanded View 2B – Blot –  $\alpha$ -tubulin

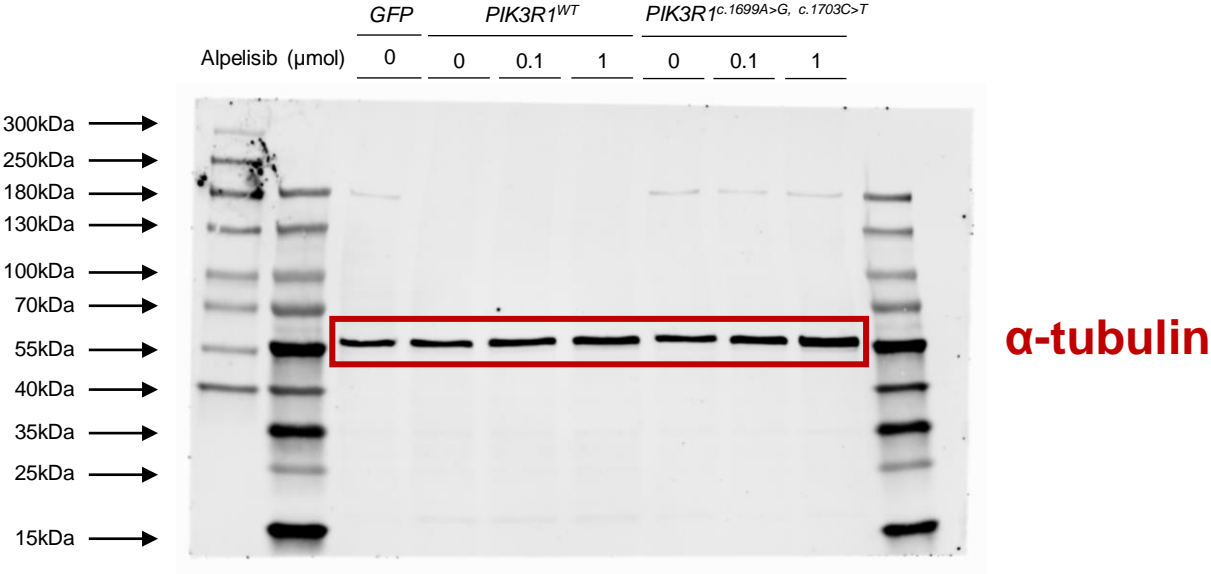

Supplement: Supplementary file 7 — Figure Source Data EV [file 44321_2025_249_MOESM7_ESM.zip › Figures EV/EV2B_blot_summary.pdf]

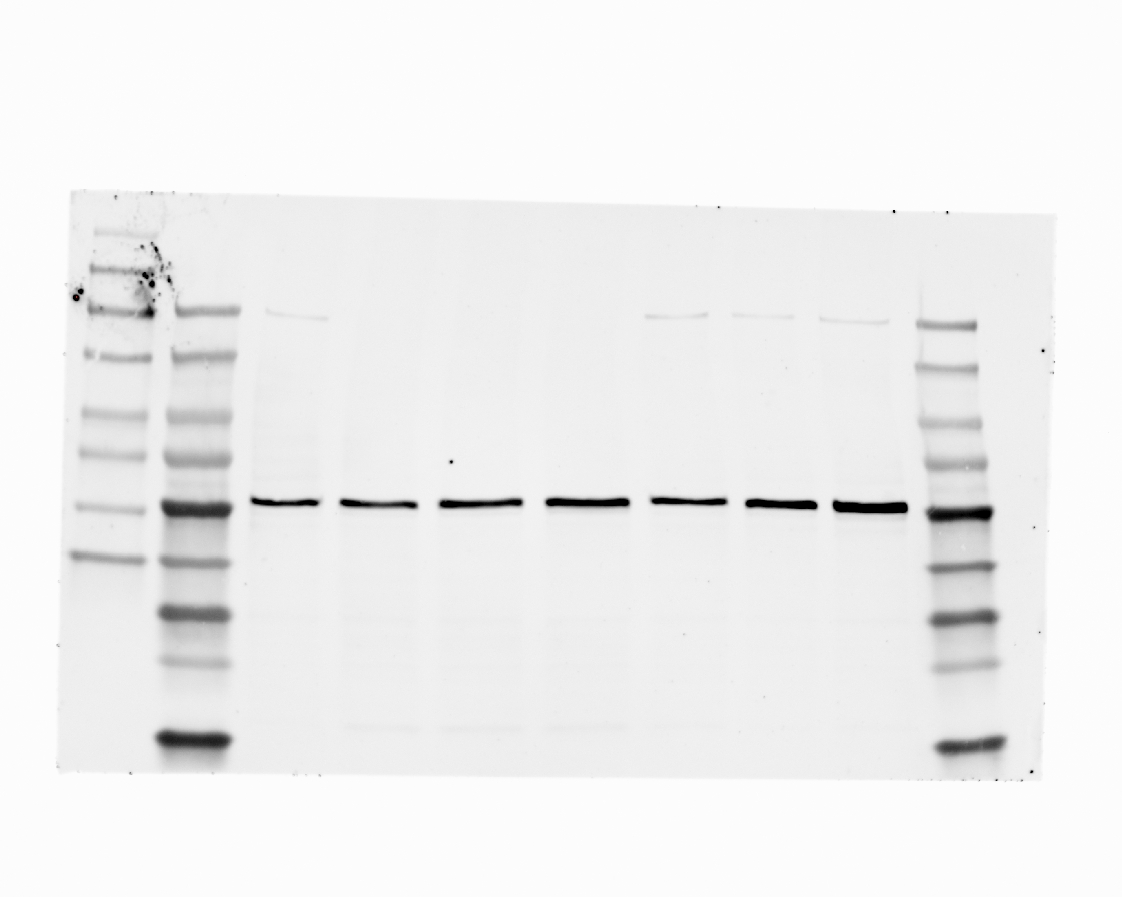

Supplement: Supplementary file 7 — Figure Source Data EV [file 44321_2025_249_MOESM7_ESM.zip › Figures EV/EV2B_blot_tubulin.jpg]
