# Supplementary material for: Somatic PIK3R1 mutations in the iSH2 domain are accessible to PI3Kα inhibition
Source: EMBO Mol Med. 2025 May 19;17(7):1556–74. doi: 10.1038/s44321-025-00249-9 (PMC12254339; doi:10.1038/s44321-025-00249-9)
Supplement: Supplementary file 7 — Figure Source Data EV [file 44321_2025_249_MOESM7_ESM.zip › Figures EV/EV3_alphafold_methods.pdf]

p85α-p110α interaction studies

Using Alphafold Server (AlphaFold3)

Methods

PROMPTS ON ALPHAFOLD SERVER

https://golgi.sandbox.google.com/

16-22/05/2024

SOURCE AMINO-ACID SEQUENCES

p110α wild-type (source UNIPROT P42336) : length 1068aa, predicted mass 124.284Da

```
   1    MPPRPSSGELWGIHLMPPRILVECLLPNGMIVTLECLREATLITIKHELF
  51    KEARKYPLHQLLQDESSYIFVSVTQEAEREEFFDETRRLCDLRLFQPFLK
 101    VIEPVGNREEKILNREIGFAIGMPVCEFDMVKDPEVQDFRRNILNVCKEA
 151    VDLRDLNSPHSRAMYVYPPNVESSPELPKHIYNKLDKGQIIVVIWVIVSP
 201    NNDKQKYTLKINHDCVPEQVIAEAIRKKTRSMLLSSEQLKLCVLEYQGKY
 251    ILKVCGCDEYFLEKYPLSQYKYIRSCIMLGRMPNLMLMAKESLYSQLPMD
 301    CFTMPSYSRRISTATPYMNGETSTKSLWVINSALRIKILCATYVNVNIRD
 351    IDKIYVRTGIYHGGEPLCDNVNTQRVPCSNPRWNEWLNYDIYIPDLPRAA
 401    RLCLSICSVKGRKGAKEEHCPLAWGNINLFDYTDTLVSGKMALNLWPVPH
 451    GLEDLLNPIGVTGSNPNKETPCLELEFDWFSSVVKFPDMSVIEEHANWSV
 501    SREAGFSYSHAGLSNRLARDNELRENDKEQLKAISTRDPLSEITEQEKDF
 551    LWSHRHYCVTIPEILPKLLLSVKWNSRDEVAQMYCLVKDWPPIKPEQAME
 601    LLDCNYPDPMVRGFAVRCLEKYLTDDKLSQYLIQLVQVLKYEQYLDNLLV
 651    RFLLKKALTNQRIGHFFFWHLKSEMHNKTVSQRFGLLLESYCRACGMYLK
 701    HLNRQVEAMEKLINLTDILKQEKKDETQKVQMKFLVEQMRRPDFMDALQG
 751    FLSPLNPAHQLGNLRLEECRIMSSAKRPLWLNWENPDIMSELLFQNNEII
 801    FKNGDDLRQDMLTLQIIRIMENIWQNQGLDLRMLPYGCLSIGDCVGLIEV
 851    VRNSHTIMQIQCKGGLKGALQFNSHTLHQWLKDKNKGEIYDAAIDLFTRS
 901    CAGYCVATFILGIGDRHNSNIMVKDDGQLFHIDFGHFLDHKKKKFGYKRE
 951    RVPFVLTQDFLIVISKGAQECTKTREFERFQEMCYKAYLAIRQHANLFIN
1001    LFSMMLGSGMPELQSFDDIAYIRKTLALDKTEQEALEYFMKQMNDAHHGG
1051    WTTKMDWIFHTIKQHALN
```

p85α wild-type (source UNIPROT A0A2X0SFG1) : length 724aa, predicted mass 83,598Da

1	MSAEGYQYRALYDYKKEREEDIDLHLGDILTVNKGSLVALGFSDGQEARP
51	EEIGWLNGYNETTGERGDFPGTYVEYIGRKKISPPTPKPRPPRPLPVAPG
101	SSKTEADVEQQALTLPDLAEQFAPPDIAPPLLIKLVEAIEKKGLECSTLY
151	RTQSSSNLAELRQLLDCDTPSVDLEMIDVHVLADAFKRYLLDLPNPVIPA
201	AVYSEMISLAPEVQSSEEYIQLLKKLIRSPSIPHQYWLTLQYLLKHFFKL
251	SQTSSKNLLNARVLSEIFSPMLFRFSAASSDNTENLIKVIEILISTEWNE
301	RQPAPALPPKPPKPTTVANNGMNNNMSLQDAEWYWGDISREEVNEKLRDT
351	ADGTFLVRDASTKMHGDYTLTLRKGGNNKLIKIFHRDGKYGFSDPLTFSS
401	VVELINHYRNESLAQYNPKLDVKLLYPVSKYQQDQVVKEDNIEAVGKKLH
451	EYNTQFQEKSREYDRLYEEYTRTSQEIQMKRTAIEAFNETIKIFEEQCQT
501	QERYSKEYIEKFKREGNEKEIQRIMHNYDKLKSRISEIIDSRRRLEEDLK
551	KQAAEYREIDKRMNSIKPDLIQLRKTRDQYLMWLTQKGVRQKKLNEWLGN
601	ENTEDQYSLVEDDEDLPHHDEKTWNVGSSNRNKAENLLRGKRDGTFLVRE
651	SSKQGCYACSVVVDGEVKHCVINKTATGYGFAEPYNLYSSLKELVLHYQH
701	TSLVQHNDSLNVTLAYPVYAQQRR

p85α p.(K567E, P568L)

1 MSAEGYQYRALYDYKKEREEDIDLHLGDILTVNKGSLVALGFSDGQEARP
51 EEIGWLNGYNETTGERGDFPGTYVEYIGRKKISPPTPKPRPPRPLPVAPG
101 SSKTEADVEQQALTLPDLAEQFAPPDIAPPLLIKLVEAIEKKGLECSTLY
151 RTQSSSNLAELRQLLDCDTPSVDLEMIDVHVLADAFKRYLLDLPNPVIPA
201 AVYSEMISLAPEVQSSEEYIQLLKKLIRSPSIPHQYWLTLQYLLKHFFKL
251 SQTSSKNLLNARVLSEIFSPMLFRFSAASSDNTENLIKVIEILISTEWNE
301 RQPAPALPPKPPKPTTVANNGMNNNMSLQDAEWYWGDISREEVNEKLRDT
351 ADGTFLVRDASTKMHGDYTLTLRKGGNNKLIKIFHRDGKYGFSDPLTFSS
401 VVELINHYRNESLAQYNPKLDVKLLYPVSKYQQDQVVKEDNIEAVGKKLH
451 EYNTQFQEKSREYDRLYEEYTRTSQEIQMKRTAIEAFNETIKIFEEQCQT
501 QERYSKEYIEKFKREGNEKEIQRIMHNYDKLKSRISEIIDSRRRLEEDLK
551 KQAAEYREIDKRMNSI**EL**DLIQLRKTRDQYLMWLTQKGVRQKKLNEWLGN
601 ENTEDQYSLVEDDEDLPHHDEKTWNVGSSNRNKAENLLRGKRDGTFLVRE
651 SSKQGCYACSVVVDGEVKHCVINKTATGYGFAEPYNLYSSLKELVLHYQH
701 TSLVQHNDSLNVTLAYPVYAQQRR

p85α p.K459dup

1	MSAEGYQYRALYDYKKEREEDIDLHLGDILTVNKGSLVALGFSDGQEARP
51	EEIGWLNGYNETTGERGDFPGTYVEYIGRKKISPPTPKPRPPRPLPVAPG
101	SSKTEADVEQQALTLPDLAEQFAPPDIAPPLLIKLVEAIEKKGLECSTLY
151	RTQSSSNLAELRQLLDCDTPSVDLEMIDVHVLADAFKRYLLDLPNPVIPA
201	AVYSEMISLAPEVQSSEEYIQLLKKLIRSPSIPHQYWLTLQYLLKHFFKL
251	SQTSSKNLLNARVLSEIFSPMLFRFSAASSDNTENLIKVIEILISTEWNE
301	RQPAPALPPKPPKPTTVANNGMNNNMSLQDAEWYWGDISREEVNEKLRDT
351	ADGTFLVRDASTKMHGDYTLTLRKGGNNKLIKIFHRDGKYGFSDPLTFSS
401	VVELINHYRNESLAQYNPKLDVKLLYPVSKYQQDQVVKEDNIEAVGKKLH
451	EYNTQFQEK**K**SREYDRLYEEYTRTSQEIQMKRTAIEAFNETIKIFEEQCQT
501	QERYSKEYIEKFKREGNEKEIQRIMHNYDKLKSRISEIIDSRRRLEEDLK
551	KQAAEYREIDKRMNSIKPDLIQLRKTRDQYLMWLTQKGVRQKKLNEWLGN
601	ENTEDQYSLVEDDEDLPHHDEKTWNVGSSNRNKAENLLRGKRDGTFLVRE
651	SSKQGCYACSVVVDGEVKHCVINKTATGYGFAEPYNLYSSLKELVLHYQH
701	TSLVQHNDSLNVTLAYPVYAQQRR

p85α p.(579-580)del

1	MSAEGYQYRALYDYKKEREEDIDLHLGDILTVNKGSLVALGFSDGQEARP
51	EEIGWLNGYNETTGERGDFPGTYVEYIGRKKISPPTPKPRPPRPLPVAPG
101	SSKTEADVEQQALTLPDLAEQFAPPDIAPPLLIKLVEAIEKKGLECSTLY
151	RTQSSSNLAELRQLLDCDTPSVDLEMIDVHVLADAFKRYLLDLPNPVIPA
201	AVYSEMISLAPEVQSSEEYIQLLKKLIRSPSIPHQYWLTLQYLLKHFFKL
251	SQTSSKNLLNARVLSEIFSPMLFRFSAASSDNTENLIKVIEILISTEWNE
301	RQPAPALPPKPPKPTTVANNGMNNNMSLQDAEWYWGDISREEVNEKLRDT
351	ADGTFLVRDASTKMHGDYTLTLRKGGNNKLIKIFHRDGKYGFSDPLTFSS
401	VVELINHYRNESLAQYNPKLDVKLLYPVSKYQQDQVVKEDNIEAVGKKLH
451	EYNTQFQEKSREYDRLYEEYTRTSQEIQMKRTAIEAFNETIKIFEEQCQT
501	QERYSKEYIEKFKREGNEKEIQRIMHNYDKLKSRISEIIDSRRRLEEDLK
551	KQAAEYREIDKRMNSIKPDLIQLRKTR**DL**MWLTQKGVRQKKLNEWLGN
601	ENTEDQYSLVEDDEDLPHHDEKTWNVGSSNRNKAENLLRGKRDGTFLVRE
651	SSKQGCYACSVVVDGEVKHCVINKTATGYGFAEPYNLYSSLKELVLHYQH
701	TSLVQHNDSLNVTLAYPVYAQQRR
