# Supplementary material for: Somatic PIK3R1 mutations in the iSH2 domain are accessible to PI3Kα inhibition
Source: EMBO Mol Med. 2025 May 19;17(7):1556–74. doi: 10.1038/s44321-025-00249-9 (PMC12254339; doi:10.1038/s44321-025-00249-9)
Supplement: Supplementary file 8 — Expanded View Figures [file 44321_2025_249_MOESM8_ESM.pdf]

Expanded View Figures

Figure EV1. Double mutant in cis is associated with a strong stimulation of P-AKT compared to single mutants.

Western blot and quantification of AKT phosphorylation on residue Ser^{473} and S6RP in HeLa cells transfected with plasmids containing either GFP, $PIK3R1^{WT}$, $PIK3R1^{c.1699A>G}$, $PIK3R1^{c.1703C>T}$ or $PIK3R1^{c.1699A>G,\ c.1703C>T}$ variants. In all experiments, cells were stimulated with recombinant human IGF-1 (10 ng/mL) for 30′. Graphs show mean ± SEM from 3 biological replicates in each group. *P*-values were obtained from two-way ANOVAs; exact *p*-values for P-AKT^{S473}/tubulin are $p = 9.35 \times 10^{-5}$ when comparing $PIK3R1^{c.1699A>G,\ c.1703C>T}$ and $PIK3R1^{WT}$; $p = 7.30 \times 10^{-5}$ when comparing $PIK3R1^{c.1699A>G,\ c.1703C>T}$ and $PIK3R1^{c.1703C>T}$. Exact *p*-values for P-AKT^{S473}/total AKT are $p = 1.17 \times 10^{-5}$ when comparing $PIK3R1^{c.1699A>G,\ c.1703C>T}$ and $PIK3R1^{WT}$; $p = 1.09 \times 10^{-5}$ when comparing $PIK3R1^{c.1699A>G,\ c.1703C>T}$ and $PIK3R1^{c.1703C>T}$ Source data are available online for this figure.

 © The Author(s)

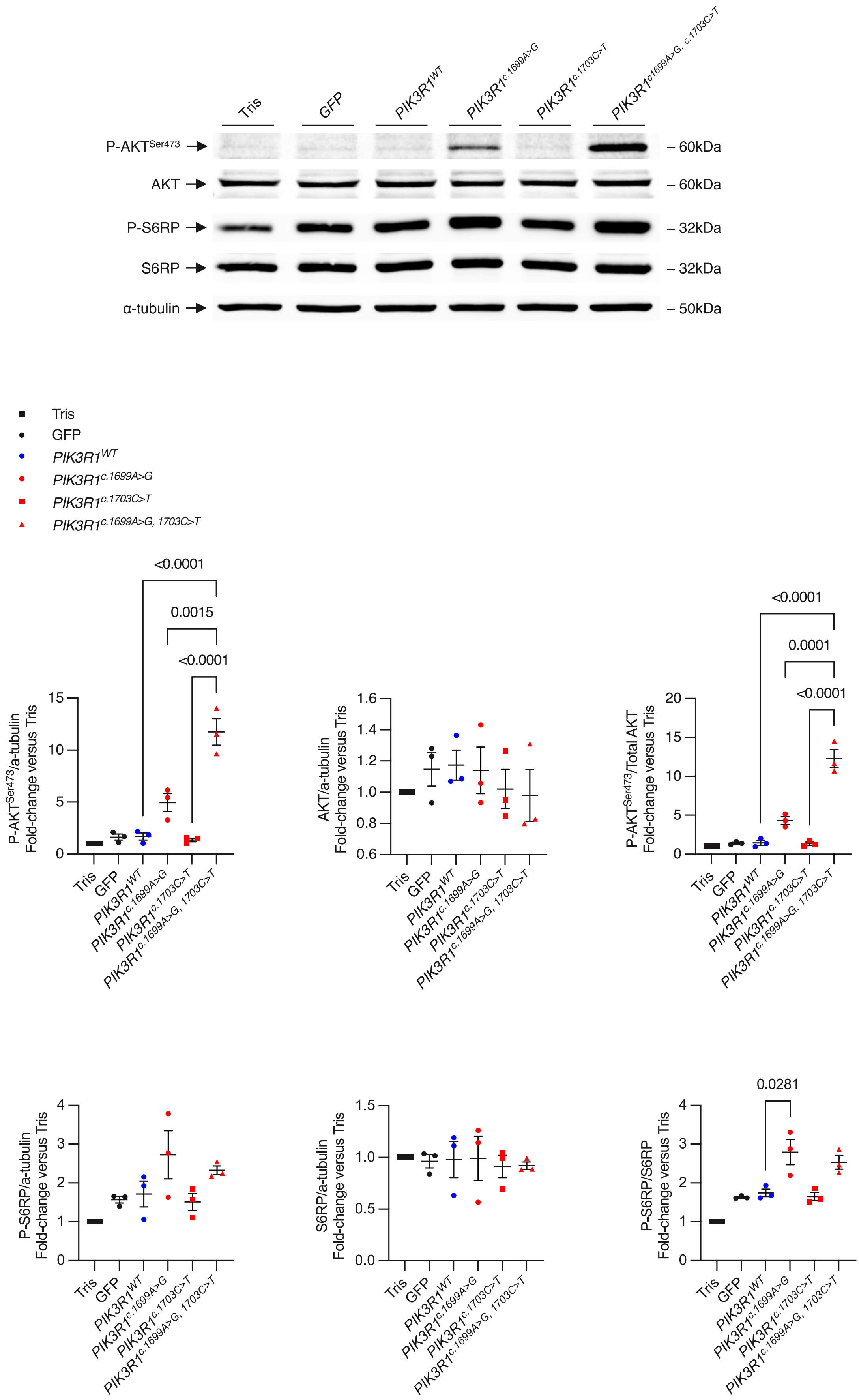
Tris
GFP
PIK3R1WT
PIK3R1c.1699A>G
PIK3R1c.1703C>T
PIK3R1c1699A>G, c.1703C>T
P-AKTSer473
AKT
P-S6RP
S6RP
α-tubulin
– 60kDa
– 60kDa
– 32kDa
– 32kDa
– 50kDa
P-AKTSer473/a-tubulin Fold-change versus Tris
AKT/a-tubulin Fold-change versus Tris
P-AKTSer473/Total AKT Fold-change versus Tris
P-S6RP/a-tubulin Fold-change versus Tris
S6RP/a-tubulin Fold-change versus Tris
P-S6RP/S6RP Fold-change versus Tris
<0.0001
0.0015
<0.0001
0.0001
0.0281

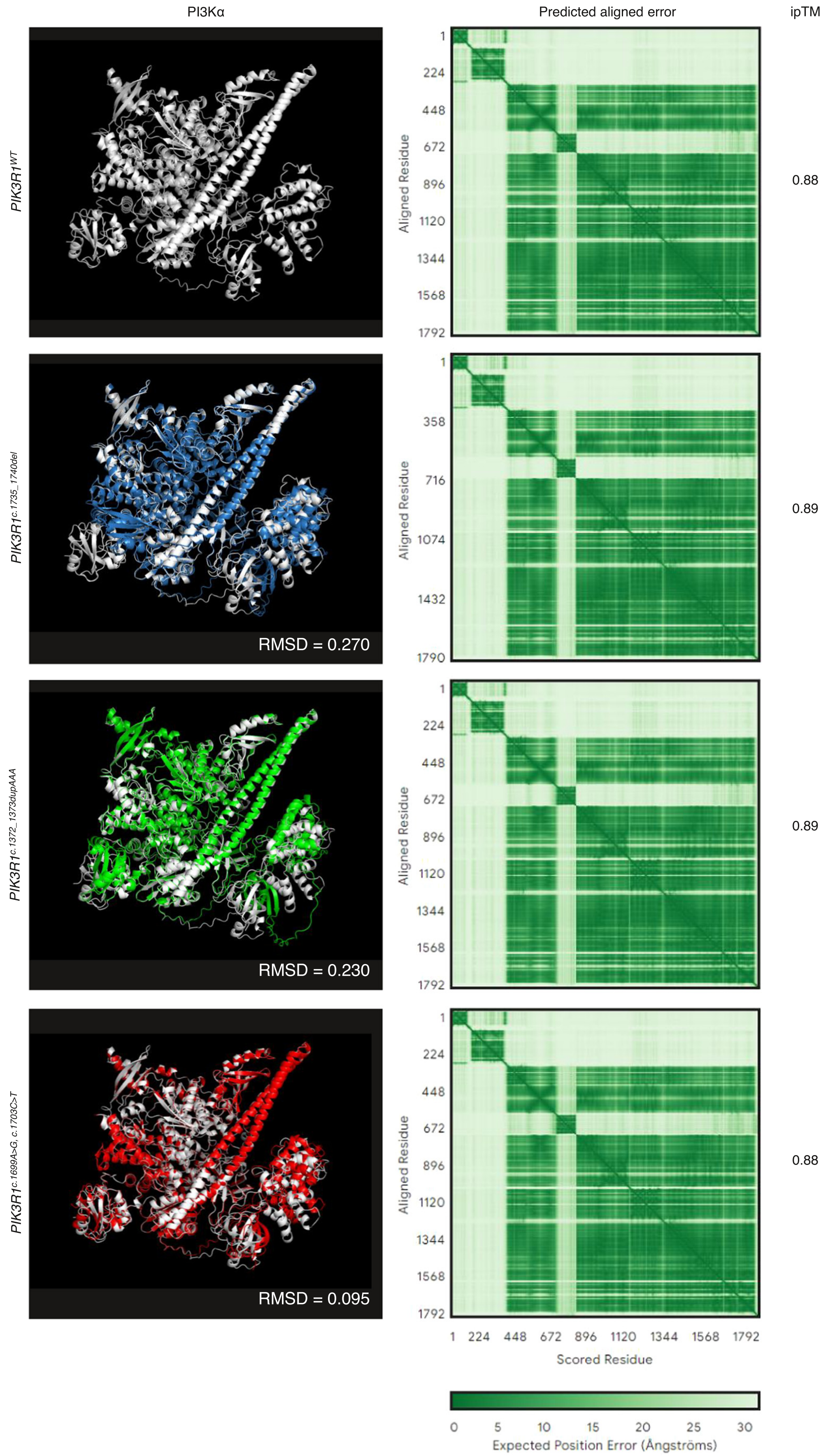
PI3Kα
Predicted aligned error
ipTM
PIK3R1WT
0.88
PIK3R1c.1735_1740del
RMSD = 0.270
0.89
PIK3R1c.1372_1373dupAAA
RMSD = 0.230
0.89
PIK3R1c.1699A>G, c.1703C>T
RMSD = 0.095
0.88
Aligned Residue
Scored Residue
1 224 448 672 896 1120 1344 1568 1792
1 358 716 1074 1432 1790
Expected Position Error (Ångströms)
0 5 10 15 20 25 30

 © The Author(s)

Figure EV2. 3D modeling of PI3Kα resulting from the dimerization of wild-type p110α with the different p85α mutants.

Left panel: 3D structures of PI3Kα containing wild-type p110α and either wild-type p85α or variants (*c.1735_1740del*, *c.1372_1373dupAAA* or *c.1699A>G, c.1703C>T)*, as predicted by AlphaFold3 and formatted with the PyMOL software. The resulting 3D model of each variant was superimposed to that of wild-type PI3Kα for comparison. Root mean square deviation (RMSD) values are shown for each mutant compared to the wild-type dimer. Right panel: Predicted aligned error graphs obtained from AlphaFold3 show high reliability of the predicted models regarding the position of the iSH2 domain of p85α (approx. residues 448 to 672) relative to the p110α subunit (approx. residues 896 to 1792). Interface predicted template modeling (ipTM) scores show highly reliable positioning of the two subunits in the predicted dimers. Source data are available online for this figure.

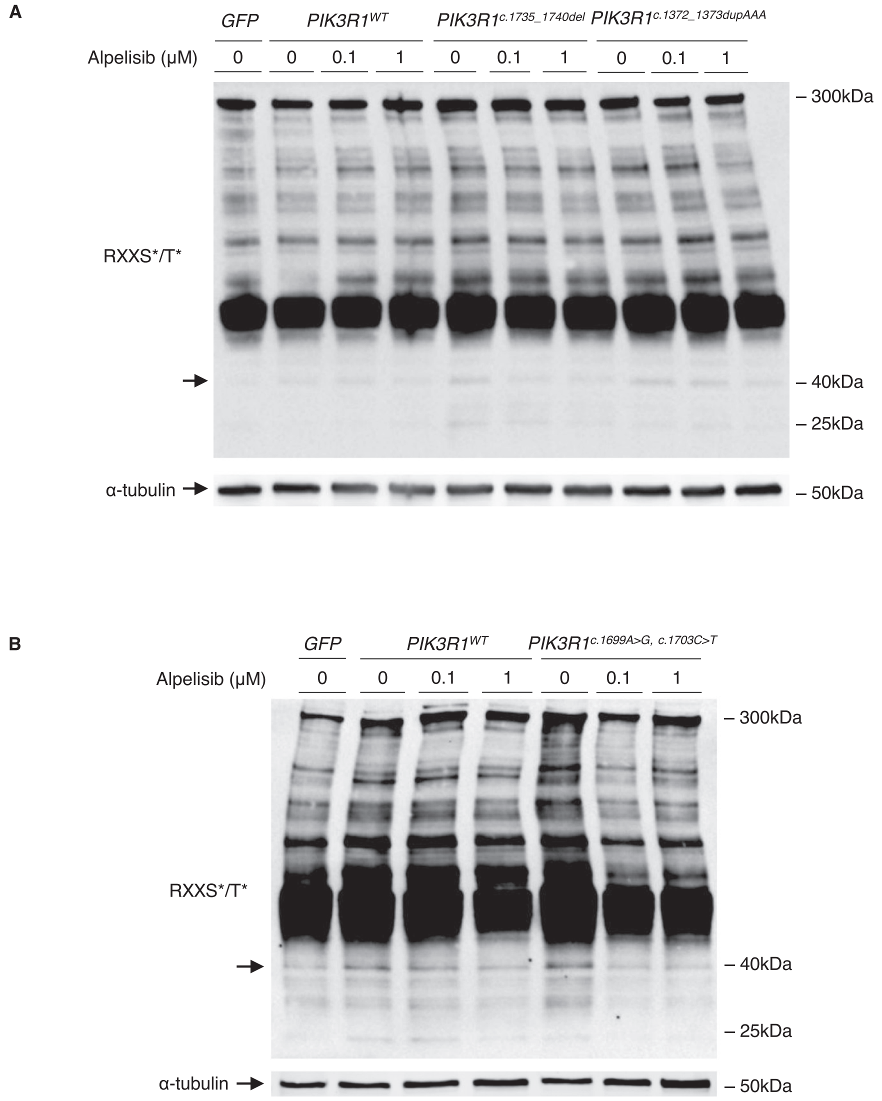

Figure EV3. Phosphorylation profiles of AKT targets identify a 40 kDa effector sensitive to alpelisib in cells transfected with *PIK3R1* variants.

(**A**) Western Blot of proteins phosphorylated on RXXS*/T* residues in HeLa cells transfected with plasmids encoding $PIK3R1^{WT}$, $PIK3R1^{c.1735\_1740del}$ or $PIK3R1^{c.1372\_1373dupAAA}$. (**B**) Western Blot of proteins phosphorylated on RXXS*/T* residues in HeLa cells transfected with plasmids encoding $PIK3R1^{WT}$ or $PIK3R1^{c.1699A>G,\ c.1703C>T}$. In both cases, the phosphorylation levels of a 40 kDa protein increase in cells transfected with *PIK3R1* variants and decrease upon treatment with alpelisib. Source data are available online for this figure.

 © The Author(s)

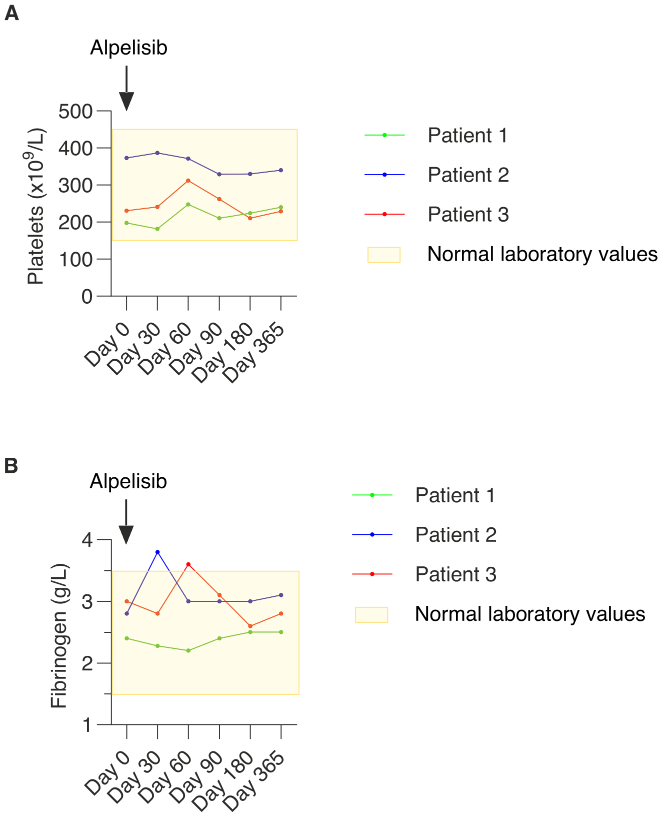

Figure EV4. Patients had normal platelets counts and fibrinogen throughout alpelisib treatment.

(**A**) Platelet counts before and following alpelisib introduction. Graph shows longitudinal data from 3 patients and normal laboratory values. No statistical difference was seen between timepoints according to a Friedman test. (**B**) Fibrinogen levels before and following alpelisib introduction. Graph shows longitudinal data from 3 patients and normal laboratory values. No statistical difference was seen between timepoints according to a Friedman test. Source data are available online for this figure.

© The Author(s)
